# Microarray analysis of gene expression in lung tissues of indium-exposed rats: possible roles of S100 proteins in lung diseases

**DOI:** 10.1007/s00204-024-03897-x

**Published:** 2024-11-08

**Authors:** Yusuke Hiraku, Akiyo Tanaka, Masato Yamamoto, Minori Nakatani, Mayu Kobayashi, Eiki Kimura, Sharif Ahmed, Mariko Murata

**Affiliations:** 1 Department of Environmental Health, University of Fukui School of Medical Science, Eiheiji, Fukui 910-1193 Japan; 2 Department of Environmental and Molecular Medicine, Mie University Graduate School of Medicine, Tsu, Mie Japan; 3 Center for Plasma Nano-Interface Engineering, Kyushu University, Fukuoka, Japan; 4 Graduate School of Health Science, Suzuka University of Medical Science, Suzuka, Mie Japan

**Keywords:** Indium, Microarray, S100 proteins, Inflammation, Lung disease

## Abstract

Indium compounds are used in manufacturing displays of mobile phones and televisions. These compounds cause interstitial pneumonia in workers and lung cancer in animals, but their precise mechanisms are unclear. In this study, we performed microarray analysis of gene expression in lung tissues of indium-exposed rats. Male Wistar rats (8-week-old) were exposed to indium oxide (In_2_O_3_, mean particle diameter 0.14 μm) and indium-tin oxide (ITO, mean particle diameter 0.95 μm) by intratracheal instillation (10 mg indium/kg body weight/instillation) twice a week and five times in total. These rats were sacrificed immediately, 3 weeks and 12 weeks after the last instillation. Hematoxylin and eosin and Masson’s trichrome staining showed that indium compounds induced infiltration of neutrophils and macrophages into alveolar space, and fibrosis around bronchial epithelium and in alveolar wall. Microarray analysis revealed that In_2_O_3_ and ITO significantly upregulated 233 and 676 genes at 12 weeks, respectively (> twofold, *p* < 0.05 by ANOVA + Tukey’s test). In_2_O_3_ and ITO largely upregulated *Lcn2* (lipocalin-2) (49.4- and 91.8-fold), *S100a9* (30.2- and 46.5-fold) and *S100a8* (11.5- and 22.0-fold), respectively. Metascape database predicted that these genes participate in immunomodulatory and inflammatory responses. Real-time PCR confirmed that these genes were upregulated by indium compounds throughout the experiments. In Western blotting, S100A9 expression was significantly increased by indium exposure, whereas LCN2 expression was only slightly increased. Fluorescent immunohistochemistry revealed that S100A9 and S100A8 were expressed in alveolar epithelial cells and neutrophils in indium-exposed rats. These results suggest that S100 proteins contribute to indium-induced lung diseases via neutrophil-mediated inflammatory responses.

**Supplementary Information:**

The online version contains supplementary material available at 10.1007/s00204-024-03897-x.

## Introduction

Indium compounds are used in manufacturing various products in industry. Indium tin oxide (ITO) is a sintered material, consisting of indium oxide (In_2_O_3_) and tin oxide, which accounts for most global indium consumption. ITO is primarily used for electrically conductive purposes to produce a variety of flat-panel displays included in mobile phones and computers, because of high electrical conductivity and transparency (U.S. Geological Survey 2023).

However, indium compounds cause harmful effects on humans and experimental animals. The first case of interstitial pneumonia in an indium-exposed worker occurred in Japan (Homma et al. 2003). An epidemiological study has demonstrated that inhalation exposure to indium compounds is closely associated with interstitial changes in the lung (Chonan et al. 2007). A follow-up study has shown that long-term adverse effects on emphysematous changes occurred in workers with high serum indium concentrations (Nakano et al. 2014). Indium-induced lung disorders also occurred in other countries, including Korea, China and the United States (Choi et al. 2015; Cummings et al. 2010, 2012; Omae et al. 2011). In addition, there is a serious concern about lung carcinogenicity of indium compounds. Animal experiments have demonstrated that long-term inhalation exposure to indium phosphide and ITO caused lung carcinogenesis in rats (Nagano et al. 2011; National Toxicology Program 2001). Based on these findings, the International Agency for Research on Cancer (IARC) classified indium phosphide and ITO as group 2A (IARC 2006) and 2B (IARC 2018) carcinogens, respectively. Recently, a few lung cancer cases have been found in indium-exposed workers, although indium carcinogenicity to humans is still unclear (Nakano et al. 2019).

Inhalation exposure to particulate matters causes chronic inflammation in the respiratory systems, leading to lung diseases, including cancer. We have demonstrated that various particles and fibers, including carbon black, multi-walled carbon nanotube and nanoparticles of indium compounds, caused the formation of 8-nitroguanine, a DNA lesion formed during inflammation, in RAW 264.7 mouse macrophages and A549 human lung epithelial cells (Afroz et al. 2018; Ahmed et al. 2020; Guo et al. 2012; Hiraku et al. 2016, 2017). However, the precise mechanisms of indium-induced lung diseases have not been well understood.

In this study, to clarify molecular mechanism of indium-induced lung diseases, we performed microarray analysis for gene expression in lung tissues of rats exposed to In_2_O_3_ and ITO particles by intratracheal instillation. For significantly upregulated genes by these indium compounds, we performed bioinformatic analysis using Metascape, a web-based portal designed to provide a comprehensive gene list annotation (Zhou et al. 2019). Based on the bioinformatic analysis, we focused on lipocalin-2 (LCN2) and S100 proteins (S100A9 and S100A8), which are known to mediate inflammatory responses and lung diseases. LCN2 is secreted by immune and epithelial cells in various tissues, including lung, and acts as an acute-phase protein that mediates inflammatory responses (Guardado et al. 2021). S100A9 mediates a variety of respiratory diseases, such as chronic obstructive pulmonary disorder (COPD) and infectious diseases, including tuberculosis, influenza and coronavirus disease 2019 (COVID-19) by forming a heterodimer with S100A8 (Mellett and Khader 2022). However, the role of these proteins in indium-induced lung diseases have not been investigated. In this study, we validated the expression of these genes in lung tissues of indium-exposed rats by real-time PCR and Western blotting. We further investigated the distribution of S100 proteins in lung tissues by fluorescent immunohistochemistry to examine the role of these molecules in indium-induced lung diseases.

## Materials and methods

### Test materials

We used In_2_O_3_ and ITO particles for animal experiments as described previously (Tanaka et al. 2010). In_2_O_3_ particles (> 99.99% pure) were purchased from Katayama Chemicals (Osaka, Japan). The mean particle diameter of In_2_O_3_ was 0.14 μm, which was found from the ratio of the surface area of the particle by the Brunauer–Emmett–Teller (BET) adsorption method. ITO particles were kindly donated by a company and contained 74.4% (w/w) indium and 7.8% (w/w) tin, the remainder being oxygen as described previously (Tanaka et al. 2010). The mean particle diameter for ITO was 0.95 μm (geometric standard deviation: 2.42), as measured by an image analyzer (Nikon Co. Ltd, Tokyo Japan) using scanning electron microscopy (T-220, JEOL Ltd, Tokyo, Japan). ITO particles were analyzed in an energy dispersive X-ray fluorescence element analyzer (MESA-500, Horiba Ltd., Kyoto, Japan), and contained 0.16% (w/w) zirconium and 0.02% (w/w) silicon.

### Animal experiments

Eighty one male Wistar rats were purchased from Kyudo. Co. (Tosu, Japan) at 6 weeks of age and housed under temperature conditions of between 22 and 25 °C. Animal studies were conducted in accordance with the Guidelines for Animal Experiments in the Graduate School of Medical Sciences, Kyushu University. All rats were maintained under a cycle of 12-h lighting, within a specific pathogen-free laboratory room at the Laboratory of Animal Experiments, Graduate School of Medical Sciences, Kyushu University. These rats were provided with a commercial diet (CE-2 pellets, Clea Japan Inc., Tokyo, Japan) and tap water ad libitum. After an acclimatization period of 2 weeks, intratracheal instillations of indium compounds were started at 8 weeks of age. In_2_O_3_ or ITO particles were suspended in distilled water and given to rats by intratracheal instillation (10 mg indium/kg body weight/instillation) twice a week and five times in total. Control rats only received 1 ml/kg body weight of distilled water intratracheally. Nine rats in each group were euthanized by carbon dioxide gas immediately (0 weeks), 3 weeks and 12 weeks after the last instillation to detect molecular events at an early stage, and lower lobes of lung tissues were used for the experiments. Lung tissues obtained from four rats were fixed in 10% neutral buffered formalin and embedded in paraffin. Then the tissues were cut at a thickness of 6 μm and used for histopathological analysis and immunohistochemistry. Lung tissues obtained from five rats were stored at -80 °C and used for microarray analysis, real-time PCR and Western blotting.

### Histological staining

To observe histopathological changes in lung tissues of indium-exposed rats, we performed hematoxylin and eosin (HE) staining. Fibrotic changes in the lung tissues were evaluated by Masson’s trichrome staining using a Trichrome Stain Kit (ScyTek Labolatories, Logan, UT, USA) and aniline blue (Fujifilm Wako Pure Chemical Corporation, Osaka, Japan) as reported previously (Hiraku et al. 2021).

### Microarray analysis

Microarray analysis for gene expression in lung tissues of indium-exposed rats at 12 weeks was performed as described previously (Hiraku et al. 2021). The lung tissues were preserved in RNA*later*-ICE (Ambion, Carlesbad, CA, USA) at − 20 °C and homogenized in Lysis/Binding Buffer included in a *mir*Vana miRNA Isolation Kit (Ambion) using a μT-01 Beads Crusher (Taitec, Koshigaya, Japan). Total RNA was extracted from the homogenates with this kit following manufacturer’s instructions and quantified with a BioSpec-nano UV–VIS spectrophotometer (Shimadzu, Kyoto, Japan). RNA was reverse-transcribed to cDNA and then RNA was synthesized using a Low Input Quick Amp Labeling Kit (Agilent Technologies) according to manufacturer’s instruction. cRNA (400 ng) was labeled with Cy3 and then hybridized onto SurePrint G3 Rat Gene Expression Microarrays (Agilent Technologies) at 65 °C for 17 h. Hybridized microarray slides were washed and scanned with a DNA Microarray Scanner (Agilent Technologies). The scanned images were analyzed numerically using a Feature Extraction software (version 10.7, Agilent Technologies). Microarray data were statistically analyzed with a GeneSpring GX software (version 12, Agilent Technologies). The data were normalized by 75% percentile shift according to previous studies (Hiraku et al. 2021). We performed analysis of variance (ANOVA) followed by Tukey’s test and identified differentially expressed genes with > twofold change between the control and indium-exposed rats (*n* = 4). *P* values less than 0.05 were considered as statistically significant.

### Bioinformatic analysis

Based on microarray analysis, we performed bioinformatic analysis for significantly upregulated genes by indium compounds using Metascape database (Zhou et al. 2019). We carried out gene ontology (GO) enrichment analysis and protein–protein interaction (PPI) network analysis according to the workflow, including ID conversion, annotation, membership and enrichment. For enrichment analysis, terms with a *p*-value < 0.01, a minimum count of 3, and an enrichment factor > 1.5 were collected and grouped into clusters based on their membership similarities. *P* values were calculated based on the cumulative hypergeometric distribution.

### Real-time PCR

To validate the gene expression levels in the lung tissues of indium-exposed rats, we performed quantitative real-time PCR as described previously (Hiraku et al. 2021). Total RNA was extracted from the lung tissues of indium-exposed rats and quantified as described above. RNA was reverse-transcribed with a High Capacity RNA-to-cDNA kit (Applied Biosystems). In all assays, TaqMan predeveloped master mix (Applied Biosystems) was used. RNA levels were normalized against the corresponding levels of *Actb* gene (β-actin). Relative expression levels were calculated using the ΔΔCt method. The experiments were performed in duplicate in separate wells.

### Western blotting

We examined the expression levels of LCN2 and S100A9 in lung tissues of indium-exposed rats by Western blotting as described previously (Hiraku et al. 2016, 2021). The lung tissues were homogenized in phosphate-buffered saline (pH 7.4) containing proteinase inhibitor cocktail (Complete Mini, Roche Diagnostics, Mannheim, Germany) with a μT-01 Beads Crusher (Taitec). The homogenate was centrifuged at 14,000×*g* for 10 min at 4 °C, and the supernatant was used for the experiment. Protein concentration was measured using a Coomassie Protein Assay Reagent Kit (Pierce Biotechnology, Rockford, USA) following manufacturer’s instruction. Proteins were solubilized in sample buffer [2% (w/v) sodium dodecyl sulfate (SDS), 10% (v/v) glycerol, 5% (v/v) 2-mercaptoethanol, 0.001% (w/v) bromophenol blue and 50 mM Tris–HCl (pH 6.8)] and boiled for 5 min. The proteins were separated by 15% SDS-PAGE and blotted onto a polyvinylidene difluoride membrane, which was then treated with 5% (w/v) skim milk in Tris-buffered saline (pH 7.4) containing 0.1% (v/v) Tween 20. The membrane was incubated with rabbit polyclonal anti-LCN2 antibody (ab63292, 1:1,000, Abcam), rabbit polyclonal anti-S100A9 antibody (ab75478, 1:500, Abcam) or mouse monoclonal anti-β-actin antibody (sc-47778, 1:2,000, an internal standard, Santa Cruz Biotechnology, Santa Cruz, CA, USA) for 60 min, and then with horseradish peroxidase-conjugated donkey anti-rabbit IgG or goat anti-mouse IgG antibody (1:10,000 each, Santa Cruz Biotechnology) for 30 min. The membrane was treated with ECL plus Western blotting detection reagents (GE Healthcare, Backinghamshire, UK) and analyzed with a LAS-4000 mini biomolecular imager (Fujifilm, Tokyo, Japan). We performed quantitative image analysis by measuring the band intensity with an ImageJ software and normalized with β-actin.

### Immunohistochemical analysis

To examine the localization of S100 family proteins in lung tissues of indium-exposed rats, we performed fluorescent immunohistochemical analysis as described previously (Hiraku et al. 2014). Paraffin sections of the lung tissues were deparaffinized, microwaved and incubated with rabbit monoclonal anti-S100A9 antibody (ab242945, 1:500, Abcam) or rabbit polyclonal anti-S100A8 antibody (ab180735, 1:200, Abcam) overnight at room temperature. Then, the sections were incubated with Alexa 594-labeled goat antibody against rabbit IgG (1:400, Molecular Probes Inc., Eugene, OR, USA) for 3 h. The nuclei were stained with 4',6-diamidino-2-phenylindole (DAPI) contained in SlowFade Diamond (Molecular Probes). The stained sections were examined under a fluorescent microscope (BX53, Olympus, Tokyo, Japan). To examine the expression levels of these proteins, the staining intensity of each sample was quantified with an ImageJ software by analyzing five randomly selected fields as described previously (Hiraku et al. 2021). To examine the distribution of these proteins, we stained the treated tissue sections by Masson’s trichrome staining.

### Statistical analysis

For statistical analysis of the data, ANOVA followed by Tukey’s test was used at a significance level of 0.05. The statistical analysis was performed by IBM SPSS Statistics version 26 for Macintosh. The data represent means ± SD.

## Results

### Histopathological changes in lung tissues of indium-exposed rats

**Fig. 1:**
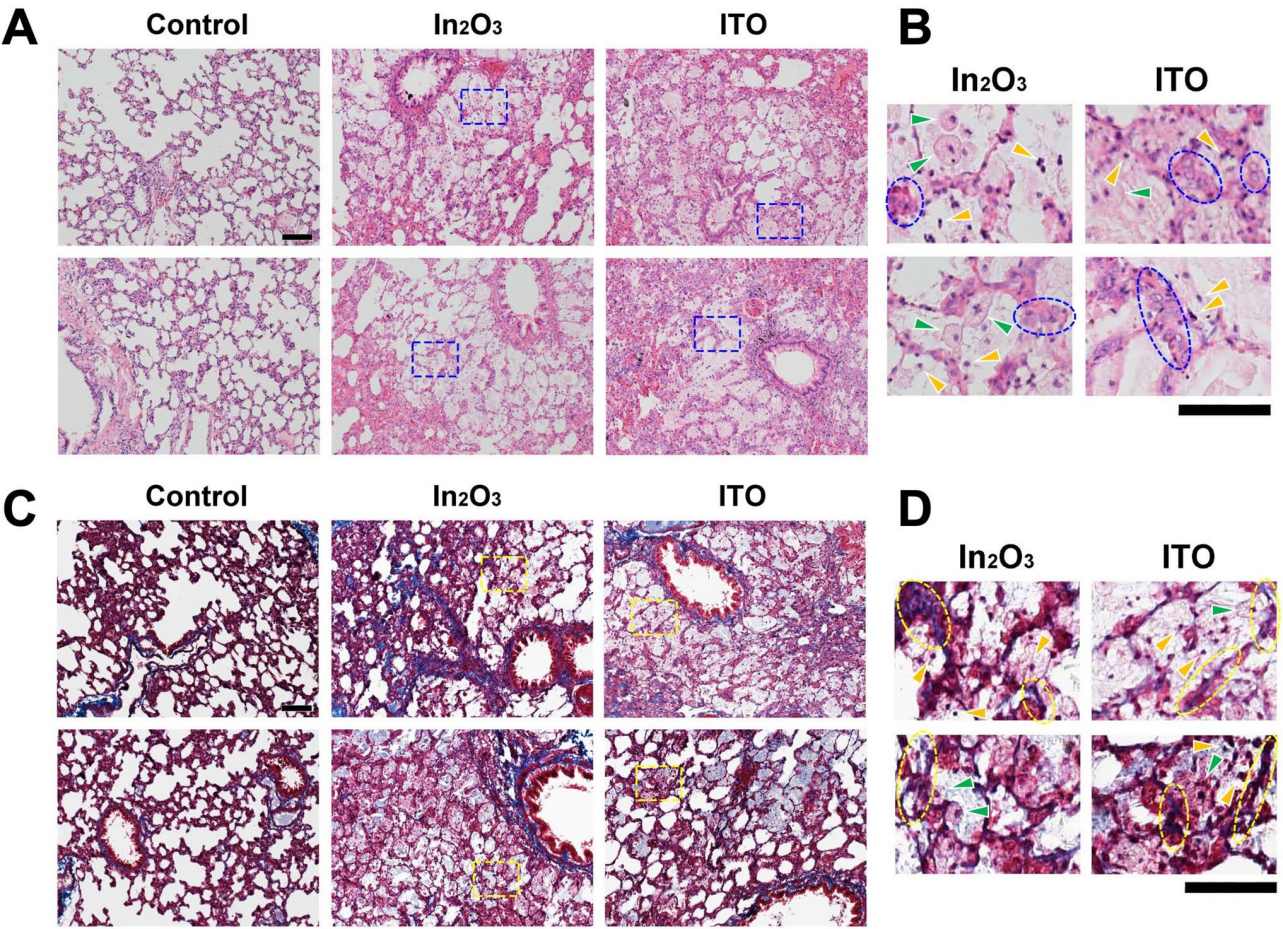
Histopathological changes in lung tissues of indium-exposed rats. Histopathological changes in lung tissues of indium-exposed rats were evaluated by HE staining (**A**, enlarged images are shown in **B**) at 12 weeks as described in Materials and methods. Blue boxes in **A** are shown as enlarged images in **B**. **B** *Yellow arrowheads*, neutrophils; *green arrowheads*, macrophages: *blue circles*, hyperplasia of alveolar type II cells. Scale bars = 100 µm. Fibrotic changes in the lung tissues were evaluated by Masson’s trichrome staining (**C**, enlarged images are shown in **D**) at 12 weeks. Yellow boxes in **C** are shown as enlarged images in **D**. **D** *Yellow arrowheads*, neutrophils; *green arrowheads*, macrophages: *yellow circles*, fibrotic changes. Scale bars = 100 µm (color figure online)

Figure 1 shows histopathological changes in lung tissues of indium-exposed rats at 12 weeks evaluated by HE and Masson’s trichrome staining. Both In_2_O_3_ and ITO caused severe histopathological changes including alveolar wall thickening, infiltration of inflammatory cells, mainly neutrophils and macrophages, into alveolar spaces, hyperplasia of alveolar type II epithelial cells and alveolar proteinosis as previously demonstrated in hamsters (Tanaka et al. 2010) (Fig. 1A, B). Carcinogenic changes were not observed under these conditions. Masson’s trichrome staining shows that both In_2_O_3_ and ITO induced fibrotic changes especially around bronchiolar epithelium and in alveolar walls (Fig. 1C,D).

### Differentially expressed genes in lung tissues of indium-exposed rats in microarray analysis

**Fig. 2:**
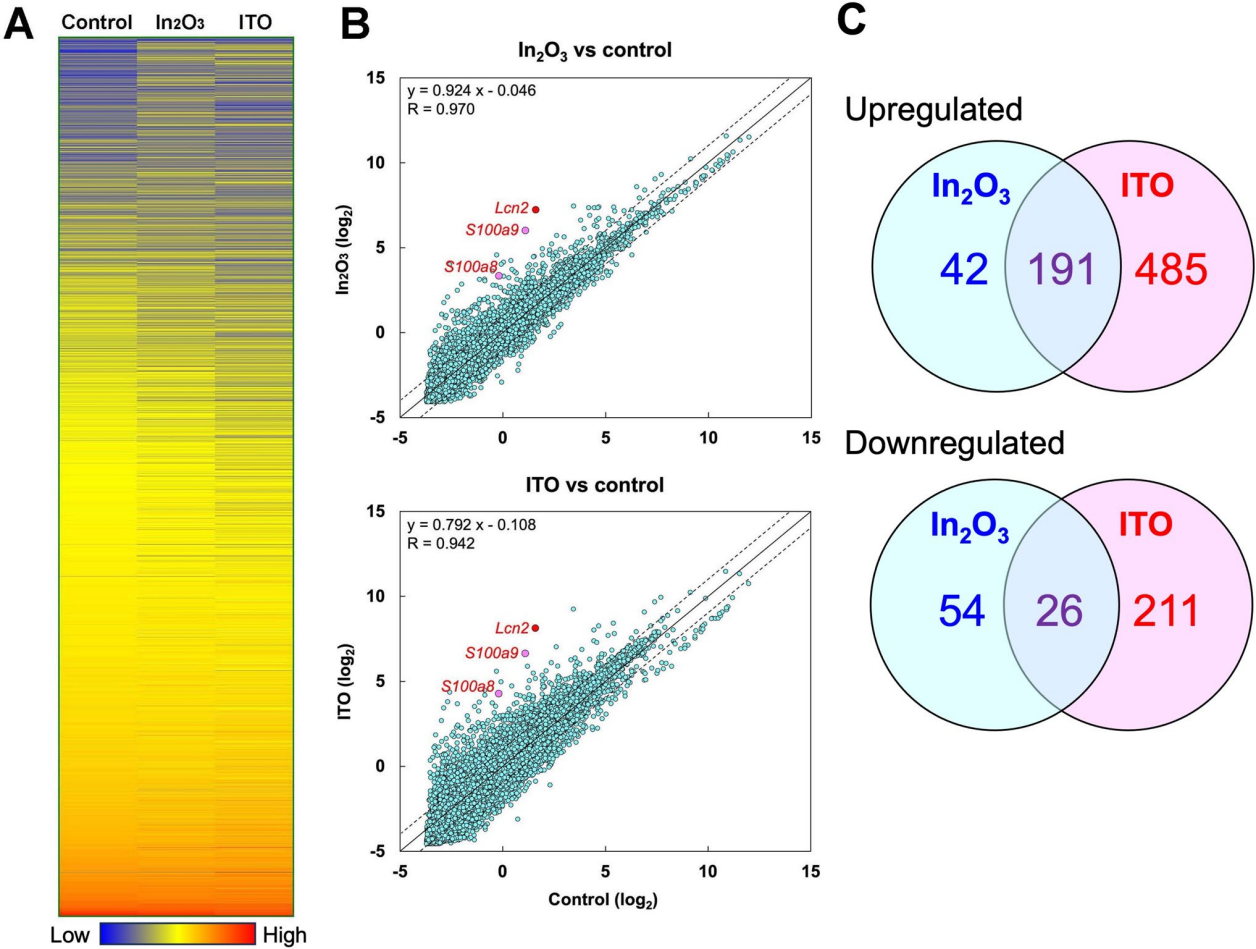
Differential transcriptomic signature in lung tissues of control and indium-exposed rats. **A** Heatmap of microarray analysis of gene expression in lung tissues of indium-exposed rats. **B** Scatter plot for transcriptome comparison of gene profiles. Individual points show the expression level of each gene. Gene expression levels are log_2_-transformed. Solid line indicates same expression level (fold change = 1) and broken lines indicate twofold increase or decrease in gene expression in indium-exposed rats compared with the control rats. The expression levels of *Lcn2* (red), *S100a9* and *S100a8* (pink) are indicated. **C** Venn diagram showing the numbers of significantly upregulated and downregulated genes (> twofold, *p* < 0.05 by ANOVA + Tukey’s test) in lung tissues of In_2_O_3_- and ITO-exposed rats according to Metascape analysis

We performed microarray analysis to examine gene expression in lung tissues of indium-exposed rats at 12 weeks in association with the histopathological changes as described above. Figure 2 shows genome-wide expression profiles in lung tissues of indium-exposed rats. A heatmap (Fig. 2A) and scatter plots (Fig. 2B) revealed differential gene expression, which discriminate among control and indium-exposed rats. Gene expression levels are basically correlated between control and In_2_O_3_-exposed rats (*R* = 0.970, *p* < 0.001 by Pearson’s correlation test) and between control and ITO-exposed rats (*R* = 0.942, *p* < 0.001) (Fig. 2B). Table 1 shows top 10 upregulated genes in the lung tissues of indium-exposed rats. In_2_O_3_ and ITO largely upregulated *Lcn2* (49.4- and 91.8-fold), *S100a9* (30.2- and 46.5-fold) and *S100a8* (11.5- and 22.0-fold), respectively. All significantly upregulated and downregulated gene identifiers in indium-exposed rats are shown in Supplementary Tables S1 and S2, respectively (> twofold, *p* < 0.05 by ANOVA + Tukey’s test). We further performed bioinformatic analysis for these gene identifiers using Metascape database, in which these identifiers are first converted into their corresponding *R. norvegicus* Entrez gene IDs (updated on May 1, 2024). If multiple identifiers correspond to the same Entrez gene ID, they were considered as a single Entrez gene ID. Metascape analysis revealed that in In_2_O_3_- and ITO-exposed rats, the expression levels of 313 genes (233 upregulated and 80 downregulated genes) and 913 genes (676 upregulated and 237 downregulated genes) were differentially expressed compared with control rats, respectively. Among them, 191 genes were upregulated and 26 genes were downregulated by both In_2_O_3_ and ITO (Fig. 2C).

**Table 1:** Top 10 upregulated genes in the lung tissues of indium-exposed rats evaluated by microarray analysis

Gene	In_2_O_3_		Gene	ITO	
	*p* (ANOVA)	Fold increase (vs control)		*p* (ANOVA)	Fold increase (vs control)
***Lcn2***	< 0.0001	49.35	***Lcn2***	< 0.0001	91.77
***S100a9***	< 0.0001	30.23	*Trem2*	< 0.0001	60.01
*Trem2*	< 0.0001	29.87	*Chia*	< 0.0001	56.87
*Hmox1*	0.0004	17.64	***S100a9***	< 0.0001	46.46
*Lilrb4*	0.0002	17.57	*Bpifb1*	0.0004	41.46
*Bag3*	0.0006	17.55	*Lilrb4*	0.0002	33.91
*Bpifb1*	0.0004	16.49	*Gpnmb*	< 0.0001	25.87
*Chia*	< 0.0001	16.40	***S100a8***	< 0.0001	21.97
*Zfand2a*	0.0002	14.73	*C3*	0.0002	21.66
***S100a8***	< 0.0001	11.45	*Ly6i*	< 0.0001	19.76

### Bioinformatic analysis of upregulated genes in lung tissues of indium-exposed rats

**Fig. 3:**
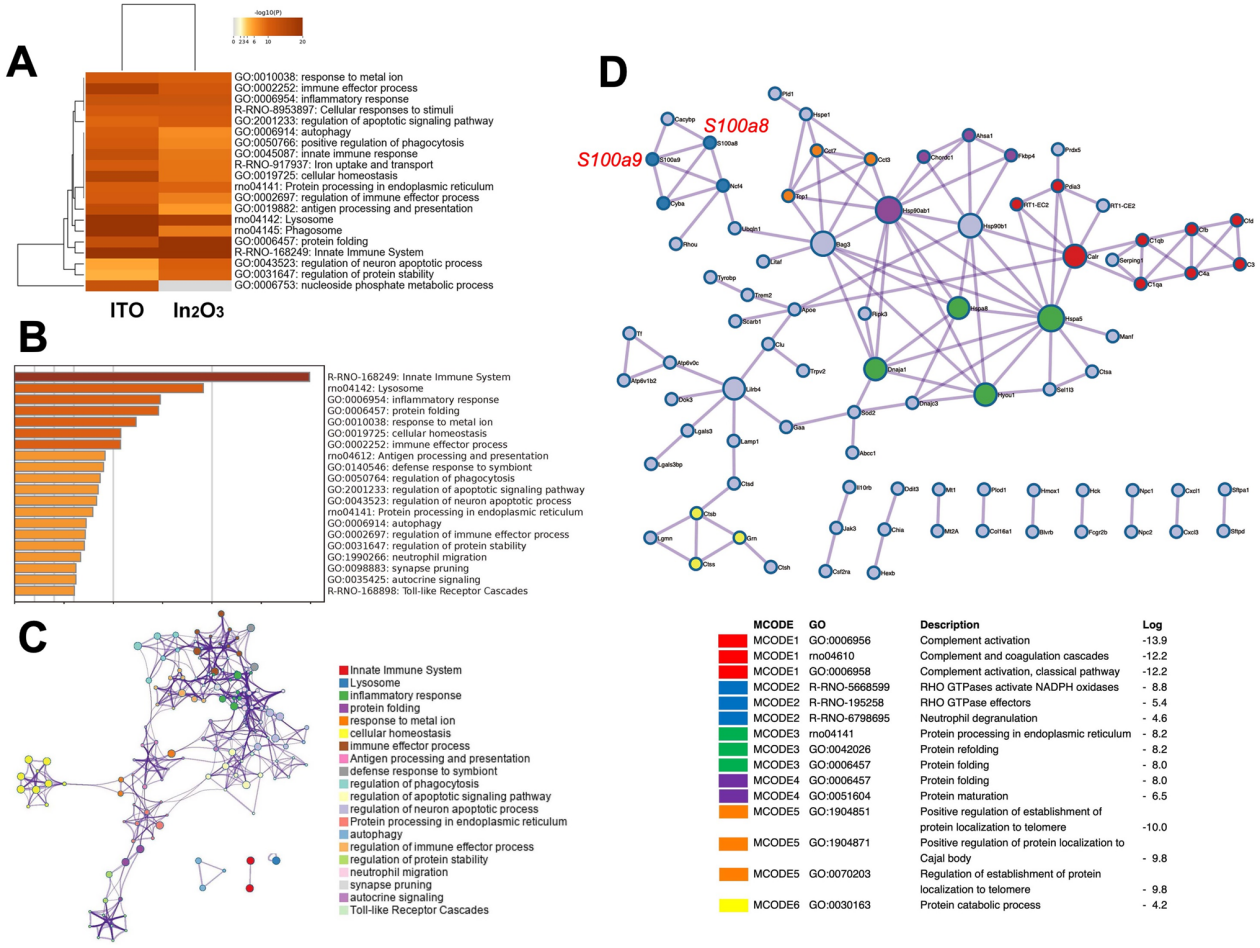
Bioinformatic analysis for upregulated genes in lung tissues of indium-exposed rats. We performed analyses of GO enrichment and protein–protein interaction (PPI) network using Metascape database as described in Materials and methods. **A** Heatmap of enriched terms across genes upregulated by indium compounds. We performed GO enrichment analysis for genes upregulated by In_2_O_3_ (233 genes) and ITO (676 genes). This heatmap shows the top 20 enrichment clusters using a discrete color scale to represent statistical significance. Gray color shows a lack of significance. We also performed analyses of GO enrichment and PPI network for 191 genes upregulated by both In_2_O_3_ and ITO (**B**–**D)**. **B** Bar graph of enriched terms across genes upregulated by both indium compounds. This graph shows enrichment clusters using a discrete color scale to represent statistical significance. **C** Enrichment network visualization showing intra- and inter-cluster similarities of enriched terms. Each term is represented by a circle node, where its size is proportional to the number of input genes fall under that term, and its color represent its cluster identity. **D** PPI network among input genes and MCODE components identified in these genes

We performed bioinformatic analysis for significantly upregulated genes in lung tissues of indium-exposed rats by using Metascape database. Figure 3A shows a heatmap of top 20 enrichment clusters obtained by GO analysis for 233 and 676 upregulated genes by In_2_O_3_ and ITO, respectively. Among the enrichment clusters, R-RNO-168249 (innate immune system), rno04142 (lysosome), GO:0002252 (immune effector process) and many other clusters are common to In_2_O_3_- and ITO-exposed rats. Supplementary Fig. S1 shows top 100 enriched clusters obtained by GO analysis.

We further performed enrichment analyses for 191 upregulated genes common to In_2_O_3_- and ITO-exposed rats. Figure 3B shows a bar graph of enrichment clusters across these genes, which primarily include immunomodulatory and inflammatory responses, such as in R-RNO-168249 (innate immune system), rno04142 (lysosome), GO:0006954 (inflammatory response) and GO:0002252 (immune effector process). An enrichment network showing the intra- and inter-cluster similarities of enriched terms is shown in Fig. 3C. A PPI network formed by upregulated genes by both indium compounds is shown in Fig. 3D. According to molecular complex detection (MCODE), S100A9 and S100A8 are predicted to interact with each other and contribute to neutrophil degradation. In addition, many complements and molecules involved in protein folding are predicted to interact with multiple proteins.

Table 2 shows comparison of the enrichment of top 4 genes (*Lcn2*, *S100A9*, *Trem2*, *Hmox1* and *Chia*) and *S100A8* upregulated by In_2_O_3_ and/or ITO. *Lcn2*, *S100A9* and *S100A8* share common properties, including R-RNO-168249 (innate immune system), GO:0006954 (inflammatory response), GO:0010038 (response to metal ion), GO-1990266 (neutrophil migration) and R-RNO-168898 (Toll-like receptor cascades). Other upregulated genes, *Trem2*, *Hmox1* and *Chia* showed different patterns of gene enrichment. From the results of these analyses, we examined the expression of* Lcn2*, *S100A9* and *S100A8* in further experiments.

**Table 2:** Comparison of enrichment analysis of top 4 upregulated genes in lung tissues of In_2_O_3_- and/or ITO-exposed rats

Term	Description	LogP	*Lcn2*	*S100a9*	*S100a8*	*Trem2*	*Hmox1*	*Chia*
R-RNO-168249	Innate immune system	− 29.9	+	+	+			
GO:0006954	Inflammatory response	− 14.8	+	+	+		+	+
GO:0010038	Response to metal ion	− 12.3	+	+	+		+	
GO:0019725	Cellular homeostasis	− 10.8	+			+	+	
GO:0002252	Immune effector process	− 10.7		+	+	+		
GO:0140546	Defense response to symbiont	− 9.0	+	+	+			
GO:2001233	Regulation of apoptotic signaling pathway	− 8.5	+	+	+	+	+	
GO:0043523	Regulation of neuron apoptotic process	− 8.3	+			+	+	
GO:0006914	Autophagy	− 7.3		+	+		+	
GO:1990266	Neutrophil migration	− 6.7	+	+	+	+		
GO:0035425	Autocrine signaling	− 6.2	+	+	+			
R-RNO-168898	Toll-like receptor cascades	− 6.1	+	+	+			

### Validation of gene expression by real-time PCR

**Fig. 4:**
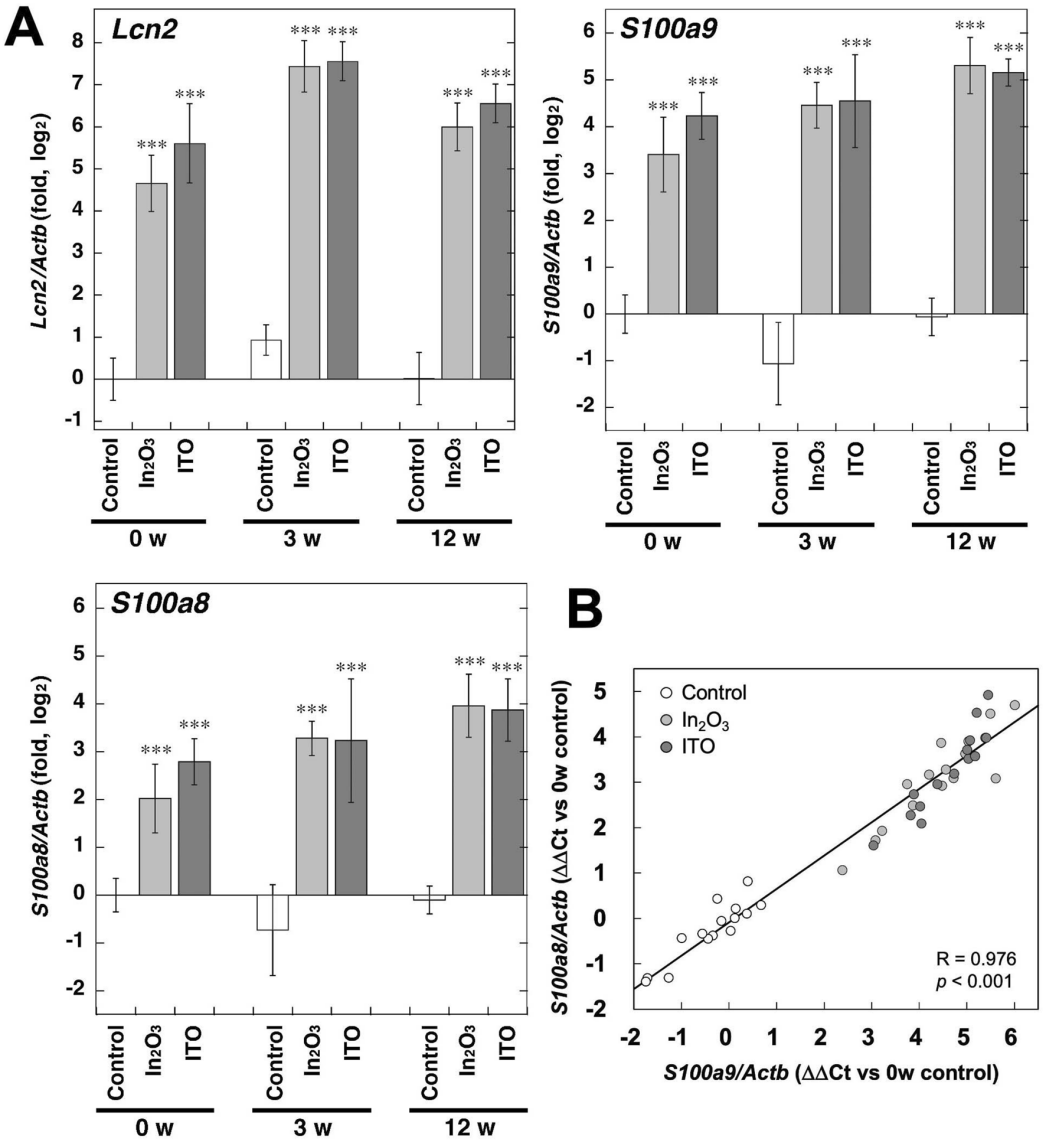
Gene expression of *Lcn2* and S100 proteins in lung tissues of indium-exposed rats. **A** Gene expression levels in lung tissues. Expression levels of *Lcn2*, *S100a9* and *S100a8* were evaluated by real-time PCR as described in Materials and methods. Data were expressed as means ± SD of 4–5 independent experiments. ***p < 0.001, compared with control by ANOVA followed by Tukey’s test. **B** Correlation of the expression levels of *S100a9* and *S100a8*. Individual points show the expression levels of *S100a9* and *S100a8* in each lung tissue of control (white), In_2_O_3_-exposed (pale gray) and ITO-exposed (dark gray) rats at 0, 3 and 12 weeks after the last instillation. The correlation of their expression levels was analyzed by Pearson’s correlation test

To validate the results of microarray analysis, we performed real-time PCR to quantify the expression levels of *Lcn2, S100A9* and *S100A8*. The expression levels of these genes were significantly increased by indium exposure (*p* < 0.001, Fig. 4A). In_2_O_3_ increased *Lcn2* expression level by 25.1-, 173.7- and 63.9-fold at 0, 3 and 12 weeks, compared with the control at each time point, respectively. ITO increased *Lcn2* expression level by 49.0-, 188.2- and 94.3-fold at 0, 3 and 12 weeks, respectively (Fig. 4A). In_2_O_3_ increased *S100a9* expression level by 10.6-, 45.8- and 41.4-fold at 0, 3 and 12 weeks, compared with the control, respectively. ITO increased *S100a9* expression level by 18.7-, 49.0- and 37.4-fold at 0, 3 and 12 weeks, respectively (Fig. 4A). In_2_O_3_ increased *S100a8* expression level by 4.1-, 16.1- and 16.6-fold at 0, 3 and 12 weeks, compared with the control, respectively. ITO increased *S100a8* expression level by 6.9-, 15.5- and 15.7-fold at 0, 3 and 12 weeks, respectively (Fig. 4A). The expression levels of *S100a9* and *S100a8* were significantly correlated throughout the experiment (R = 0.976, *p* < 0.001, Fig. 4B), suggesting that *S100a9* and *S100a8* were expressed in parallel and possibly form a heterodimer to exert a crucial function.

### Expression levels of LCN2 and S100A9 in lung tissues at the protein level

**Fig. 5:**
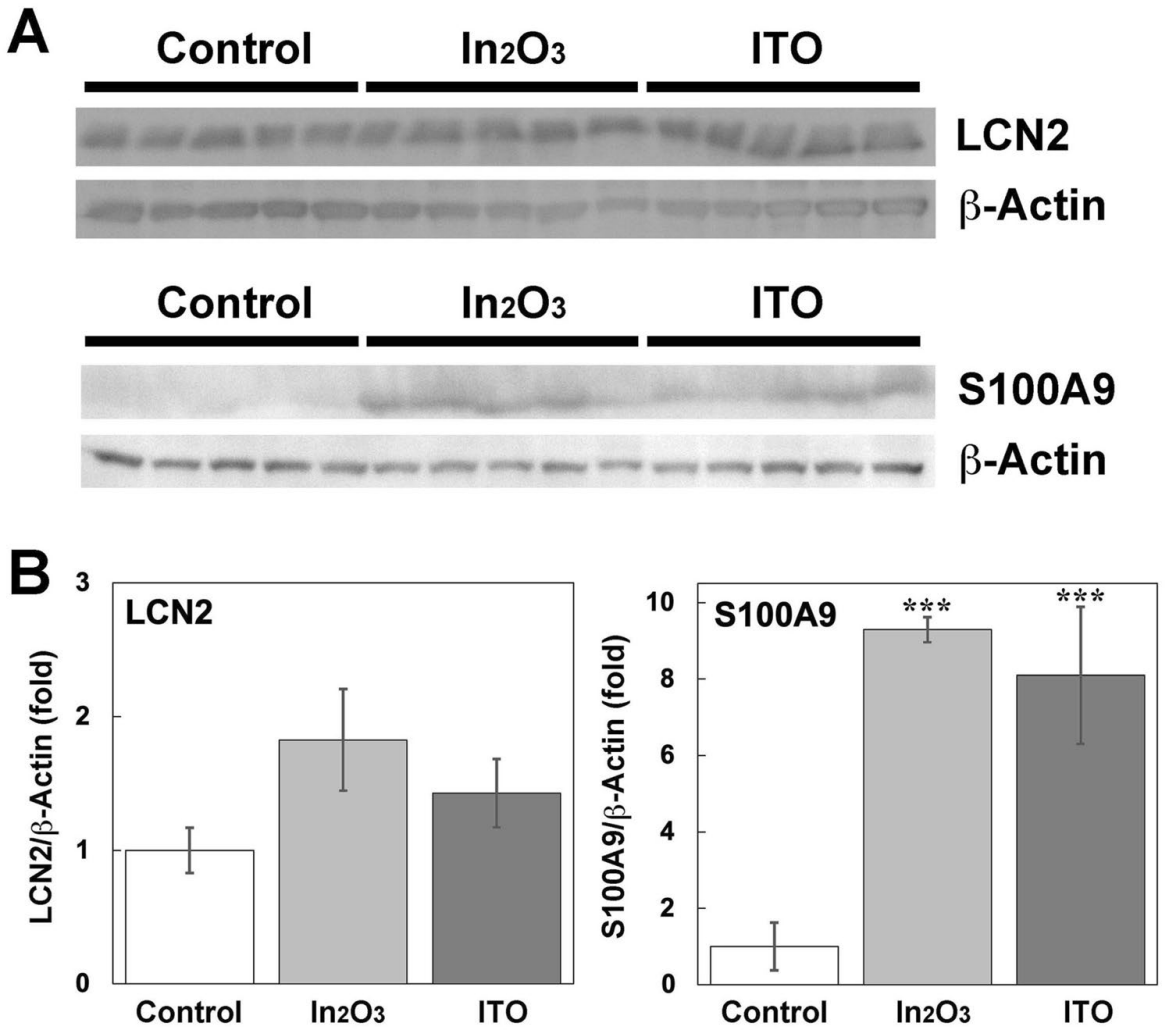
Expression of LCN2 and S100A9 in lung tissues of indium-exposed rats at the protein level. **A** Expression of LCN2 and S100A9 in lung tissues at 12 weeks evaluated by Western blotting. Western blotting was performed as described in Materials and methods. **B** Quantitative image analysis for expression of LCN2 and S100A9. Data were expressed as means ± SD of 5 independent experiments. ***p < 0.001, compared with control by ANOVA followed by Tukey’s test

Figure 5 shows the expression levels of LCN2 and S100A9 in lung tissues of indium-exposed rats evaluated by Western blotting. Exposure to In_2_O_3_ and ITO for 12 weeks appeared to increase LCN2 expression compared to the control rats (Fig. 5A), but quantitative image analysis revealed that there was no statistical significance (Fig. 5B). On the other hand, these indium compounds largely increased S100A9 expression (Fig. 5A), and image analysis showed that these indium compounds significantly increased the intensity of S100A9 expression compared with control (*p* < 0.001, Fig. 5B). There was no significant difference in the intensity of S100A9 expression between In_2_O_3_- and ITO-exposed rats.

### Distribution of S100 proteins in lung tissues

**Fig. 6:**
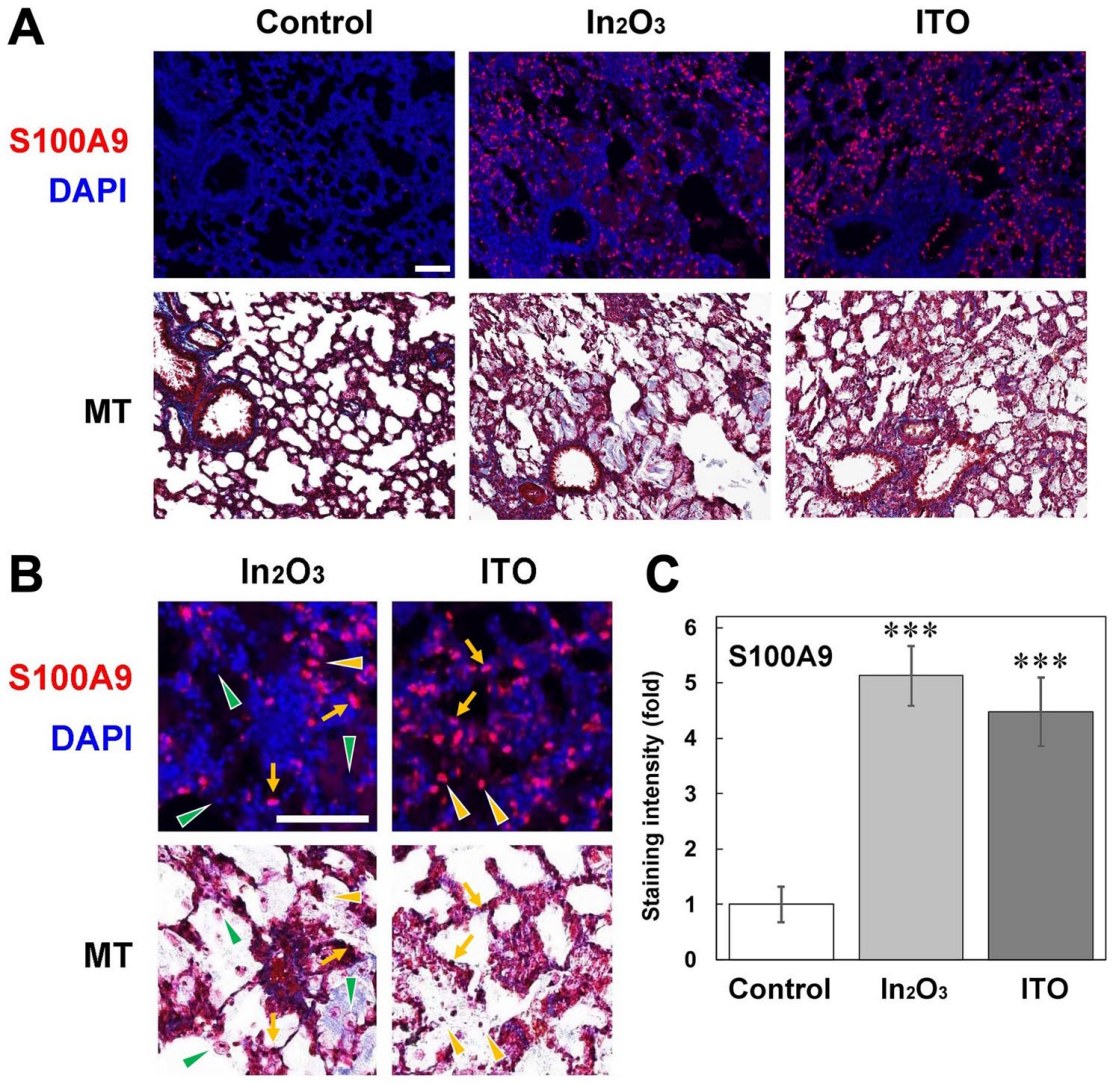
Fluorescent immunohistochemical analysis for S100A9 in lung tissues of indium-exposed rats. **A** Fluorescent images for S100A9 in lung tissues of indium-exposed rats at 12 weeks. Fluorescent immunohistochemistry was performed as described in Materials and methods. The nuclei were stained with DAPI. Histopathological changes were examined by Masson’s trichrome (MT) staining. Scale bar = 100 µm. **B** Enlarged images of fluorescent immunohistochemical analysis for S100A9. *Yellow arrows*, alveolar epithelial cells; *yellow arrowheads*, neutrophils; *green arrowheads*, macrophages. Scale bar = 100 µm. **C** Quantitative image analysis for expression of S100A9. The staining intensity per field was analyzed with an ImageJ software as described in Materials and methods. Data were expressed as means ± SD of 3 independent experiments. ***p < 0.001, compared with control by ANOVA followed by Tukey’s test

Figure 6 shows the distribution of S100A9 expression in lung tissues of indium-exposed rats, evaluated by fluorescent immunohistochemical analysis. No or weak S100A9 expression was observed in lung tissues of control rats, whereas S100A9 was strongly expressed in lung tissues of In_2_O_3_- and ITO-exposed rats (Fig. 6A). In indium-exposed rats, S100A9 showed a patchy distribution, especially in alveolar epithelial cells and neutrophils (Fig. 6A, B). S100A9 was not clearly expressed in macrophages (Fig. 6B). Image analysis revealed that both In_2_O_3_ and ITO significantly increased the intensity of S100A9 expression compared to the control (*p* < 0.001) (Fig. 6C).

**Fig. 7:**
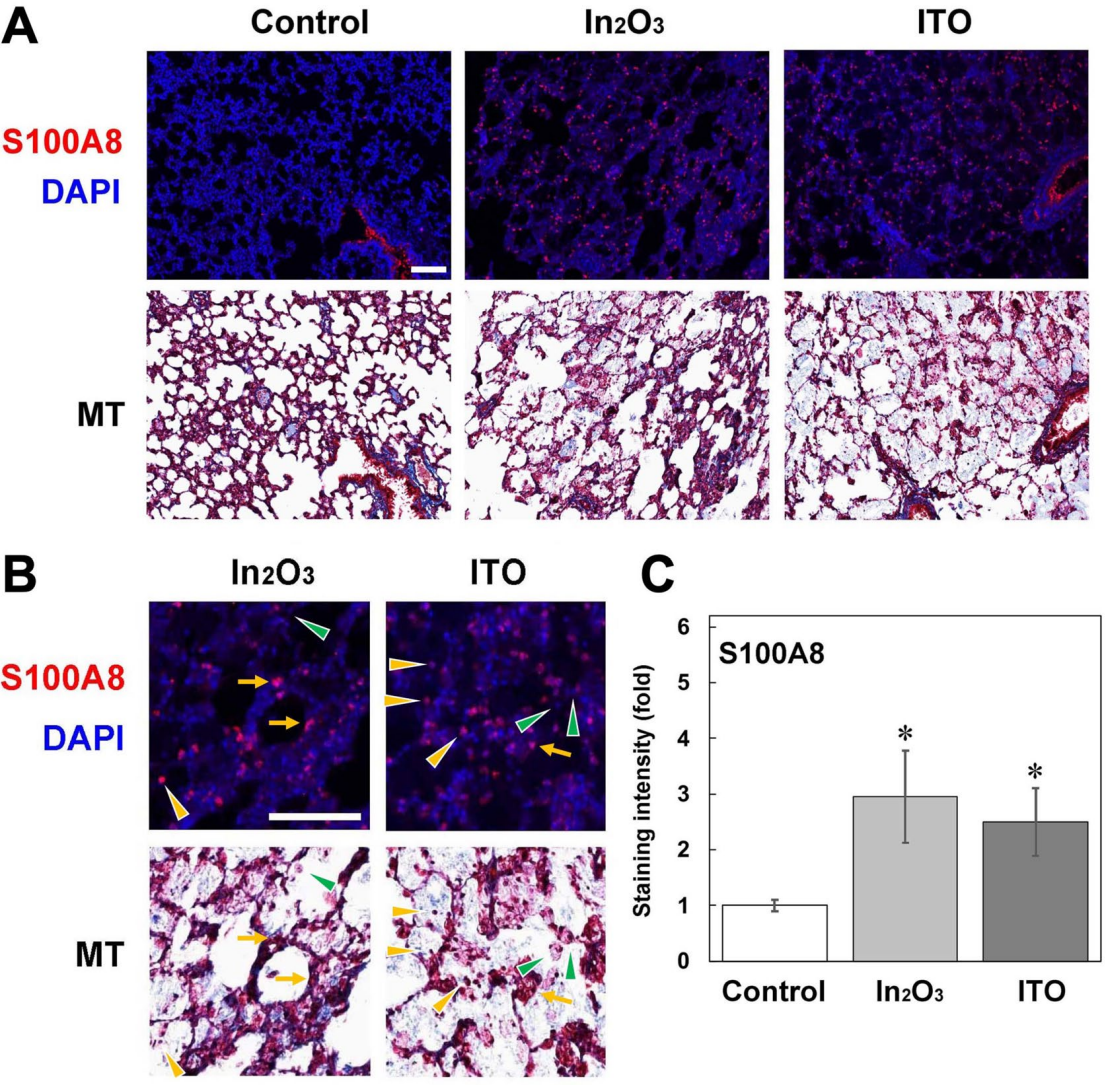
Fluorescent immunohistochemical analysis for S100A8 in lung tissues of indium-exposed rats. **A** Fluorescent images for S100A8 in lung tissues of indium-exposed rats at 12 weeks. Fluorescent immunohistochemistry was performed as described in Materials and methods. The nuclei were stained with DAPI. Histopathological changes were examined by Masson’s trichrome (MT) staining. Scale bar = 100 µm. **B** Enlarged images of fluorescent immunohistochemical analysis for S100A8. *Yellow arrows*, alveolar epithelial cells; *yellow arrowheads*, neutrophils; *green arrowheads*, macrophages. Scale bar = 100 µm. **C** Quantitative image analysis for expression of S100A8. The staining intensity per field was analyzed with an ImageJ software as described in Materials and methods. Data were expressed as means ± SD of 3 independent experiments. *p < 0.05, compared with control by ANOVA followed by Tukey’s test

Figure 7 shows the distribution of S100A8 expression in lung tissues of indium-exposed rats. No or weak S100A8 expression was observed in lung tissues of control rats, whereas S100A8 was expressed in lung tissues of In_2_O_3_- and ITO-exposed rats (Fig. 7A). In a similar manner to S100A9, S100A8 expression was observed in alveolar epithelial cells and neutrophils in indium-exposed rats, but S100A8 was not clearly expressed in macrophages (Fig. 7A, B). Image analysis revealed that both In_2_O_3_ and ITO significantly increased the intensity of S100A8 expression compared to the control (*p* < 0.05) (Fig. 7C).

## Discussion

In this study, we performed microarray analysis for gene expression in the lung tissues of indium-exposed rats to explore genes that contribute to indium-induced respiratory diseases. In In_2_O_3_- and ITO-exposed rats, 233 and 676 genes were significantly upregulated in lung tissues, respectively. Among largely upregulated genes, bioinformatic analysis revealed that *Lcn2*, *S100a9* and *S100a8* shared common gene enrichment including immune and inflammatory responses, response to metal ion and neutrophil migration. These molecules are known to exert inflammatory responses and participate in various pulmonary diseases (Guardado et al. 2021; Sattar et al. 2021). From our analyses and previous literatures, we focused on *Lcn2*, *S100a9* and *S100a8* as potential genes that may play a key role in indium-induced lung diseases.

In further experiments, we validated expression levels of these genes at the transcriptional and translational levels. Real-time PCR revealed that the expression level of *Lcn2* and S100 proteins were significantly increased by indium exposure. However, in Western blotting, LCN2 expression level was only slightly increased by indium compounds, whereas S100A9 was greatly and significantly upregulated. Although the reason for the discrepancy in LCN2 expression at the transcriptional and translational levels is unclear, it is speculated that mRNA translation and/or protein synthesis were interfered. In our experiments, indium exposure induced marked increase in S100A9 and S100A8 expression at the transcriptional and translational levels. In real-time PCR, the expression levels of these genes were strongly correlated, suggesting that these S100 proteins were expressed in parallel and form a heterodimer to exert biological effects, as predicted by PPI network analysis.

S100 proteins constitute a family of calcium-binding proteins including over 20 members in humans. S100 proteins are multifunctional proteins involved in various intracellular and extracellular processes, including proliferation, differentiation, inflammation, tissue repair and migration (Sattar et al. 2021). These proteins are expressed mainly in epithelial cells and granulocytes, and released from activated or damaged cells mediating a wide variety of physiological and pathological responses (Kotsiou et al. 2021). The expression of S100A9 and S100A8 proteins is increased in various pulmonary diseases, including idiopathic pulmonary fibrosis, COPD, COVID-19 and lung cancer (Sattar et al. 2021). Serum S100A8/S100A9 levels were increased in patients with idiopathic pulmonary fibrosis and acute exacerbation of COPD compared with normal subjects, and negatively associated with pulmonary function (Huang et al. 2020; Tanaka et al. 2021). Serum S100A8/A9 levels were also higher in COVID-19 patients than those in healthy controls and associated with the severity of COVID-19 (Shi et al. 2021). Following activation of neutrophils, S100 proteins are secreted or released and function in an autocrine and paracrine manner (Sattar et al. 2021). S100A9 and S100A8 are potent stimulators of neutrophils, which mediate neutrophil migration to inflammatory sites (Ryckman et al. 2003). In this study, HE and Masson’s trichrome staining revealed that indium compounds caused infiltration of neutrophils into alveolar spaces, hyperplasia of alveolar epithelial cells and fibrotic changes. In immunohistochemistry, S100A9 and S100A8 were expressed in alveolar epithelial cells and neutrophils. These findings suggest that these S100 proteins contribute to inflammatory responses and resulting lung injury induced by indium compounds. Although the carcinogenicity of indium compounds to humans is still unclear, a few lung cancer cases have been found in indium-exposed workers (Nakano et al. 2019). Inhalation exposure to indium compounds causes lung tumors in rats (Nagano et al. 2011; National Toxicology Program 2001). The expression rates of S100A9 and S100A8 were significantly higher in lung cancer tissues than in para-cancer tissues, and their expression was associated with tumor differentiation degree (Huang et al. 2018). These findings raise a possibility that S100 proteins contribute to indium-induced carcinogenesis, although carcinogenic changes were not observed under the conditions in this study.

S100 proteins, including S100A9 and S100A8, trigger inflammatory responses through their interactions with toll-like receptor (TLR) 4 and receptor for advanced glycation end products (RAGE) (Vogl et al. 2007). In enrichment analysis of this study, these S100 proteins share the property of Toll-like receptor cascades (R-RNO-168898), supporting the possibility that TLR activation contributes to the pathogenesis of lung diseases. Following the interaction of S100 proteins with these receptors, mitogen-activated protein kinases are phosphorylated and NF-κB, a transcription factor mediating the production of inflammatory cytokines, is activated (Ghavami et al. 2008). S100A9 promotes proliferation of lung fibroblasts and expression of proinflammatory cytokines and collagen type III, via NF-κB signaling (Xu et al. 2013). We have reported that multi-walled carbon nanotube and indium compounds induced nitrative DNA damage in lung epithelial cells through the release of HMGB1 and DNA, which are known as damage-associated molecular patterns (DAMPs) and interact with RAGE, leading to activation of TLR9 (Ahmed et al. 2020; Hiraku et al. 2016). This study raises a possibility that S100 proteins act as DAMPs to mediate inflammatory responses and contribute to indium-induced lung diseases as well as HMGB1 and DNA.

Although we have not investigated precise mechanisms of expression of S100 proteins in this study, it has been reported that oxidative stress can induce the release of S100 proteins from several cell types (Sattar et al. 2021). S100A8/A9 was secreted from human neutrophils exposed to silica, titanium dioxide, fullerene, and single-wall carbon nanotubes and this event was dependent on the production of reactive oxygen species (Tardif et al. 2015). In our microarray analysis, some antioxidant enzymes were upregulated in lung tissues of indium-exposed rats (Supplementary Table 1): *Hmox1* (heme oxygenase 1) was upregulated 17.6-fold by In_2_O_3_ and 19.0-fold by ITO; *Sod2* (superoxide dismutase 2) was upregulated 3.1-fold by In_2_O_3_ and 6.9-fold by ITO; *Gpx1* (glutathione peroxidase 1) was upregulated 2.5-fold by ITO. The upregulation of these antioxidative enzymes may be induced as a protective effect against indium-induced oxidative stress, which contributes to S100 protein expression.

Upregulation of S100 proteins in lung tissues has been demonstrated in animals exposed some industrial chemicals. Inhalation exposure of rats to zinc nanoparticles induced a significant increase of S100A9 and S100A8 in bronchoalveolar lavage fluid (Juang et al. 2014). Pharyngeal aspiration of carbon nanotube (Teeguarden et al. 2011) and welding fume (Zeidler-Erdely et al. 2010) increased the expression levels of S100A9 and S100A8 in mouse lung tissues. Cigarette smoke induced S100A9 expression and contributes to loss of lung function, airspace enlargement and altered expression of inflammatory cytokines in mice (Railwah et al. 2020). Therefore, S100 proteins appear to contribute to lung diseases caused by various environmental and industrial chemicals.

In this study, we first demonstrated that the expression of S100A9 and S100A8 proteins was markedly increased in lung tissues of experimental animals exposed to indium compounds. This study proposes new molecular mechanisms of indium-induced lung diseases and raises a possibility that S100 proteins can be potential biomarkers to evaluate the risk of these diseases. Recently, clinical trials to use drugs targeting S100 proteins have focused on some diseases, such as systemic lupus erythematosus, ischemic heart failure and rheumatoid arthritis (Sattar et al. 2021). Therefore, S100 proteins may be therapeutic targets of lung diseases caused by industrial chemicals.

## Supplementary Information

Below is the link to the electronic supplementary material.

Supplementary file1 (DOCX 170 KB)

Supplementary file2 (DOCX 77 KB)

Supplementary file3 (JPG 3985 KB)

## Data Availability

The data that support the findings of this study are available from the corresponding author on reasonable request for purposes of reproducing the results or replicating the procedure.

## Abbreviations

ITO: Indium tin oxide
In_2_O_3_: Indium oxide
IARC: The International Agency for Research on Cancer
LCN2: Lipocalin-2
COPD: Chronic obstructive pulmonary disorder
COVID-19: Coronavirus disease 2019
HE: Hematoxylin and eosin
ANOVA: Analysis of variance
GO: Gene ontology
PPI: Protein–protein interaction
SDS: Sodium dodecyl sulfate
DAPI: 4',6-Diamidino-2-phenylindole
TLR: Toll-like receptor
RAGE: Advanced glycation end products
DAMPs: Damage-associated molecular patterns

## References

[CR1] Afroz T, Hiraku Y, Ma N, Ahmed S, Oikawa S, Kawanishi S, Murata M (2018) Nitrative DNA damage in cultured macrophages exposed to indium oxide. J Occup Health 60(2):148–155. 10.1539/joh.17-0146-OA 29187674 10.1539/joh.17-0146-OA PMC5886882

[CR2] Ahmed S, Kobayashi H, Afroz T, Ma N, Oikawa S, Kawanishi S, Murata M, Hiraku Y (2020) Nitrative DNA damage in lung epithelial cells exposed to indium nanoparticles and indium ions. Sci Rep 10:10741. 10.1038/s41598-020-67488-3 32612147 10.1038/s41598-020-67488-3 PMC7329867

[CR3] Choi S, Won YL, Kim D, Lee MY, Choi YJ, Park JS, Kim HR, Jung JI, Lee SG, Kim EA (2015) Interstitial lung disorders in the indium workers of Korea: an update study for the relationship with biological exposure indices. Am J Ind Med 58(1):61–68. 10.1002/ajim.22402 25345911 10.1002/ajim.22402

[CR4] Chonan T, Taguchi O, Omae K (2007) Interstitial pulmonary disorders in indium-processing workers. Eur Respir J 29(2):317–324. 10.1183/09031936.00020306 17050566 10.1183/09031936.00020306

[CR5] Cummings KJ, Donat WE, Ettensohn DB, Roggli VL, Ingram P, Kreiss K (2010) Pulmonary alveolar proteinosis in workers at an indium processing facility. Am J Respir Crit Care Med 181(5):458–464. 10.1164/rccm.200907-1022CR 20019344 10.1164/rccm.200907-1022CR PMC3159086

[CR6] Cummings KJ, Nakano M, Omae K, Takeuchi K, Chonan T, Xiao YL, Harley RA, Roggli VL, Hebisawa A, Tallaksen RJ, Trapnell BC, Day GA, Saito R, Stanton ML, Suarthana E, Kreiss K (2012) Indium lung disease. Chest 141(6):1512–1521. 10.1378/chest.11-1880 22207675 10.1378/chest.11-1880 PMC3367484

[CR7] Ghavami S, Rashedi I, Dattilo BM, Eshraghi M, Chazin WJ, Hashemi M, Wesselborg S, Kerkhoff C, Los M (2008) S100A8/A9 at low concentration promotes tumor cell growth via RAGE ligation and MAP kinase-dependent pathway. J Leukoc Biol 83(6):1484–1492. 10.1189/jlb.0607397 18339893 10.1189/jlb.0607397 PMC2737328

[CR8] Guardado S, Ojeda-Juarez D, Kaul M, Nordgren TM (2021) Comprehensive review of lipocalin 2-mediated effects in lung inflammation. Am J Physiol Lung Cell Mol Physiol 321(4):L726–L733. 10.1152/ajplung.00080.2021 34468208 10.1152/ajplung.00080.2021 PMC8560400

[CR9] Guo F, Ma N, Horibe Y, Kawanishi S, Murata M, Hiraku Y (2012) Nitrative DNA damage induced by multi-walled carbon nanotube via endocytosis in human lung epithelial cells. Toxicol Appl Pharmacol 260(2):183–192. 10.1016/j.taap.2012.02.010 22373798 10.1016/j.taap.2012.02.010

[CR10] Hiraku Y, Guo F, Ma N, Yamada T, Wang S, Kawanishi S, Murata M (2016) Multi-walled carbon nanotube induces nitrative DNA damage in human lung epithelial cells via HMGB1-RAGE interaction and Toll-like receptor 9 activation. Part Fibre Toxicol 13(1):16. 10.1186/s12989-016-0127-7 27026438 10.1186/s12989-016-0127-7 PMC4812657

[CR11] Hiraku Y, Nishikawa Y, Ma N, Afroz T, Mizobuchi K, Ishiyama R, Matsunaga Y, Ichinose T, Kawanishi S, Murata M (2017) Nitrative DNA damage induced by carbon-black nanoparticles in macrophages and lung epithelial cells. Mutat Res 818:7–16. 10.1016/j.mrgentox.2017.04.002 10.1016/j.mrgentox.2017.04.002 28477879

[CR12] Hiraku Y, Sakai K, Shibata E, Kamijima M, Hisanaga N, Ma N, Kawanishi S, Murata M (2014) Formation of the nitrative DNA lesion 8-nitroguanine is associated with asbestos contents in human lung tissues: a pilot study. J Occup Health 56(3):186–196. 10.1539/joh.13-0231-oa 24598051 10.1539/joh.13-0231-oa

[CR13] Hiraku Y, Watanabe J, Kaneko A, Ichinose T, Murata M (2021) MicroRNA expression in lung tissues of asbestos-exposed mice: Upregulation of miR-21 and downregulation of tumor suppressor genes Pdcd4 and Reck. J Occup Health 63(1):e12282. 10.1002/1348-9585.12282 34679210 10.1002/1348-9585.12282 PMC8535435

[CR14] Homma T, Ueno T, Sekizawa K, Tanaka A, Hirata M (2003) Interstitial pneumonia developed in a worker dealing with particles containing indium-tin oxide. J Occup Health 45(3):137–139. 10.1539/joh.45.137 14646287 10.1539/joh.45.137

[CR15] Huang H, Huang Q, Tang T, Gu L, Du J, Li Z, Lu X, Zhou X (2018) Clinical significance of calcium-binding protein S100A8 and S100A9 expression in non-small cell lung cancer. Thorac Cancer 9(7):800–804. 10.1111/1759-7714.12649 29733516 10.1111/1759-7714.12649 PMC6026607

[CR16] Huang SJ, Ding ZN, Xiang HX, Fu L, Fei J (2020) Association between serum S100A8/S100A9 heterodimer and pulmonary function in patients with acute exacerbation of chronic obstructive pulmonary disease. Lung 198(4):645–652. 10.1007/s00408-020-00376-9 32661658 10.1007/s00408-020-00376-9

[CR17] IARC (2006) Indium phosphide. IARC Monographs on the Evaluation of Carcinogic Risks to Humans, vol 86. Cobalt in Hard Metals and Cobalt Sulfate, Gallium Arsenide, Indium Phosphide and Vanadium Pentoxide, p 197–224 PMC4781610 16906675

[CR18] IARC (2018) Indium tin oxide. IARC Monographs on the Evaluation of Carcinogenic Risks to Humans, vol 118. Welding, Molybdenum Trioxide, and Indium Tin Oxide, p 283–306

[CR19] Juang YM, Lai BH, Chien HJ, Ho M, Cheng TJ, Lai CC (2014) Changes in protein expression in rat bronchoalveolar lavage fluid after exposure to zinc oxide nanoparticles: an iTRAQ proteomic approach. Rapid Commun Mass Spectrom 28(8):974–980. 10.1002/rcm.6866 24623703 10.1002/rcm.6866

[CR20] Kotsiou OS, Papagiannis D, Papadopoulou R, Gourgoulianis KI (2021) Calprotectin in lung diseases. Int J Mol Sci 22(4):1706. 10.3390/ijms22041706 33567747 10.3390/ijms22041706 PMC7915440

[CR21] Mellett L, Khader SA (2022) S100A8/A9 in COVID-19 pathogenesis: impact on clinical outcomes. Cytokine Growth Factor Rev 63:90–97. 10.1016/j.cytogfr.2021.10.004 34728150 10.1016/j.cytogfr.2021.10.004 PMC8520505

[CR22] Nagano K, Nishizawa T, Umeda Y, Kasai T, Noguchi T, Gotoh K, Ikawa N, Eitaki Y, Kawasumi Y, Yamauchi T, Arito H, Fukushima S (2011) Inhalation carcinogenicity and chronic toxicity of indium-tin oxide in rats and mice. J Occup Health 53(3):175–187. 10.1539/joh.10-0057-oa 21471693 10.1539/joh.10-0057-oa

[CR23] Nakano M, Omae K, Tanaka A, Hirata M (2019) Possibility of lung cancer risk in indium-exposed workers: an 11-year multicenter cohort study. J Occup Health 61(3):251–256. 10.1002/1348-9585.12050 30895696 10.1002/1348-9585.12050 PMC6499344

[CR24] Nakano M, Omae K, Uchida K, Michikawa T, Yoshioka N, Hirata M, Tanaka A (2014) Five-year cohort study: emphysematous progression of indium-exposed workers. Chest 146(5):1166–1175. 10.1378/chest.13-2484 24946105 10.1378/chest.13-2484

[CR25] National Toxicology Program (2001) Toxicology and carcinogenesis studies of indium phosphide (CAS No. 22398-90-7) in F344/N rats and B6C3F1 mice (inhalation studies). Natl Toxicol Program Tech Rep Ser(499):7–340. 12087422

[CR26] Omae K, Nakano M, Tanaka A, Hirata M, Hamaguchi T, Chonan T (2011) Indium lung-case reports and epidemiology. Int Arch Occup Environ Health 84(5):471–477. 10.1007/s00420-010-0575-6 20886351 10.1007/s00420-010-0575-6

[CR27] Railwah C, Lora A, Zahid K, Goldenberg H, Campos M, Wyman A, Jundi B, Ploszaj M, Rivas M, Dabo A, Majka SM, Foronjy R, El Gazzar M, Geraghty P (2020) Cigarette smoke induction of S100A9 contributes to chronic obstructive pulmonary disease. Am J Physiol Lung Cell Mol Physiol 319(6):L1021–L1035. 10.1152/ajplung.00207.2020 32964723 10.1152/ajplung.00207.2020 PMC7938777

[CR28] Ryckman C, Vandal K, Rouleau P, Talbot M, Tessier PA (2003) Proinflammatory activities of S100: proteins S100A8, S100A9, and S100A8/A9 induce neutrophil chemotaxis and adhesion. J Immunol 170(6):3233–3242. 10.4049/jimmunol.170.6.3233 12626582 10.4049/jimmunol.170.6.3233

[CR29] Sattar Z, Lora A, Jundi B, Railwah C, Geraghty P (2021) The S100 protein family as players and therapeutic targets in pulmonary diseases. Pulm Med 2021:5488591. 10.1155/2021/5488591 34239729 10.1155/2021/5488591 PMC8214497

[CR30] Shi H, Zuo Y, Yalavarthi S, Gockman K, Zuo M, Madison JA, Blair C, Woodward W, Lezak SP, Lugogo NL, Woods RJ, Lood C, Knight JS, Kanthi Y (2021) Neutrophil calprotectin identifies severe pulmonary disease in COVID-19. J Leukoc Biol 109(1):67–72. 10.1002/JLB.3COVCRA0720-359R 32869342 10.1002/JLB.3COVCRA0720-359R PMC7902293

[CR31] Tanaka A, Hirata M, Homma T, Kiyohara Y (2010) Chronic pulmonary toxicity study of indium-tin oxide and indium oxide following intratracheal instillations into the lungs of hamsters. J Occup Health 52(1):14–22. 10.1539/joh.l9097 19940388 10.1539/joh.l9097

[CR32] Tanaka K, Enomoto N, Hozumi H, Isayama T, Naoi H, Aono Y, Katsumata M, Yasui H, Karayama M, Suzuki Y, Furuhashi K, Fujisawa T, Inui N, Nakamura Y, Suda T (2021) Serum S100A8 and S100A9 as prognostic biomarkers in acute exacerbation of idiopathic pulmonary fibrosis. Respir Investig 59(6):827–836. 10.1016/j.resinv.2021.05.008 34154976 10.1016/j.resinv.2021.05.008

[CR33] Tardif MR, Chapeton-Montes JA, Posvandzic A, Page N, Gilbert C, Tessier PA (2015) Secretion of S100A8, S100A9, and S100A12 by neutrophils involves reactive oxygen species and potassium efflux. J Immunol Res 2015:296149. 10.1155/2015/296149 27057553 10.1155/2015/296149 PMC4736198

[CR34] Teeguarden JG, Webb-Robertson BJ, Waters KM, Murray AR, Kisin ER, Varnum SM, Jacobs JM, Pounds JG, Zanger RC, Shvedova AA (2011) Comparative proteomics and pulmonary toxicity of instilled single-walled carbon nanotubes, crocidolite asbestos, and ultrafine carbon black in mice. Toxicol Sci 120(1):123–135. 10.1093/toxsci/kfq363 21135415 10.1093/toxsci/kfq363 PMC3044201

[CR35] U.S. Geological Survey (2023) Indium. Mineral Commodity Summaries 2023. U.S. Geological Publishing Office, St. Louis, USA, p 88–89

[CR36] Vogl T, Tenbrock K, Ludwig S, Leukert N, Ehrhardt C, van Zoelen MA, Nacken W, Foell D, van der Poll T, Sorg C, Roth J (2007) Mrp8 and Mrp14 are endogenous activators of Toll-like receptor 4, promoting lethal, endotoxin-induced shock. Nature Med 13(9):1042–1049. 10.1038/nm1638 17767165 10.1038/nm1638

[CR37] Xu X, Chen H, Zhu X, Ma Y, Liu Q, Xue Y, Chu H, Wu W, Wang J, Zou H (2013) S100A9 promotes human lung fibroblast cells activation through receptor for advanced glycation end-product-mediated extracellular-regulated kinase 1/2, mitogen-activated protein-kinase and nuclear factor-κB-dependent pathways. Clin Exp Immunol 173(3):523–535. 10.1111/cei.12139 23682982 10.1111/cei.12139 PMC3949640

[CR38] Zeidler-Erdely PC, Kashon ML, Li S, Antonini JM (2010) Response of the mouse lung transcriptome to welding fume: effects of stainless and mild steel fumes on lung gene expression in A/J and C57BL/6J mice. Respir Res 11:70. 10.1186/1465-9921-11-70 20525249 10.1186/1465-9921-11-70 PMC2892448

[CR39] Zhou Y, Zhou B, Pache L, Chang M, Khodabakhshi AH, Tanaseichuk O, Benner C, Chanda SK (2019) Metascape provides a biologist-oriented resource for the analysis of systems-level datasets. Nat Commun 10(1):1523. 10.1038/s41467-019-09234-6 30944313 10.1038/s41467-019-09234-6 PMC6447622

