# Supplementary material for: Microarray analysis of gene expression in lung tissues of indium-exposed rats: possible roles of S100 proteins in lung diseases
Source: Arch Toxicol. 2024 Nov 8;99(1):245–58. doi: 10.1007/s00204-024-03897-x (PMC11742277; doi:10.1007/s00204-024-03897-x)
Supplement: Supplementary file 1 — Supplementary file1 (DOCX 170 KB) [file 204_2024_3897_MOESM1_ESM.docx]

**Table S1** Upregulated genes in indium-exposed rats

**(A) In_2_O_3_-exposed rats**

| Rank | ***GeneSymbol*** | *p* (ANOVA) | fold | Description |
| --- | --- | --- | --- | --- |
| 1 | ***Lcn2*** | 2.4E-06 | 49.35 | Rattus norvegicus lipocalin 2 (Lcn2), mRNA [NM_130741] |
| 2 | ***S100a9*** | 8.5E-08 | 30.23 | Rattus norvegicus S100 calcium binding protein A9 (S100a9), mRNA [NM_053587] |
| 3 | ***Trem2*** | 3.9E-06 | 29.87 | Rattus norvegicus triggering receptor expressed on myeloid cells 2 (Trem2), mRNA [NM_001106884] |
| 4 | ***Hmox1*** | 3.7E-04 | 17.64 | Rattus norvegicus heme oxygenase (decycling) 1 (Hmox1), mRNA [NM_012580] |
| 5 | ***Lilrb4*** | 2.1E-04 | 17.57 | PREDICTED: Rattus norvegicus leukocyte immunoglobulin-like receptor, subfamily B, member 4 (Lilrb4), transcript variant X1, mRNA [XM_006228238] |
| 6 | ***Bag3*** | 6.0E-04 | 17.55 | Rattus norvegicus Bcl2-associated athanogene 3 (Bag3), mRNA [NM_001011936] |
| 7 | ***Bpifb1*** | 4.2E-04 | 16.49 | Rattus norvegicus BPI fold containing family B, member 1 (Bpifb1), mRNA [NM_001077680] |
| 8 | ***Chia*** | 1.2E-05 | 16.40 | Rattus norvegicus chitinase, acidic (Chia), mRNA [NM_207586] |
| 9 | ***Zfand2a*** | 2.3E-04 | 14.73 | Rattus norvegicus zinc finger, AN1-type domain 2A (Zfand2a), mRNA [NM_001008363] |
| 10 | ***S100a8*** | 1.2E-05 | 11.45 | Rattus norvegicus S100 calcium binding protein A8 (S100a8), mRNA [NM_053822] |
| 11 | ***Cxcl1*** | 8.3E-04 | 9.91 | Rattus norvegicus chemokine (C-X-C motif) ligand 1 (Cxcl1), mRNA [NM_030845] |
| 12 | ***Ly6i*** | 2.9E-05 | 9.83 | Rattus norvegicus lymphocyte antigen 6 complex, locus I (Ly6i), mRNA [NM_139257] |
| 13 | ***LOC689489*** | 2.6E-04 | 9.81 | Protein LOC689489 [Source:UniProtKB/TrEMBL;Acc:D4AAZ5] [ENSRNOT00000035746] |
| 14 | ***Hsp90aa1*** | 1.7E-04 | 9.23 | Rattus norvegicus heat shock protein 90, alpha (cytosolic), class A member 1 (Hsp90aa1), mRNA [NM_175761] |
| 15 | ***C3*** | 1.8E-04 | 8.77 | Rattus norvegicus complement component 3 (C3), mRNA [NM_016994] |
| 16 | ***Uspl1*** | 8.9E-03 | 8.76 | Rattus norvegicus ubiquitin specific peptidase like 1 (Uspl1), transcript variant 1, mRNA [NM_001198555] |
| 17 | ***Chordc1*** | 4.2E-05 | 8.47 | Rattus norvegicus cysteine and histidine-rich domain (CHORD)-containing 1 (Chordc1), mRNA [NM_001108128] |
| 18 | ***Dnaja1*** | 4.9E-05 | 8.25 | Rattus norvegicus DnaJ (Hsp40) homolog, subfamily A, member 1 (Dnaja1), mRNA [NM_022934] |
| 19 | ***Tf*** | 5.0E-05 | 8.01 | Rattus norvegicus transferrin (Tf), mRNA [NM_001013110] |
| 20 | ***Hsp90aa1*** | 1.5E-04 | 7.90 | Rattus norvegicus heat shock protein 90, alpha (cytosolic), class A member 1 (Hsp90aa1), mRNA [NM_175761] |
| 21 | ***Ahsa2*** | 1.6E-04 | 7.76 | Rattus norvegicus AHA1, activator of heat shock protein ATPase 2 (Ahsa2), mRNA [NM_001107241] |
| 22 | ***Cfb*** | 6.6E-05 | 7.67 | Rattus norvegicus complement factor B (Cfb), mRNA [NM_212466] |
| 23 | ***Cacybp*** | 1.1E-04 | 7.43 | Rattus norvegicus calcyclin binding protein (Cacybp), mRNA [NM_001004208] |
| 24 | ***Plod1*** | 1.7E-02 | 7.29 | Rattus norvegicus procollagen-lysine, 2-oxoglutarate 5-dioxygenase 1 (Plod1), mRNA [NM_053827] |
| 25 | ***Ly6i*** | 8.4E-05 | 7.27 | lymphocyte antigen 6 complex, locus I (Ly6i), mRNA [Source:RefSeq mRNA;Acc:NM_139257] [ENSRNOT00000009395] |
| 26 | ***Hsp90aa1*** | 3.8E-05 | 7.26 | Rattus norvegicus heat shock protein 90, alpha (cytosolic), class A member 1 (Hsp90aa1), mRNA [NM_175761] |
| 27 | ***Fkbp4*** | 6.4E-04 | 7.16 | Rattus norvegicus FK506 binding protein 4 (Fkbp4), mRNA [NM_001191863] |
| 28 | ***Cfd*** | 2.5E-04 | 6.95 | Rattus norvegicus complement factor D (adipsin) (Cfd), mRNA [NM_001077642] |
| 29 | ***C4a*** | 1.2E-04 | 6.80 | Rattus norvegicus complement component 4A (Rodgers blood group) (C4a), mRNA [NM_031504] |
| 30 | ***Hspb1*** | 2.2E-03 | 6.77 | Rattus norvegicus heat shock protein 1 (Hspb1), mRNA [NM_031970] |
| 31 | ***Cd68*** | 5.2E-05 | 6.62 | Rattus norvegicus Cd68 molecule (Cd68), mRNA [NM_001031638] |
| 32 | ***Hspa1b*** | 3.2E-03 | 6.57 | Rattus norvegicus heat shock 70kD protein 1B (mapped) (Hspa1b), mRNA [NM_212504] |
| 33 | ***Qsox1*** | 1.2E-04 | 6.44 | Rattus norvegicus quiescin Q6 sulfhydryl oxidase 1 (Qsox1), transcript variant 2, mRNA [NM_053431] |
| 34 | ***Gpnmb*** | 1.2E-05 | 6.40 | Rattus norvegicus glycoprotein (transmembrane) nmb (Gpnmb), mRNA [NM_133298] |
| 35 | ***Hspe1*** | 5.4E-04 | 6.38 | Rattus norvegicus heat shock protein 1 (chaperonin 10) (Hspe1), mRNA [NM_012966] |
| 36 | ***Hspe1*** | 6.4E-04 | 6.16 | Rattus norvegicus heat shock protein 1 (chaperonin 10) (Hspe1), mRNA [NM_012966] |
| 37 | ***Hspa5*** | 5.5E-04 | 5.71 | Rattus norvegicus heat shock protein 5 (Hspa5), mRNA [NM_013083] |
| 38 | ***Creg1*** | 3.9E-03 | 5.62 | Rattus norvegicus cellular repressor of E1A-stimulated genes 1 (Creg1), mRNA [NM_001105966] |
| 39 | ***Dedd2*** | 2.8E-02 | 5.62 | PREDICTED: Rattus norvegicus death effector domain containing 2 (Dedd2), transcript variant 1, mRNA [XM_003748789] |
| 40 | ***Tmem173*** | 2.3E-05 | 5.55 | Rattus norvegicus transmembrane protein 173 (Tmem173), mRNA [NM_001109122] |
| 41 | ***Sdf2l1*** | 4.1E-05 | 5.46 | Rattus norvegicus stromal cell-derived factor 2-like 1 (Sdf2l1), mRNA [NM_001109433] |
| 42 | ***Ifi30*** | 1.2E-04 | 5.38 | Rattus norvegicus interferon gamma inducible protein 30 (Ifi30), mRNA [NM_001030026] |
| 43 | ***Ccl9*** | 5.4E-03 | 5.28 | Rattus norvegicus chemokine (C-C motif) ligand 9 (Ccl9), mRNA [NM_001012357] |
| 44 | ***Pnpla7*** | 6.3E-03 | 5.17 | Rattus norvegicus patatin-like phospholipase domain containing 7 (Pnpla7), mRNA [NM_144738] |
| 45 | ***Hspd1*** | 1.6E-03 | 5.16 | Rattus norvegicus heat shock protein 1 (chaperonin) (Hspd1), mRNA [NM_022229] |
| 46 | ***Il1b*** | 2.4E-03 | 5.02 | Rattus norvegicus interleukin 1 beta (Il1b), mRNA [NM_031512] |
| 47 | ***Hp*** | 5.4E-04 | 4.83 | Rattus norvegicus haptoglobin (Hp), mRNA [NM_012582] |
| 48 | ***Hspa8*** | 4.5E-06 | 4.76 | Rattus norvegicus heat shock 70kDa protein 8 (Hspa8), mRNA [NM_024351] |
| 49 | ***Dnaja4*** | 2.1E-02 | 4.70 | Rattus norvegicus DnaJ (Hsp40) homolog, subfamily A, member 4 (Dnaja4), mRNA [NM_001025411] |
| 50 | ***Scimp*** | 3.8E-04 | 4.68 | PREDICTED: Rattus norvegicus SLP adaptor and CSK interacting membrane protein (Scimp), mRNA [XM_003752341] |
| 51 | ***RT1-A2*** | 3.3E-02 | 4.66 | Rattus norvegicus RT1 class Ia, locus A2 (RT1-A2), mRNA [NM_001008829] |
| 52 | ***LOC688932*** | 3.2E-04 | 4.61 | PREDICTED: Rattus norvegicus heat shock cognate 71 kDa protein-like (LOC688932), misc_RNA [XR_085946] |
| 53 | ***RT1-CE2*** | 5.5E-04 | 4.57 | Rattus norvegicus RT1 class I, locus CE2 (RT1-CE2), mRNA [NM_001008840] |
| 54 | ***LOC680491*** | 7.0E-04 | 4.51 | PREDICTED: Rattus norvegicus heat shock protein HSP 90-alpha-like (LOC680491), misc_RNA [XR_146567] |
| 55 | ***Fetub*** | 2.0E-03 | 4.45 | Rattus norvegicus fetuin B (Fetub), mRNA [NM_053348] |
| 56 | ***Hspa8*** | 1.1E-06 | 4.42 | Rattus norvegicus heat shock 70kDa protein 8 (Hspa8), mRNA [NM_024351] |
| 57 | ***Hsp90ab1*** | 1.3E-03 | 4.38 | Rattus norvegicus heat shock protein 90 alpha (cytosolic), class B member 1 (Hsp90ab1), mRNA [NM_001004082] |
| 58 | ***Khk*** | 4.4E-04 | 4.36 | Rattus norvegicus ketohexokinase (Khk), mRNA [NM_031855] |
| 59 | ***Fcgr2b*** | 3.4E-04 | 4.35 | Rattus norvegicus Fc fragment of IgG, low affinity IIb, receptor (CD32) (Fcgr2b), mRNA [NM_175756] |
| 60 | ***Mt1a*** | 2.2E-04 | 4.33 | Rattus norvegicus metallothionein 1a (Mt1a), mRNA [NM_138826] |
| 61 | ***C1qa*** | 3.3E-04 | 4.33 | Rattus norvegicus complement component 1, q subcomponent, A chain (C1qa), mRNA [NM_001008515] |
| 62 | ***Hspa8*** | 2.0E-04 | 4.29 | Rattus norvegicus heat shock 70kDa protein 8 (Hspa8), mRNA [NM_024351] |
| 63 | ***Nupr1*** | 9.5E-04 | 4.27 | Rattus norvegicus nuclear protein, transcriptional regulator, 1 (Nupr1), mRNA [NM_053611] |
| 64 | ***Hsp90ab1*** | 1.2E-03 | 4.22 | heat shock protein 90 alpha (cytosolic), class B member 1 (Hsp90ab1), mRNA [Source:RefSeq mRNA;Acc:NM_001004082] [ENSRNOT00000026920] |
| 65 | ***Cx3cl1*** | 1.6E-03 | 4.20 | Rattus norvegicus chemokine (C-X3-C motif) ligand 1 (Cx3cl1), mRNA [NM_134455] |
| 66 | ***Hspa8*** | 3.2E-04 | 4.18 | Rattus norvegicus heat shock 70kDa protein 8 (Hspa8), mRNA [NM_024351] |
| 67 | ***Hsp90ab1*** | 6.4E-04 | 4.17 | Rattus norvegicus heat shock protein 90 alpha (cytosolic), class B member 1 (Hsp90ab1), mRNA [NM_001004082] |
| 68 | ***Ctsb*** | 2.8E-04 | 4.09 | Rattus norvegicus cathepsin B (Ctsb), mRNA [NM_022597] |
| 69 | ***Bcl3*** | 3.2E-03 | 4.08 | Rattus norvegicus B-cell CLL/lymphoma 3 (Bcl3), mRNA [NM_001109422] |
| 70 | ***Dnajb1*** | 9.2E-03 | 4.08 | Rattus norvegicus DnaJ (Hsp40) homolog, subfamily B, member 1 (Dnajb1), mRNA [NM_001108441] |
| 71 | ***Ahsa1*** | 1.5E-04 | 4.08 | Rattus norvegicus AHA1, activator of heat shock 90kDa protein ATPase homolog 1 (yeast) (Ahsa1), mRNA [NM_001115034] |
| 72 | ***B4galt5*** | 6.8E-03 | 4.06 | Rattus norvegicus UDP-Gal:betaGlcNAc beta 1,4-galactosyltransferase, polypeptide 5 (B4galt5), mRNA [NM_001108608] |
| 73 | ***Endod1*** | 2.2E-03 | 4.05 | PREDICTED: Rattus norvegicus endonuclease domain containing 1 (Endod1), mRNA [XM_002729871] |
| 74 | ***Smpdl3a*** | 2.4E-04 | 4.02 | Rattus norvegicus sphingomyelin phosphodiesterase, acid-like 3A (Smpdl3a), mRNA [NM_001005539] |
| 75 | ***Tjp3*** | 1.8E-03 | 4.01 | Rattus norvegicus tight junction protein 3 (Tjp3), mRNA [NM_001108073] |
| 76 | ***LOC100909504*** | 5.2E-05 | 3.99 | PREDICTED: Rattus norvegicus heat shock protein HSP 90-beta-like (LOC100909504), partial mRNA [XM_003752259] |
| 77 | ***Rasa4*** | 1.4E-03 | 3.97 | PREDICTED: Rattus norvegicus RAS p21 protein activator 4 (Rasa4), transcript variant 2, mRNA [XM_002724809] |
| 78 | ***P4ha2*** | 2.3E-03 | 3.79 | Rattus norvegicus prolyl 4-hydroxylase, alpha polypeptide II (P4ha2), mRNA [NM_001108275] |
| 79 | ***Serpinb1a*** | 2.3E-04 | 3.78 | Rattus norvegicus serine (or cysteine) proteinase inhibitor, clade B, member 1a (Serpinb1a), mRNA [NM_001031642] |
| 80 | ***Plat*** | 4.2E-03 | 3.77 | Rattus norvegicus plasminogen activator, tissue (Plat), mRNA [NM_013151] |
| 81 | ***Serpinb1a*** | 1.8E-04 | 3.76 | Rattus norvegicus serine (or cysteine) proteinase inhibitor, clade B, member 1a (Serpinb1a), mRNA [NM_001031642] |
| 82 | ***Cyba*** | 6.9E-04 | 3.75 | Rattus norvegicus cytochrome b-245, alpha polypeptide (Cyba), mRNA [NM_024160] |
| 83 | ***Pld1*** | 6.6E-04 | 3.72 | Rattus norvegicus phospholipase D1 (Pld1), mRNA [NM_030992] |
| 84 | ***Atp1b1*** | 2.9E-03 | 3.71 | Rattus norvegicus ATPase, Na+/K+ transporting, beta 1 polypeptide (Atp1b1), mRNA [NM_013113] |
| 85 | ***Hsp90b1*** | 7.1E-03 | 3.70 | Rattus norvegicus heat shock protein 90, beta, member 1 (Hsp90b1), mRNA [NM_001012197] |
| 86 | ***Lrg1*** | 9.5E-04 | 3.68 | Rattus norvegicus leucine-rich alpha-2-glycoprotein 1 (Lrg1), mRNA [NM_001009717] |
| 87 | ***LOC100909505*** | 1.4E-03 | 3.67 | PREDICTED: Rattus norvegicus N-acetylglucosamine-6-sulfatase-like (LOC100909505), mRNA [XM_003750325] |
| 88 | ***Serping1*** | 1.4E-03 | 3.66 | Rattus norvegicus serpin peptidase inhibitor, clade G (C1 inhibitor), member 1 (Serping1), mRNA [NM_199093] |
| 89 | ***Serpinh1*** | 1.3E-03 | 3.66 | Rattus norvegicus serpin peptidase inhibitor, clade H (heat shock protein 47), member 1, (collagen binding protein 1) (Serpinh1), mRNA [NM_017173] |
| 90 | ***Hyou1*** | 1.4E-02 | 3.65 | Rattus norvegicus hypoxia up-regulated 1 (Hyou1), transcript variant 1, mRNA [NM_138867] |
| 91 | ***Calr*** | 5.2E-03 | 3.63 | Rattus norvegicus calreticulin (Calr), mRNA [NM_022399] |
| 92 | ***Ddit3*** | 2.1E-03 | 3.63 | Rattus norvegicus DNA-damage inducible transcript 3 (Ddit3), transcript variant 1, mRNA [NM_001109986] |
| 93 | ***Ncf4*** | 6.7E-04 | 3.59 | Rattus norvegicus neutrophil cytosolic factor 4 (Ncf4), mRNA [NM_001127304] |
| 94 | ***Ifi27l2b*** | 7.6E-03 | 3.58 | Rattus norvegicus interferon, alpha-inducible protein 27 like 2B (Ifi27l2b), mRNA [NM_206846] |
| 95 | ***Prkar1b*** | 2.8E-04 | 3.55 | Rattus norvegicus protein kinase, cAMP dependent regulatory, type I, beta (Prkar1b), mRNA [NM_001033679] |
| 96 | ***Tec*** | 3.9E-03 | 3.55 | Rattus norvegicus tec protein tyrosine kinase (Tec), mRNA [NM_053432] |
| 97 | ***Mt2A*** | 1.9E-03 | 3.52 | Rattus norvegicus metallothionein 2A (Mt2A), mRNA [NM_001137564] |
| 98 | ***Igsf8*** | 1.1E-04 | 3.49 | Rattus norvegicus immunoglobulin superfamily, member 8 (Igsf8), mRNA [NM_001014787] |
| 99 | ***LOC291871*** | 8.5E-05 | 3.47 | PREDICTED: Rattus norvegicus heat shock cognate protein HSP 90-beta-like (LOC291871), mRNA [XM_006255432] |
| 100 | ***Fam105a*** | 1.7E-03 | 3.45 | Rattus norvegicus family with sequence similarity 105, member A (Fam105a), mRNA [NM_001037648] |
| 101 | ***Cd6*** | 1.2E-03 | 3.44 | Rattus norvegicus Cd6 molecule (Cd6), mRNA [NM_175577] |
| 102 | ***Lgals3bp*** | 2.7E-03 | 3.43 | Rattus norvegicus lectin, galactoside-binding, soluble, 3 binding protein (Lgals3bp), mRNA [NM_139096] |
| 103 | ***Rnaset2*** | 2.1E-04 | 3.40 | Rattus norvegicus ribonuclease T2 (Rnaset2), mRNA [NM_001106210] |
| 104 | ***Litaf*** | 3.0E-04 | 3.33 | Rattus norvegicus lipopolysaccharide-induced TNF factor (Litaf), mRNA [NM_001105735] |
| 105 | ***Rnaset2*** | 1.7E-04 | 3.33 | Rattus norvegicus ribonuclease T2 (Rnaset2), mRNA [NM_001106210] |
| 106 | ***Pla2g1b*** | 1.0E-02 | 3.33 | Rattus norvegicus phospholipase A2, group IB, pancreas (Pla2g1b), mRNA [NM_031585] |
| 107 | ***Fam46a*** | 3.0E-04 | 3.32 | Rattus norvegicus family with sequence similarity 46, member A (Fam46a), mRNA [NM_001106844] |
| 108 | ***Sftpa1*** | 2.7E-03 | 3.31 | Rattus norvegicus surfactant protein A1 (Sftpa1), transcript variant 2, mRNA [NM_017329] |
| 109 | ***Ctsa*** | 5.5E-04 | 3.30 | Rattus norvegicus cathepsin A (Ctsa), mRNA [NM_001011959] |
| 110 | ***Herpud1*** | 1.3E-02 | 3.27 | Rattus norvegicus homocysteine-inducible, endoplasmic reticulum stress-inducible, ubiquitin-like domain member 1 (Herpud1), mRNA [NM_053523] |
| 111 | ***Tbpl1*** | 4.2E-02 | 3.25 | Rattus norvegicus TBP-like 1 (Tbpl1), mRNA [NM_001127201] |
| 112 | ***Gngt2*** | 2.3E-04 | 3.23 | Rattus norvegicus guanine nucleotide binding protein (G protein), gamma transducing activity polypeptide 2 (Gngt2), mRNA [NM_001135767] |
| 113 | ***Lgals3*** | 8.1E-04 | 3.20 | Rattus norvegicus lectin, galactoside-binding, soluble, 3 (Lgals3), mRNA [NM_031832] |
| 114 | ***RGD1563378*** | 8.7E-04 | 3.20 | PREDICTED: Rattus norvegicus ferritin heavy polypeptide-like 17-like (RGD1563378), transcript variant X1, mRNA [XM_006227337] |
| 115 | ***Grn*** | 6.3E-04 | 3.19 | Rattus norvegicus granulin (Grn), transcript variant 1, mRNA [NM_017113] |
| 116 | ***LOC100360087*** | 4.7E-05 | 3.18 | PREDICTED: Rattus norvegicus ferritin light chain 1-like (LOC100360087), mRNA [XM_002726637] |
| 117 | ***Cd83*** | 2.5E-03 | 3.15 | Rattus norvegicus CD83 molecule (Cd83), mRNA [NM_001108410] |
| 118 | ***Fcgr2b*** | 5.4E-04 | 3.13 | Rattus norvegicus Fc fragment of IgG, low affinity IIb, receptor (CD32) (Fcgr2b), mRNA [NM_175756] |
| 119 | ***Npc2*** | 2.8E-04 | 3.13 | Rattus norvegicus Niemann-Pick disease, type C2 (Npc2), mRNA [NM_173118] |
| 120 | ***Sod2*** | 3.4E-04 | 3.12 | Rattus norvegicus superoxide dismutase 2, mitochondrial (Sod2), mRNA [NM_017051] |
| 121 | ***Sbno2*** | 2.2E-03 | 3.12 | Rattus norvegicus strawberry notch homolog 2 (Drosophila) (Sbno2), mRNA [NM_001108068] |
| 122 | ***Fxyd5*** | 5.3E-04 | 3.10 | Rattus norvegicus FXYD domain-containing ion transport regulator 5 (Fxyd5), transcript variant 1, mRNA [NM_021909] |
| 123 | ***Ctsh*** | 5.6E-04 | 3.10 | Rattus norvegicus cathepsin H (Ctsh), mRNA [NM_012939] |
| 124 | ***RT1-EC2*** | 1.5E-02 | 3.10 | Rattus norvegicus RT1 class Ib, locus EC2 (RT1-EC2), mRNA [NM_012645] |
| 125 | ***Ftl1*** | 5.4E-05 | 3.10 | Rattus norvegicus ferritin light chain 1 (Ftl1), mRNA [NM_022500] |
| 126 | ***Manf*** | 7.0E-05 | 3.09 | Rattus norvegicus mesencephalic astrocyte-derived neurotrophic factor (Manf), mRNA [NM_001108183] |
| 127 | ***Lipa*** | 9.7E-04 | 3.08 | Rattus norvegicus lipase A, lysosomal acid, cholesterol esterase (Lipa), mRNA [NM_012732] |
| 128 | ***Jak3*** | 4.0E-02 | 3.07 | Rattus norvegicus Janus kinase 3 (Jak3), mRNA [NM_012855] |
| 129 | ***Azi2*** | 1.2E-02 | 3.07 | Rattus norvegicus 5-azacytidine induced 2 (Azi2), mRNA [NM_001025705] |
| 130 | ***C1qb*** | 3.5E-04 | 3.06 | Rattus norvegicus complement component 1, q subcomponent, B chain (C1qb), mRNA [NM_019262] |
| 131 | ***Trmt1l*** | 3.4E-02 | 3.05 | Rattus norvegicus tRNA methyltransferase 1-like (Trmt1l), mRNA [NM_001037192] |
| 132 | ***Tmem33*** | 1.4E-03 | 3.04 | Rattus norvegicus transmembrane protein 33 (Tmem33), transcript variant 1, mRNA [NM_021671] |
| 133 | ***Ctsd*** | 2.2E-03 | 3.04 | Rattus norvegicus cathepsin D (Ctsd), mRNA [NM_134334] |
| 134 | ***Tnnt2*** | 1.4E-03 | 3.04 | Rattus norvegicus troponin T type 2 (cardiac) (Tnnt2), mRNA [NM_012676] |
| 135 | ***Fabp5*** | 1.2E-04 | 3.03 | Rattus norvegicus fatty acid binding protein 5, epidermal (Fabp5), mRNA [NM_145878] |
| 136 | ***Cxcl3*** | 5.1E-04 | 3.03 | PREDICTED: Rattus norvegicus chemokine (C-X-C motif) ligand 3 (Cxcl3), transcript variant X1, mRNA [XM_006250721] |
| 137 | ***B3gnt7*** | 2.1E-04 | 3.01 | Rattus norvegicus UDP-GlcNAc:betaGal beta-1,3-N-acetylglucosaminyltransferase 7 (B3gnt7), mRNA [NM_001012134] |
| 138 | ***C2*** | 5.2E-03 | 3.00 | Rattus norvegicus complement component 2 (C2), mRNA [NM_172222] |
| 139 | ***Fam65b*** | 1.4E-04 | 2.99 | Rattus norvegicus family with sequence similarity 65, member B (Fam65b), mRNA [NM_001014009] |
| 140 | ***Rt1.aa*** | 6.5E-03 | 2.99 | Rattus norvegicus MHC class I RT1.Aa alpha-chain (Rt1.aa), mRNA [NM_001134701] |
| 141 | ***Cxcl3*** | 6.3E-03 | 2.97 | Rattus norvegicus chemokine (C-X-C motif) ligand 3 (Cxcl3), mRNA [NM_138522] |
| 142 | ***Ctss*** | 2.2E-04 | 2.97 | Rattus norvegicus cathepsin S (Ctss), mRNA [NM_017320] |
| 143 | ***Myo5a*** | 2.2E-03 | 2.96 | Rattus norvegicus myosin VA (Myo5a), mRNA [NM_022178] |
| 144 | ***Man2b2*** | 6.5E-03 | 2.94 | Rattus norvegicus mannosidase, alpha, class 2B, member 2 (Man2b2), mRNA [NM_001134971] |
| 145 | ***Trpv2*** | 6.1E-04 | 2.93 | Rattus norvegicus transient receptor potential cation channel, subfamily V, member 2 (Trpv2), transcript variant 1, mRNA [NM_017207] |
| 146 | ***Ubqln1*** | 5.7E-03 | 2.92 | Rattus norvegicus ubiquilin 1 (Ubqln1), mRNA [NM_053747] |
| 147 | ***RGD1561520*** | 8.9E-05 | 2.91 | PREDICTED: Rattus norvegicus ferritin light chain 1-like (RGD1561520), mRNA [XM_345159] |
| 148 | ***Lgmn*** | 7.6E-04 | 2.89 | Rattus norvegicus legumain (Lgmn), mRNA [NM_022226] |
| 149 | ***Sftpd*** | 2.4E-03 | 2.88 | Rattus norvegicus surfactant protein D (Sftpd), mRNA [NM_012878] |
| 150 | ***Grin2c*** | 2.1E-04 | 2.87 | Rattus norvegicus glutamate receptor, ionotropic, N-methyl D-aspartate 2C (Grin2c), mRNA [NM_012575] |
| 151 | ***Lamp1*** | 6.9E-04 | 2.86 | Rattus norvegicus lysosomal-associated membrane protein 1 (Lamp1), mRNA [NM_012857] |
| 152 | ***Ttyh2*** | 8.2E-04 | 2.86 | PREDICTED: Rattus norvegicus tweety family member 2 (Ttyh2), mRNA [XM_006220891] |
| 153 | ***Elf3*** | 3.3E-03 | 2.85 | Rattus norvegicus E74-like factor 3 (Elf3), mRNA [NM_001024768] |
| 154 | ***Atp6v1b2*** | 2.5E-04 | 2.85 | Rattus norvegicus ATPase, H transporting, lysosomal V1 subunit B2 (Atp6v1b2), mRNA [NM_057213] |
| 155 | ***Cct7*** | 1.1E-02 | 2.83 | Rattus norvegicus chaperonin containing Tcp1, subunit 7 (eta) (Cct7), mRNA [NM_001106603] |
| 156 | ***Ccdc93*** | 2.8E-03 | 2.83 | Rattus norvegicus coiled-coil domain containing 93 (Ccdc93), mRNA [NM_001024997] |
| 157 | ***Ly6c*** | 7.8E-04 | 2.80 | Rattus norvegicus Ly6-C antigen (Ly6c), mRNA [NM_020103] |
| 158 | ***Slc5a3*** | 5.4E-03 | 2.80 | Rattus norvegicus solute carrier family 5 (sodium/myo-inositol cotransporter), member 3 (Slc5a3), mRNA [NM_053715] |
| 159 | ***Cd63*** | 6.9E-04 | 2.79 | Rattus norvegicus Cd63 molecule (Cd63), mRNA [NM_017125] |
| 160 | ***LOC678766*** | 1.0E-02 | 2.78 | PREDICTED: Rattus norvegicus rho-related GTP-binding protein RhoU-like (LOC678766), mRNA [XM_006222804] |
| 161 | ***Cd53*** | 6.9E-04 | 2.77 | Rattus norvegicus Cd53 molecule (Cd53), mRNA [NM_012523] |
| 162 | ***Ubtd1*** | 1.4E-04 | 2.77 | Rattus norvegicus ubiquitin domain containing 1 (Ubtd1), mRNA [NM_001013153] |
| 163 | ***Capg*** | 2.5E-04 | 2.75 | Rattus norvegicus capping protein (actin filament), gelsolin-like (Capg), mRNA [NM_001013086] |
| 164 | ***Col16a1*** | 7.3E-03 | 2.74 | Rattus norvegicus collagen, type XVI, alpha 1 (Col16a1), mRNA [NM_001015033] |
| 165 | ***Grn*** | 1.6E-04 | 2.73 | Rattus norvegicus granulin (Grn), transcript variant 1, mRNA [NM_017113] |
| 166 | ***Apoe*** | 1.2E-03 | 2.65 | Rattus norvegicus apolipoprotein E (Apoe), transcript variant 2, mRNA [NM_138828] |
| 167 | ***Dennd2d*** | 4.3E-03 | 2.62 | Rattus norvegicus DENN/MADD domain containing 2D (Dennd2d), mRNA [NM_001107714] |
| 168 | ***Sel1l3*** | 2.6E-03 | 2.62 | PREDICTED: Rattus norvegicus sel-1 suppressor of lin-12-like 3 (C. elegans) (Sel1l3), transcript variant X1, mRNA [XM_006251033] |
| 169 | ***MGC95208*** | 4.7E-03 | 2.62 | Rattus norvegicus similar to 4930453N24Rik protein (MGC95208), mRNA [NM_001005552] |
| 170 | ***Rbm38*** | 1.2E-05 | 2.60 | Rattus norvegicus RNA binding motif protein 38 (Rbm38), mRNA [NM_001108965] |
| 171 | ***Birc3*** | 2.2E-03 | 2.59 | Rattus norvegicus baculoviral IAP repeat-containing 3 (Birc3), mRNA [NM_023987] |
| 172 | ***RT1-CE2*** | 4.0E-03 | 2.58 | Rattus norvegicus RT1 class I, locus CE2 (RT1-CE2), mRNA [NM_001008840] |
| 173 | ***Flnb*** | 4.9E-02 | 2.57 | Rattus norvegicus filamin B, beta (Flnb), mRNA [NM_001107288] |
| 174 | ***RGD1565166*** | 1.6E-03 | 2.56 | Rattus norvegicus similar to MGC45438 protein (RGD1565166), mRNA [NM_001105762] |
| 175 | ***LOC102554845*** | 2.0E-02 | 2.56 | PREDICTED: Rattus norvegicus uncharacterized LOC102554845 (LOC102554845), transcript variant X2, ncRNA [XR_362163] |
| 176 | ***Tyrobp*** | 8.9E-04 | 2.56 | Rattus norvegicus Tyro protein tyrosine kinase binding protein (Tyrobp), mRNA [NM_212525] |
| 177 | ***Lyl1*** | 3.1E-03 | 2.54 | Rattus norvegicus lymphoblastic leukemia derived sequence 1 (Lyl1), mRNA [NM_001007677] |
| 178 | ***Ripk3*** | 3.2E-04 | 2.54 | Rattus norvegicus receptor-interacting serine-threonine kinase 3 (Ripk3), mRNA [NM_139342] |
| 179 | ***MGC95208*** | 6.1E-03 | 2.52 | Rattus norvegicus similar to 4930453N24Rik protein (MGC95208), mRNA [NM_001005552] |
| 180 | ***Mapkapk3*** | 1.6E-03 | 2.52 | Rattus norvegicus mitogen-activated protein kinase-activated protein kinase 3 (Mapkapk3), mRNA [NM_001012127] |
| 181 | ***Set*** | 4.9E-03 | 2.52 | Rattus norvegicus SET nuclear oncogene (Set), mRNA [NM_001012504] |
| 182 | ***Prdx5*** | 8.4E-04 | 2.52 | Rattus norvegicus peroxiredoxin 5 (Prdx5), mRNA [NM_053610] |
| 183 | ***Cyp51*** | 2.7E-03 | 2.48 | Rattus norvegicus cytochrome P450, family 51 (Cyp51), mRNA [NM_012941] |
| 184 | ***Ubc*** | 4.2E-02 | 2.48 | Rattus norvegicus ubiquitin C (Ubc), mRNA [NM_017314] |
| 185 | ***Dnajc3*** | 5.4E-03 | 2.48 | Rattus norvegicus DnaJ (Hsp40) homolog, subfamily C, member 3 (Dnajc3), mRNA [NM_022232] |
| 186 | ***Csf2ra*** | 9.5E-05 | 2.48 | Rattus norvegicus colony stimulating factor 2 receptor, alpha, low-affinity (granulocyte-macrophage) (Csf2ra), mRNA [NM_001037660] |
| 187 | ***Ubb*** | 1.4E-02 | 2.47 | Rattus norvegicus ubiquitin B (Ubb), mRNA [NM_138895] |
| 188 | ***Ptcd3*** | 1.5E-02 | 2.47 | Rattus norvegicus Pentatricopeptide repeat domain 3 (Ptcd3), mRNA [NM_001134718] |
| 189 | ***Grb7*** | 2.0E-03 | 2.45 | Rattus norvegicus growth factor receptor bound protein 7 (Grb7), mRNA [NM_053403] |
| 190 | ***Npc1*** | 2.6E-02 | 2.45 | Rattus norvegicus Niemann-Pick disease, type C1 (Npc1), mRNA [NM_153624] |
| 191 | ***Snx20*** | 8.3E-04 | 2.43 | Rattus norvegicus sorting nexin 20 (Snx20), mRNA [NM_001024999] |
| 192 | ***Farsb*** | 2.4E-02 | 2.43 | Rattus norvegicus phenylalanyl-tRNA synthetase, beta subunit (Farsb), mRNA [NM_001004252] |
| 193 | ***Aars*** | 1.6E-02 | 2.43 | Rattus norvegicus alanyl-tRNA synthetase (Aars), mRNA [NM_001100517] |
| 194 | ***Socs3*** | 3.7E-03 | 2.42 | Rattus norvegicus suppressor of cytokine signaling 3 (Socs3), mRNA [NM_053565] |
| 195 | ***Slc11a2*** | 7.7E-03 | 2.41 | Rattus norvegicus solute carrier family 11 (proton-coupled divalent metal ion transporter), member 2 (Slc11a2), mRNA [NM_013173] |
| 196 | ***Ifi27l2b*** | 2.4E-02 | 2.41 | Rattus norvegicus interferon, alpha-inducible protein 27 like 2B (Ifi27l2b), mRNA [NM_206846] |
| 197 | ***Fuca1*** | 1.6E-03 | 2.40 | Rattus norvegicus fucosidase, alpha-L- 1, tissue (Fuca1), mRNA [NM_012562] |
| 198 | ***G6pd*** | 5.2E-05 | 2.40 | Rattus norvegicus glucose-6-phosphate dehydrogenase (G6pd), mRNA [NM_017006] |
| 199 | ***Esd*** | 3.7E-02 | 2.39 | Rattus norvegicus esterase D (Esd), transcript variant 1, mRNA [NM_001270865] |
| 200 | ***LOC688591*** | 5.0E-05 | 2.38 | PREDICTED: Rattus norvegicus ferritin light chain 1-like (LOC688591), misc_RNA [XR_006269] |
| 201 | ***Blvrb*** | 9.4E-04 | 2.37 | Rattus norvegicus biliverdin reductase B (flavin reductase (NADPH)) (Blvrb), mRNA [NM_001106236] |
| 202 | ***Dusp11*** | 2.3E-02 | 2.37 | Rattus norvegicus dual specificity phosphatase 11 (RNA/RNP complex 1-interacting) (Dusp11), mRNA [NM_001025650] |
| 203 | ***Dnajb4*** | 4.6E-02 | 2.36 | Rattus norvegicus DnaJ (Hsp40) homolog, subfamily B, member 4 (Dnajb4), mRNA [NM_001013076] |
| 204 | ***Myo5c*** | 2.1E-02 | 2.36 | PREDICTED: Rattus norvegicus myosin VC (Myo5c), transcript variant X1, mRNA [XM_006226477] |
| 205 | ***LOC100365047*** | 3.2E-02 | 2.36 | PREDICTED: Rattus norvegicus scavenger receptor class B, member 2-like (LOC100365047), mRNA [XM_006235988] |
| 206 | ***Rrbp1*** | 2.3E-02 | 2.35 | ribosome binding protein 1 [Source:MGI Symbol;Acc:MGI:1932395] [ENSRNOT00000007888] |
| 207 | ***Cct3*** | 1.5E-02 | 2.34 | Rattus norvegicus chaperonin containing Tcp1, subunit 3 (gamma) (Cct3), mRNA [NM_199091] |
| 208 | ***Mars*** | 1.7E-02 | 2.33 | Rattus norvegicus methionyl-tRNA synthetase (Mars), mRNA [NM_001127659] |
| 209 | ***Clu*** | 2.7E-03 | 2.33 | Rattus norvegicus clusterin (Clu), mRNA [NM_053021] |
| 210 | ***Acap1*** | 7.1E-03 | 2.30 | Rattus norvegicus ArfGAP with coiled-coil, ankyrin repeat and PH domains 1 (Acap1), mRNA [NM_001105796] |
| 211 | ***RT1-A1*** | 3.6E-02 | 2.29 | Rattus norvegicus RT1 class Ia, locus A1 (RT1-A1), mRNA [NM_001008827] |
| 212 | ***Psap*** | 7.6E-04 | 2.26 | Rattus norvegicus prosaposin (Psap), transcript variant 1, mRNA [NM_013013] |
| 213 | ***Gls*** | 4.4E-05 | 2.26 | Rattus norvegicus glutaminase (Gls), nuclear gene encoding mitochondrial protein, transcript variant 1, mRNA [NM_012569] |
| 214 | ***Fxr1*** | 7.8E-03 | 2.23 | Rattus norvegicus fragile X mental retardation, autosomal homolog 1 (Fxr1), mRNA [NM_001012179] |
| 215 | ***Sema4a*** | 3.3E-04 | 2.22 | Rattus norvegicus sema domain, immunoglobulin domain (Ig), transmembrane domain (TM) and short cytoplasmic domain, (semaphorin) 4A (Sema4a), mRNA [NM_001012078] |
| 216 | ***Tcp1*** | 1.4E-02 | 2.22 | Rattus norvegicus t-complex 1 (Tcp1), mRNA [NM_012670] |
| 217 | ***Hck*** | 2.4E-03 | 2.21 | Rattus norvegicus hemopoietic cell kinase (Hck), mRNA [NM_013185] |
| 218 | ***Gdpd5*** | 3.1E-04 | 2.21 | Protein Gdpd5; Putative uncharacterized protein RGD1559673_predicted [Source:UniProtKB/TrEMBL;Acc:G3V9L7] [ENSRNOT00000055321] |
| 219 | ***Slc35c1*** | 2.3E-03 | 2.21 | Rattus norvegicus solute carrier family 35 (GDP-fucose transporter), member C1 (Slc35c1), mRNA [NM_001107748] |
| 220 | ***Mafg*** | 2.7E-03 | 2.20 | Rattus norvegicus v-maf avian musculoaponeurotic fibrosarcoma oncogene homolog G (Mafg), mRNA [NM_022386] |
| 221 | ***Il10rb*** | 9.7E-03 | 2.20 | Rattus norvegicus interleukin 10 receptor, beta (Il10rb), mRNA [NM_001107111] |
| 222 | ***Snx18*** | 8.9E-03 | 2.20 | Rattus norvegicus sorting nexin 18 (Snx18), mRNA [NM_001107652] |
| 223 | ***Traf3*** | 3.2E-02 | 2.19 | Rattus norvegicus Tnf receptor-associated factor 3 (Traf3), mRNA [NM_001108724] |
| 224 | ***Lyz2*** | 8.1E-03 | 2.19 | Rattus norvegicus lysozyme 2 (Lyz2), mRNA [NM_012771] |
| 225 | ***Myo1f*** | 1.0E-03 | 2.18 | Rattus norvegicus myosin IF (Myo1f), mRNA [NM_001108076] |
| 226 | ***Gaa*** | 1.1E-04 | 2.18 | Rattus norvegicus glucosidase, alpha, acid (Gaa), mRNA [NM_199118] |
| 227 | ***St13*** | 3.7E-03 | 2.17 | Rattus norvegicus suppression of tumorigenicity 13 (St13), mRNA [NM_031122] |
| 228 | ***Myo1g*** | 1.1E-02 | 2.17 | Rattus norvegicus myosin IG (Myo1g), mRNA [NM_001134843] |
| 229 | ***Trim47*** | 9.6E-03 | 2.16 | Rattus norvegicus tripartite motif-containing 47 (Trim47), mRNA [NM_001109585] |
| 230 | ***Pdia3*** | 5.1E-03 | 2.16 | Rattus norvegicus protein disulfide isomerase family A, member 3 (Pdia3), mRNA [NM_017319] |
| 231 | ***Tcp1*** | 2.0E-02 | 2.16 | Rattus norvegicus t-complex 1 (Tcp1), mRNA [NM_012670] |
| 232 | ***Slc26a6*** | 3.1E-03 | 2.16 | Rattus norvegicus solute carrier family 26 (anion exchanger), member 6 (Slc26a6), mRNA [NM_001143817] |
| 233 | ***Mt2A*** | 1.8E-02 | 2.15 | Rattus norvegicus metallothionein 2A (Mt2A), mRNA [NM_001137564] |
| 234 | ***Plcb2*** | 3.1E-02 | 2.15 | Rattus norvegicus phospholipase C, beta 2 (Plcb2), mRNA [NM_053478] |
| 235 | ***Abcc1*** | 1.2E-02 | 2.15 | ATP-binding cassette, subfamily C (CFTR/MRP), member 1 (Abcc1), mRNA [Source:RefSeq mRNA;Acc:NM_022281] [ENSRNOT00000041358] |
| 236 | ***Cstb*** | 2.1E-04 | 2.14 | Rattus norvegicus cystatin B (stefin B) (Cstb), mRNA [NM_012838] |
| 237 | ***Abcc1*** | 3.4E-03 | 2.13 | Rattus norvegicus ATP-binding cassette, subfamily C (CFTR/MRP), member 1 (Abcc1), mRNA [NM_022281] |
| 238 | ***Ube2b*** | 7.1E-03 | 2.11 | Rattus norvegicus ubiquitin-conjugating enzyme E2B (Ube2b), mRNA [NM_031138] |
| 239 | ***Clint1*** | 8.7E-03 | 2.10 | PREDICTED: Rattus norvegicus clathrin interactor 1 (Clint1), transcript variant X1, mRNA [XM_006246149] |
| 240 | ***RT1-A1*** | 1.2E-02 | 2.10 | Rattus norvegicus RT1 class Ia, locus A1 (RT1-A1), mRNA [NM_001008827] |
| 241 | ***Cflar*** | 2.1E-02 | 2.09 | Rattus norvegicus CASP8 and FADD-like apoptosis regulator (Cflar), transcript variant 2, mRNA [NM_057138] |
| 242 | ***Casp4*** | 3.1E-02 | 2.09 | Rattus norvegicus caspase 4, apoptosis-related cysteine peptidase (Casp4), mRNA [NM_053736] |
| 243 | ***Heatr5a*** | 4.9E-06 | 2.08 | Protein Heatr5a [Source:UniProtKB/TrEMBL;Acc:F1LSK5] [ENSRNOT00000035309] |
| 244 | ***Dok3*** | 1.1E-03 | 2.07 | Rattus norvegicus docking protein 3 (Dok3), mRNA [NM_001107336] |
| 245 | ***Ccdc28a*** | 1.0E-03 | 2.07 | Rattus norvegicus coiled-coil domain containing 28A (Ccdc28a), mRNA [NM_001037789] |
| 246 | ***l7Rn6*** | 1.0E-02 | 2.06 | Rattus norvegicus lethal, Chr 7, Rinchik 6 (l7Rn6), mRNA [NM_001013897] |
| 247 | ***Ptges3*** | 3.8E-02 | 2.05 | Rattus norvegicus prostaglandin E synthase 3 (cytosolic) (Ptges3), mRNA [NM_001130989] |
| 248 | ***Arsb*** | 1.4E-02 | 2.05 | Rattus norvegicus arylsulfatase B (Arsb), mRNA [NM_033443] |
| 249 | ***Glrx*** | 5.4E-03 | 2.04 | Rattus norvegicus glutaredoxin (thioltransferase) (Glrx), mRNA [NM_022278] |
| 250 | ***Gngt2*** | 3.0E-04 | 2.04 | Rattus norvegicus guanine nucleotide binding protein (G protein), gamma transducing activity polypeptide 2 (Gngt2), mRNA [NM_001135767] |
| 251 | ***Dynll1*** | 5.0E-03 | 2.02 | Rattus norvegicus dynein light chain LC8-type 1 (Dynll1), mRNA [NM_053319] |
| 252 | ***Chchd2*** | 1.8E-02 | 2.02 | Rattus norvegicus coiled-coil-helix-coiled-coil-helix domain containing 2 (Chchd2), mRNA [NM_001015019] |
| 253 | ***Scarb1*** | 1.9E-02 | 2.02 | Rattus norvegicus scavenger receptor class B, member 1 (Scarb1), mRNA [NM_031541] |
| 254 | ***Lst1*** | 6.4E-05 | 2.01 | Rattus norvegicus leukocyte specific transcript 1 (Lst1), mRNA [NM_022634] |
| 255 | ***Coq10b*** | 7.4E-03 | 2.01 | Rattus norvegicus coenzyme Q10 homolog B (S. cerevisiae) (Coq10b), mRNA [NM_001009671] |
| 256 | ***Atp6v0c*** | 4.6E-04 | 2.01 | Rattus norvegicus ATPase, H+ transporting, lysosomal V0 subunit C (Atp6v0c), mRNA [NM_130823] |
| 257 | ***Uap1*** | 1.8E-03 | 2.01 | Rattus norvegicus UDP-N-acteylglucosamine pyrophosphorylase 1 (Uap1), mRNA [NM_001191930] |
| 258 | ***Hexb*** | 1.4E-05 | 2.00 | Rattus norvegicus hexosaminidase B (Hexb), mRNA [NM_001011946] |
| 259 | ***LOC683761*** | 1.6E-02 | 2.00 | PREDICTED: Rattus norvegicus RT1 class I histocompatibility antigen, AA alpha chain-like (LOC683761), partial mRNA [XM_006227186] |

**(B) ITO-exposed rats**

| Rank | ***GeneSymbol*** | p (ANOVA) | fold | Description |
| --- | --- | --- | --- | --- |
| 1 | ***Lcn2*** | 2.4E-06 | 91.77 | Rattus norvegicus lipocalin 2 (Lcn2), mRNA [NM_130741] |
| 2 | ***Trem2*** | 3.9E-06 | 60.01 | Rattus norvegicus triggering receptor expressed on myeloid cells 2 (Trem2), mRNA [NM_001106884] |
| 3 | ***Chia*** | 1.2E-05 | 56.87 | Rattus norvegicus chitinase, acidic (Chia), mRNA [NM_207586] |
| 4 | ***S100a9*** | 8.5E-08 | 46.46 | Rattus norvegicus S100 calcium binding protein A9 (S100a9), mRNA [NM_053587] |
| 5 | ***Bpifb1*** | 4.2E-04 | 41.46 | Rattus norvegicus BPI fold containing family B, member 1 (Bpifb1), mRNA [NM_001077680] |
| 6 | ***Lilrb4*** | 2.1E-04 | 33.91 | PREDICTED: Rattus norvegicus leukocyte immunoglobulin-like receptor, subfamily B, member 4 (Lilrb4), transcript variant X1, mRNA [XM_006228238] |
| 7 | ***Gpnmb*** | 1.2E-05 | 25.87 | Rattus norvegicus glycoprotein (transmembrane) nmb (Gpnmb), mRNA [NM_133298] |
| 8 | ***S100a8*** | 1.2E-05 | 21.97 | Rattus norvegicus S100 calcium binding protein A8 (S100a8), mRNA [NM_053822] |
| 9 | ***C3*** | 1.8E-04 | 21.66 | Rattus norvegicus complement component 3 (C3), mRNA [NM_016994] |
| 10 | ***Ly6i*** | 2.9E-05 | 19.76 | Rattus norvegicus lymphocyte antigen 6 complex, locus I (Ly6i), mRNA [NM_139257] |
| 11 | ***Hmox1*** | 3.7E-04 | 19.03 | Rattus norvegicus heme oxygenase (decycling) 1 (Hmox1), mRNA [NM_012580] |
| 12 | ***Tf*** | 5.0E-05 | 16.24 | Rattus norvegicus transferrin (Tf), mRNA [NM_001013110] |
| 13 | ***Cd68*** | 5.2E-05 | 13.64 | Rattus norvegicus Cd68 molecule (Cd68), mRNA [NM_001031638] |
| 14 | ***Ly6i*** | 8.4E-05 | 13.35 | lymphocyte antigen 6 complex, locus I (Ly6i), mRNA [Source:RefSeq mRNA;Acc:NM_139257] [ENSRNOT00000009395] |
| 15 | ***C4a*** | 1.2E-04 | 12.22 | Rattus norvegicus complement component 4A (Rodgers blood group) (C4a), mRNA [NM_031504] |
| 16 | ***Ifi30*** | 1.2E-04 | 12.16 | Rattus norvegicus interferon gamma inducible protein 30 (Ifi30), mRNA [NM_001030026] |
| 17 | ***Cfb*** | 6.6E-05 | 12.11 | Rattus norvegicus complement factor B (Cfb), mRNA [NM_212466] |
| 18 | ***Creg1*** | 3.9E-03 | 12.03 | Rattus norvegicus cellular repressor of E1A-stimulated genes 1 (Creg1), mRNA [NM_001105966] |
| 19 | ***Smpdl3a*** | 2.4E-04 | 11.89 | Rattus norvegicus sphingomyelin phosphodiesterase, acid-like 3A (Smpdl3a), mRNA [NM_001005539] |
| 20 | ***C1qa*** | 3.3E-04 | 11.28 | Rattus norvegicus complement component 1, q subcomponent, A chain (C1qa), mRNA [NM_001008515] |
| 21 | ***Qsox1*** | 1.2E-04 | 11.13 | Rattus norvegicus quiescin Q6 sulfhydryl oxidase 1 (Qsox1), transcript variant 2, mRNA [NM_053431] |
| 22 | ***Hp*** | 5.4E-04 | 10.53 | Rattus norvegicus haptoglobin (Hp), mRNA [NM_012582] |
| 23 | ***Fcgr2b*** | 3.4E-04 | 10.51 | Rattus norvegicus Fc fragment of IgG, low affinity IIb, receptor (CD32) (Fcgr2b), mRNA [NM_175756] |
| 24 | ***Scimp*** | 3.8E-04 | 10.00 | PREDICTED: Rattus norvegicus SLP adaptor and CSK interacting membrane protein (Scimp), mRNA [XM_003752341] |
| 25 | ***Fcgr2b*** | 5.4E-04 | 9.94 | Rattus norvegicus Fc fragment of IgG, low affinity IIb, receptor (CD32) (Fcgr2b), mRNA [NM_175756] |
| 26 | ***Slc16a3*** | 1.4E-03 | 9.60 | Rattus norvegicus solute carrier family 16 (monocarboxylate transporter), member 3 (Slc16a3), mRNA [NM_030834] |
| 27 | ***Serping1*** | 1.4E-03 | 9.44 | Rattus norvegicus serpin peptidase inhibitor, clade G (C1 inhibitor), member 1 (Serping1), mRNA [NM_199093] |
| 28 | ***RGD1564664*** | 4.3E-03 | 9.09 | Rattus norvegicus similar to LOC387763 protein (RGD1564664), mRNA [NM_001110055] |
| 29 | ***Serpinb1a*** | 2.3E-04 | 8.76 | Rattus norvegicus serine (or cysteine) proteinase inhibitor, clade B, member 1a (Serpinb1a), mRNA [NM_001031642] |
| 30 | ***Ctsh*** | 5.6E-04 | 8.73 | Rattus norvegicus cathepsin H (Ctsh), mRNA [NM_012939] |
| 31 | ***Lrg1*** | 9.5E-04 | 8.70 | Rattus norvegicus leucine-rich alpha-2-glycoprotein 1 (Lrg1), mRNA [NM_001009717] |
| 32 | ***Cfd*** | 2.5E-04 | 8.68 | Rattus norvegicus complement factor D (adipsin) (Cfd), mRNA [NM_001077642] |
| 33 | ***Sdf2l1*** | 4.1E-05 | 8.47 | Rattus norvegicus stromal cell-derived factor 2-like 1 (Sdf2l1), mRNA [NM_001109433] |
| 34 | ***Tmem173*** | 2.3E-05 | 8.41 | Rattus norvegicus transmembrane protein 173 (Tmem173), mRNA [NM_001109122] |
| 35 | ***Serpinb1a*** | 1.8E-04 | 8.33 | Rattus norvegicus serine (or cysteine) proteinase inhibitor, clade B, member 1a (Serpinb1a), mRNA [NM_001031642] |
| 36 | ***Ctsb*** | 2.8E-04 | 8.31 | Rattus norvegicus cathepsin B (Ctsb), mRNA [NM_022597] |
| 37 | ***Lgmn*** | 7.6E-04 | 8.25 | Rattus norvegicus legumain (Lgmn), mRNA [NM_022226] |
| 38 | ***Fabp5*** | 1.2E-04 | 8.18 | Rattus norvegicus fatty acid binding protein 5, epidermal (Fabp5), mRNA [NM_145878] |
| 39 | ***Atp6v1b2*** | 2.5E-04 | 8.05 | Rattus norvegicus ATPase, H transporting, lysosomal V1 subunit B2 (Atp6v1b2), mRNA [NM_057213] |
| 40 | ***LOC100909505*** | 1.4E-03 | 7.96 | PREDICTED: Rattus norvegicus N-acetylglucosamine-6-sulfatase-like (LOC100909505), mRNA [XM_003750325] |
| 41 | ***Ctsa*** | 5.5E-04 | 7.87 | Rattus norvegicus cathepsin A (Ctsa), mRNA [NM_001011959] |
| 42 | ***Cyba*** | 6.9E-04 | 7.86 | Rattus norvegicus cytochrome b-245, alpha polypeptide (Cyba), mRNA [NM_024160] |
| 43 | ***Grn*** | 6.3E-04 | 7.69 | Rattus norvegicus granulin (Grn), transcript variant 1, mRNA [NM_017113] |
| 44 | ***Atp1b1*** | 2.9E-03 | 7.50 | Rattus norvegicus ATPase, Na+/K+ transporting, beta 1 polypeptide (Atp1b1), mRNA [NM_013113] |
| 45 | ***Pld1*** | 6.6E-04 | 7.49 | Rattus norvegicus phospholipase D1 (Pld1), mRNA [NM_030992] |
| 46 | ***Ccl9*** | 5.4E-03 | 7.49 | Rattus norvegicus chemokine (C-C motif) ligand 9 (Ccl9), mRNA [NM_001012357] |
| 47 | ***Fetub*** | 2.0E-03 | 7.43 | Rattus norvegicus fetuin B (Fetub), mRNA [NM_053348] |
| 48 | ***Mgp*** | 9.8E-04 | 7.38 | Rattus norvegicus matrix Gla protein (Mgp), mRNA [NM_012862] |
| 49 | ***Endod1*** | 2.2E-03 | 7.25 | PREDICTED: Rattus norvegicus endonuclease domain containing 1 (Endod1), mRNA [XM_002729871] |
| 50 | ***Ctss*** | 2.2E-04 | 7.04 | Rattus norvegicus cathepsin S (Ctss), mRNA [NM_017320] |
| 51 | ***Sod2*** | 3.4E-04 | 6.91 | Rattus norvegicus superoxide dismutase 2, mitochondrial (Sod2), mRNA [NM_017051] |
| 52 | ***Cd53*** | 6.9E-04 | 6.70 | Rattus norvegicus Cd53 molecule (Cd53), mRNA [NM_012523] |
| 53 | ***Lamp1*** | 6.9E-04 | 6.69 | Rattus norvegicus lysosomal-associated membrane protein 1 (Lamp1), mRNA [NM_012857] |
| 54 | ***Lgals3*** | 8.1E-04 | 6.63 | Rattus norvegicus lectin, galactoside-binding, soluble, 3 (Lgals3), mRNA [NM_031832] |
| 55 | ***Rnaset2*** | 2.1E-04 | 6.59 | Rattus norvegicus ribonuclease T2 (Rnaset2), mRNA [NM_001106210] |
| 56 | ***Mgst1*** | 3.6E-03 | 6.52 | Rattus norvegicus microsomal glutathione S-transferase 1 (Mgst1), mRNA [NM_134349] |
| 57 | ***Fxyd5*** | 5.3E-04 | 6.51 | Rattus norvegicus FXYD domain-containing ion transport regulator 5 (Fxyd5), transcript variant 1, mRNA [NM_021909] |
| 58 | ***C2*** | 1.8E-02 | 6.49 | Rattus norvegicus complement component 2 (C2), mRNA [NM_172222] |
| 59 | ***Ncf4*** | 6.7E-04 | 6.49 | Rattus norvegicus neutrophil cytosolic factor 4 (Ncf4), mRNA [NM_001127304] |
| 60 | ***Cx3cl1*** | 1.6E-03 | 6.41 | Rattus norvegicus chemokine (C-X3-C motif) ligand 1 (Cx3cl1), mRNA [NM_134455] |
| 61 | ***Cxcl1*** | 8.3E-04 | 6.38 | Rattus norvegicus chemokine (C-X-C motif) ligand 1 (Cxcl1), mRNA [NM_030845] |
| 62 | ***Capg*** | 2.5E-04 | 6.34 | Rattus norvegicus capping protein (actin filament), gelsolin-like (Capg), mRNA [NM_001013086] |
| 63 | ***Hspa5*** | 5.5E-04 | 6.30 | Rattus norvegicus heat shock protein 5 (Hspa5), mRNA [NM_013083] |
| 64 | ***LOC498276*** | 4.2E-05 | 6.30 | Rattus norvegicus Fc gamma receptor II beta (LOC498276), mRNA [NM_001135992] |
| 65 | ***Cd83*** | 2.5E-03 | 6.29 | Rattus norvegicus CD83 molecule (Cd83), mRNA [NM_001108410] |
| 66 | ***Grn*** | 1.6E-04 | 6.26 | Rattus norvegicus granulin (Grn), transcript variant 1, mRNA [NM_017113] |
| 67 | ***Igsf8*** | 1.1E-04 | 6.20 | Rattus norvegicus immunoglobulin superfamily, member 8 (Igsf8), mRNA [NM_001014787] |
| 68 | ***Fam105a*** | 1.7E-03 | 6.17 | Rattus norvegicus family with sequence similarity 105, member A (Fam105a), mRNA [NM_001037648] |
| 69 | ***Ly6c*** | 7.8E-04 | 6.14 | Rattus norvegicus Ly6-C antigen (Ly6c), mRNA [NM_020103] |
| 70 | ***Cstb*** | 2.1E-04 | 6.13 | Rattus norvegicus cystatin B (stefin B) (Cstb), mRNA [NM_012838] |
| 71 | ***Cd63*** | 6.9E-04 | 6.07 | Rattus norvegicus Cd63 molecule (Cd63), mRNA [NM_017125] |
| 72 | ***C2*** | 5.2E-03 | 6.03 | Rattus norvegicus complement component 2 (C2), mRNA [NM_172222] |
| 73 | ***Litaf*** | 3.0E-04 | 6.00 | Rattus norvegicus lipopolysaccharide-induced TNF factor (Litaf), mRNA [NM_001105735] |
| 74 | ***Ctsd*** | 2.2E-03 | 5.97 | Rattus norvegicus cathepsin D (Ctsd), mRNA [NM_134334] |
| 75 | ***Gngt2*** | 2.3E-04 | 5.90 | Rattus norvegicus guanine nucleotide binding protein (G protein), gamma transducing activity polypeptide 2 (Gngt2), mRNA [NM_001135767] |
| 76 | ***Prdx5*** | 8.4E-04 | 5.79 | Rattus norvegicus peroxiredoxin 5 (Prdx5), mRNA [NM_053610] |
| 77 | ***Rnaset2*** | 1.7E-04 | 5.79 | Rattus norvegicus ribonuclease T2 (Rnaset2), mRNA [NM_001106210] |
| 78 | ***Tbxas1*** | 1.1E-02 | 5.78 | Rattus norvegicus thromboxane A synthase 1, platelet (Tbxas1), mRNA [NM_012687] |
| 79 | ***Bcl3*** | 3.2E-03 | 5.63 | Rattus norvegicus B-cell CLL/lymphoma 3 (Bcl3), mRNA [NM_001109422] |
| 80 | ***Pla2g15*** | 2.5E-02 | 5.58 | Rattus norvegicus phospholipase A2, group XV (Pla2g15), mRNA [NM_001004277] |
| 81 | ***Rac2*** | 1.4E-03 | 5.57 | Rattus norvegicus ras-related C3 botulinum toxin substrate 2 (rho family, small GTP binding protein Rac2) (Rac2), mRNA [NM_001008384] |
| 82 | ***Lgals3bp*** | 2.7E-03 | 5.52 | Rattus norvegicus lectin, galactoside-binding, soluble, 3 binding protein (Lgals3bp), mRNA [NM_139096] |
| 83 | ***Tmem86a*** | 2.0E-05 | 5.52 | Rattus norvegicus transmembrane protein 86A (Tmem86a), mRNA [NM_001135016] |
| 84 | ***Muc1*** | 5.7E-03 | 5.46 | Rattus norvegicus mucin 1, cell surface associated (Muc1), mRNA [NM_012602] |
| 85 | ***Atp1a1*** | 8.2E-04 | 5.41 | Rattus norvegicus ATPase, Na+/K+ transporting, alpha 1 polypeptide (Atp1a1), mRNA [NM_012504] |
| 86 | ***Mapkapk3*** | 1.6E-03 | 5.40 | Rattus norvegicus mitogen-activated protein kinase-activated protein kinase 3 (Mapkapk3), mRNA [NM_001012127] |
| 87 | ***Fasn*** | 8.6E-03 | 5.33 | Rattus norvegicus fatty acid synthase (Fasn), mRNA [NM_017332] |
| 88 | ***Apoe*** | 1.2E-03 | 5.32 | Rattus norvegicus apolipoprotein E (Apoe), transcript variant 2, mRNA [NM_138828] |
| 89 | ***Sftpa1*** | 2.7E-03 | 5.28 | Rattus norvegicus surfactant protein A1 (Sftpa1), transcript variant 2, mRNA [NM_017329] |
| 90 | ***Plod1*** | 1.7E-02 | 5.26 | Rattus norvegicus procollagen-lysine, 2-oxoglutarate 5-dioxygenase 1 (Plod1), mRNA [NM_053827] |
| 91 | ***RGD1563378*** | 8.7E-04 | 5.25 | PREDICTED: Rattus norvegicus ferritin heavy polypeptide-like 17-like (RGD1563378), transcript variant X1, mRNA [XM_006227337] |
| 92 | ***Tyrobp*** | 8.9E-04 | 5.24 | Rattus norvegicus Tyro protein tyrosine kinase binding protein (Tyrobp), mRNA [NM_212525] |
| 93 | ***Zfand2a*** | 2.3E-04 | 5.24 | Rattus norvegicus zinc finger, AN1-type domain 2A (Zfand2a), mRNA [NM_001008363] |
| 94 | ***Ubd*** | 2.6E-02 | 5.22 | Rattus norvegicus ubiquitin D (Ubd), mRNA [NM_053299] |
| 95 | ***Fam65b*** | 1.4E-04 | 5.12 | Rattus norvegicus family with sequence similarity 65, member B (Fam65b), mRNA [NM_001014009] |
| 96 | ***Naglu*** | 4.2E-04 | 5.04 | PREDICTED: Rattus norvegicus N-acetylglucosaminidase, alpha (Naglu), mRNA [XM_001081442] |
| 97 | ***Calr*** | 5.2E-03 | 5.01 | Rattus norvegicus calreticulin (Calr), mRNA [NM_022399] |
| 98 | ***LOC688591*** | 5.0E-05 | 4.99 | PREDICTED: Rattus norvegicus ferritin light chain 1-like (LOC688591), misc_RNA [XR_006269] |
| 99 | ***Ugt1a6*** | 2.1E-03 | 4.97 | Rattus norvegicus UDP glucuronosyltransferase 1 family, polypeptide A6 (Ugt1a6), transcript variant 1, mRNA [NM_001039691] |
| 100 | ***C1qb*** | 3.5E-04 | 4.96 | Rattus norvegicus complement component 1, q subcomponent, B chain (C1qb), mRNA [NM_019262] |
| 101 | ***LOC100360087*** | 4.7E-05 | 4.92 | PREDICTED: Rattus norvegicus ferritin light chain 1-like (LOC100360087), mRNA [XM_002726637] |
| 102 | ***Ltb*** | 3.7E-03 | 4.91 | Rattus norvegicus lymphotoxin beta (TNF superfamily, member 3) (Ltb), mRNA [NM_212507] |
| 103 | ***Paox*** | 3.6E-02 | 4.91 | Rattus norvegicus polyamine oxidase (exo-N4-amino) (Paox), mRNA [NM_001106311] |
| 104 | ***Trpv2*** | 6.1E-04 | 4.90 | Rattus norvegicus transient receptor potential cation channel, subfamily V, member 2 (Trpv2), transcript variant 1, mRNA [NM_017207] |
| 105 | ***RT1-Bb*** | 4.9E-04 | 4.89 | Rattus norvegicus RT1 class II, locus Bb (RT1-Bb), mRNA [NM_001004084] |
| 106 | ***Itgb2*** | 2.2E-02 | 4.88 | Rattus norvegicus integrin, beta 2 (Itgb2), mRNA [NM_001037780] |
| 107 | ***RGD1561520*** | 8.9E-05 | 4.86 | PREDICTED: Rattus norvegicus ferritin light chain 1-like (RGD1561520), mRNA [XM_345159] |
| 108 | ***LOC688090*** | 3.8E-03 | 4.85 | Rattus norvegicus similar to RT1 class II histocompatibility antigen, B-1 beta chain precursor (RT1.B-beta(1)) (LOC688090), mRNA [NM_001101017] |
| 109 | ***Snx20*** | 8.3E-04 | 4.83 | Rattus norvegicus sorting nexin 20 (Snx20), mRNA [NM_001024999] |
| 110 | ***Ftl1*** | 5.4E-05 | 4.82 | Rattus norvegicus ferritin light chain 1 (Ftl1), mRNA [NM_022500] |
| 111 | ***Hexb*** | 1.4E-05 | 4.80 | Rattus norvegicus hexosaminidase B (Hexb), mRNA [NM_001011946] |
| 112 | ***Herpud1*** | 1.3E-02 | 4.77 | Rattus norvegicus homocysteine-inducible, endoplasmic reticulum stress-inducible, ubiquitin-like domain member 1 (Herpud1), mRNA [NM_053523] |
| 113 | ***Rasa4*** | 1.4E-03 | 4.76 | PREDICTED: Rattus norvegicus RAS p21 protein activator 4 (Rasa4), transcript variant 2, mRNA [XM_002724809] |
| 114 | ***Cd302*** | 3.8E-03 | 4.75 | Rattus norvegicus CD302 molecule (Cd302), mRNA [NM_001013916] |
| 115 | ***Lgals1*** | 3.2E-04 | 4.74 | Rattus norvegicus lectin, galactoside-binding, soluble, 1 (Lgals1), mRNA [NM_019904] |
| 116 | ***Bpifa5*** | 8.3E-03 | 4.73 | Rattus norvegicus BPI fold containing family A, member 5 (Bpifa5), mRNA [NM_001107792] |
| 117 | ***Slc15a3*** | 1.3E-02 | 4.70 | Rattus norvegicus solute carrier family 15 (oligopeptide transporter), member 3 (Slc15a3), mRNA [NM_139341] |
| 118 | ***Myo5a*** | 2.2E-03 | 4.70 | Rattus norvegicus myosin VA (Myo5a), mRNA [NM_022178] |
| 119 | ***B4galt5*** | 6.8E-03 | 4.68 | Rattus norvegicus UDP-Gal:betaGlcNAc beta 1,4-galactosyltransferase, polypeptide 5 (B4galt5), mRNA [NM_001108608] |
| 120 | ***Mmp14*** | 4.3E-03 | 4.62 | Rattus norvegicus matrix metallopeptidase 14 (membrane-inserted) (Mmp14), mRNA [NM_031056] |
| 121 | ***Sftpd*** | 2.4E-03 | 4.60 | Rattus norvegicus surfactant protein D (Sftpd), mRNA [NM_012878] |
| 122 | ***Brms1l*** | 6.4E-03 | 4.59 | Rattus norvegicus breast cancer metastasis-suppressor 1-like (Brms1l), mRNA [NM_001106731] |
| 123 | ***RGD1565002*** | 3.1E-02 | 4.59 | Rattus norvegicus similar to Dehydrogenase/reductase SDR family member 7 precursor (Retinal short-chain dehydrogenase/reductase 4) (RGD1565002), mRNA [NM_001271394] |
| 124 | ***Cxcl3*** | 6.3E-03 | 4.51 | Rattus norvegicus chemokine (C-X-C motif) ligand 3 (Cxcl3), mRNA [NM_138522] |
| 125 | ***Dgat2*** | 1.3E-02 | 4.49 | Rattus norvegicus diacylglycerol O-acyltransferase 2 (Dgat2), mRNA [NM_001012345] |
| 126 | ***Itgam*** | 4.0E-03 | 4.48 | integrin, alpha M (Itgam), mRNA [Source:RefSeq mRNA;Acc:NM_012711] [ENSRNOT00000026748] |
| 127 | ***Jak3*** | 4.0E-02 | 4.44 | Rattus norvegicus Janus kinase 3 (Jak3), mRNA [NM_012855] |
| 128 | ***Cmtm7*** | 6.9E-03 | 4.44 | Rattus norvegicus CKLF-like MARVEL transmembrane domain containing 7 (Cmtm7), mRNA [NM_001109300] |
| 129 | ***Atp6v0c*** | 4.6E-04 | 4.41 | Rattus norvegicus ATPase, H+ transporting, lysosomal V0 subunit C (Atp6v0c), mRNA [NM_130823] |
| 130 | ***Lipa*** | 9.7E-04 | 4.38 | Rattus norvegicus lipase A, lysosomal acid, cholesterol esterase (Lipa), mRNA [NM_012732] |
| 131 | ***Col16a1*** | 7.3E-03 | 4.34 | Rattus norvegicus collagen, type XVI, alpha 1 (Col16a1), mRNA [NM_001015033] |
| 132 | ***P4hb*** | 1.7E-02 | 4.34 | Rattus norvegicus prolyl 4-hydroxylase, beta polypeptide (P4hb), mRNA [NM_012998] |
| 133 | ***Myo1f*** | 1.0E-03 | 4.32 | Rattus norvegicus myosin IF (Myo1f), mRNA [NM_001108076] |
| 134 | ***Slc16a11*** | 8.8E-03 | 4.32 | Rattus norvegicus solute carrier family 16, member 11 (Slc16a11), mRNA [NM_001105797] |
| 135 | ***Lyl1*** | 3.1E-03 | 4.31 | Rattus norvegicus lymphoblastic leukemia derived sequence 1 (Lyl1), mRNA [NM_001007677] |
| 136 | ***Aldh3b1*** | 6.9E-04 | 4.31 | Rattus norvegicus aldehyde dehydrogenase 3 family, member B1 (Aldh3b1), mRNA [NM_001006998] |
| 137 | ***Manba*** | 3.0E-02 | 4.31 | Rattus norvegicus mannosidase, beta A, lysosomal (Manba), mRNA [NM_001031655] |
| 138 | ***Gaa*** | 1.1E-04 | 4.31 | Rattus norvegicus glucosidase, alpha, acid (Gaa), mRNA [NM_199118] |
| 139 | ***Pnpla7*** | 6.3E-03 | 4.29 | Rattus norvegicus patatin-like phospholipase domain containing 7 (Pnpla7), mRNA [NM_144738] |
| 140 | ***Npc2*** | 2.8E-04 | 4.29 | Rattus norvegicus Niemann-Pick disease, type C2 (Npc2), mRNA [NM_173118] |
| 141 | ***Rassf4*** | 8.8E-03 | 4.25 | Rattus norvegicus Ras association (RalGDS/AF-6) domain family member 4 (Rassf4), mRNA [NM_001024275] |
| 142 | ***Fuca1*** | 1.6E-03 | 4.24 | Rattus norvegicus fucosidase, alpha-L- 1, tissue (Fuca1), mRNA [NM_012562] |
| 143 | ***Cd74*** | 8.3E-03 | 4.23 | Rattus norvegicus Cd74 molecule, major histocompatibility complex, class II invariant chain (Cd74), mRNA [NM_013069] |
| 144 | ***Man2b2*** | 6.5E-03 | 4.22 | Rattus norvegicus mannosidase, alpha, class 2B, member 2 (Man2b2), mRNA [NM_001134971] |
| 145 | ***Prdx1*** | 4.7E-04 | 4.19 | Rattus norvegicus peroxiredoxin 1 (Prdx1), mRNA [NM_057114] |
| 146 | ***Il1b*** | 2.4E-03 | 4.19 | Rattus norvegicus interleukin 1 beta (Il1b), mRNA [NM_031512] |
| 147 | ***Apobr*** | 4.8E-03 | 4.16 | Rattus norvegicus apolipoprotein B receptor (Apobr), mRNA [NM_001109154] |
| 148 | ***Lst1*** | 6.4E-05 | 4.15 | Rattus norvegicus leukocyte specific transcript 1 (Lst1), mRNA [NM_022634] |
| 149 | ***B3gnt7*** | 2.1E-04 | 4.14 | Rattus norvegicus UDP-GlcNAc:betaGal beta-1,3-N-acetylglucosaminyltransferase 7 (B3gnt7), mRNA [NM_001012134] |
| 150 | ***Slc2a1*** | 8.1E-03 | 4.13 | Rattus norvegicus solute carrier family 2 (facilitated glucose transporter), member 1 (Slc2a1), mRNA [NM_138827] |
| 151 | ***Npc1*** | 2.6E-02 | 4.13 | Rattus norvegicus Niemann-Pick disease, type C1 (Npc1), mRNA [NM_153624] |
| 152 | ***Tmem14c*** | 2.1E-04 | 4.12 | Rattus norvegicus transmembrane protein 14C (Tmem14c), transcript variant 1, mRNA [NM_134395] |
| 153 | ***RGD1565166*** | 1.6E-03 | 4.09 | Rattus norvegicus similar to MGC45438 protein (RGD1565166), mRNA [NM_001105762] |
| 154 | ***Acp5*** | 8.8E-03 | 4.08 | Rattus norvegicus acid phosphatase 5, tartrate resistant (Acp5), transcript variant 1, mRNA [NM_019144] |
| 155 | ***Hcst*** | 3.4E-04 | 4.08 | Rattus norvegicus hematopoietic cell signal transducer (Hcst), mRNA [NM_001005900] |
| 156 | ***Dnajc3*** | 5.4E-03 | 4.07 | Rattus norvegicus DnaJ (Hsp40) homolog, subfamily C, member 3 (Dnajc3), mRNA [NM_022232] |
| 157 | ***Tcirg1*** | 2.7E-03 | 4.06 | Rattus norvegicus T-cell, immune regulator 1, ATPase, H+ transporting, lysosomal V0 subunit A3 (Tcirg1), mRNA [NM_199089] |
| 158 | ***Mme*** | 2.5E-02 | 4.05 | Rattus norvegicus membrane metallo-endopeptidase (Mme), mRNA [NM_012608] |
| 159 | ***Ssr4*** | 5.9E-03 | 4.05 | Rattus norvegicus signal sequence receptor, delta (Ssr4), mRNA [NM_017199] |
| 160 | ***RGD1359108*** | 2.5E-02 | 4.03 | Rattus norvegicus similar to RIKEN cDNA 3110043O21 (RGD1359108), mRNA [NM_001007702] |
| 161 | ***Ifitm1*** | 3.2E-03 | 4.03 | Rattus norvegicus interferon induced transmembrane protein 1 (Ifitm1), mRNA [NM_001106314] |
| 162 | ***Sqle*** | 2.8E-02 | 4.03 | Rattus norvegicus squalene epoxidase (Sqle), mRNA [NM_017136] |
| 163 | ***Dnase2*** | 2.9E-03 | 4.01 | Rattus norvegicus deoxyribonuclease II, lysosomal (Dnase2), mRNA [NM_138539] |
| 164 | ***LOC678766*** | 1.0E-02 | 4.00 | PREDICTED: Rattus norvegicus rho-related GTP-binding protein RhoU-like (LOC678766), mRNA [XM_006222804] |
| 165 | ***Me1*** | 1.9E-02 | 3.98 | Rattus norvegicus malic enzyme 1, NADP(+)-dependent, cytosolic (Me1), mRNA [NM_012600] |
| 166 | ***RT1-Bb*** | 2.0E-02 | 3.98 | Rattus norvegicus RT1 class II, locus Bb (RT1-Bb), mRNA [NM_001004084] |
| 167 | ***Uap1l1*** | 1.0E-02 | 3.98 | Rattus norvegicus UDP-N-acteylglucosamine pyrophosphorylase 1-like 1 (Uap1l1), mRNA [NM_001134516] |
| 168 | ***Hck*** | 2.4E-03 | 3.97 | Rattus norvegicus hemopoietic cell kinase (Hck), mRNA [NM_013185] |
| 169 | ***Dennd2d*** | 4.3E-03 | 3.97 | Rattus norvegicus DENN/MADD domain containing 2D (Dennd2d), mRNA [NM_001107714] |
| 170 | ***Chordc1*** | 4.2E-05 | 3.96 | Rattus norvegicus cysteine and histidine-rich domain (CHORD)-containing 1 (Chordc1), mRNA [NM_001108128] |
| 171 | ***Atp6v0b*** | 5.5E-03 | 3.96 | Rattus norvegicus ATPase, H+ transporting, lysosomal V0 subunit B (Atp6v0b), mRNA [NM_001106681] |
| 172 | ***RGD1303130*** | 3.3E-02 | 3.95 | Rattus norvegicus kidney predominant protein NCU-G1 (RGD1303130), mRNA [NM_001004226] |
| 173 | ***Ckb*** | 2.6E-03 | 3.92 | Rattus norvegicus creatine kinase, brain (Ckb), mRNA [NM_012529] |
| 174 | ***Sidt2*** | 1.8E-02 | 3.91 | Rattus norvegicus SID1 transmembrane family, member 2 (Sidt2), mRNA [NM_001108142] |
| 175 | ***LOC689489*** | 2.6E-04 | 3.91 | Protein LOC689489 [Source:UniProtKB/TrEMBL;Acc:D4AAZ5] [ENSRNOT00000035746] |
| 176 | ***Aco1*** | 4.9E-02 | 3.90 | Rattus norvegicus aconitase 1, soluble (Aco1), mRNA [NM_017321] |
| 177 | ***Fcer1g*** | 6.8E-03 | 3.86 | Rattus norvegicus Fc fragment of IgE, high affinity I, receptor for; gamma polypeptide (Fcer1g), mRNA [NM_001131001] |
| 178 | ***Gda*** | 1.3E-02 | 3.85 | Rattus norvegicus guanine deaminase (Gda), mRNA [NM_031776] |
| 179 | ***Glrx*** | 5.4E-03 | 3.83 | Rattus norvegicus glutaredoxin (thioltransferase) (Glrx), mRNA [NM_022278] |
| 180 | ***Pdia6*** | 5.6E-03 | 3.82 | Rattus norvegicus protein disulfide isomerase family A, member 6 (Pdia6), mRNA [NM_001004442] |
| 181 | ***Ccl3*** | 2.6E-02 | 3.81 | Rattus norvegicus chemokine (C-C motif) ligand 3 (Ccl3), mRNA [NM_013025] |
| 182 | ***Ttyh2*** | 8.2E-04 | 3.81 | PREDICTED: Rattus norvegicus tweety family member 2 (Ttyh2), mRNA [XM_006220891] |
| 183 | ***Gsta1*** | 3.3E-03 | 3.80 | Rattus norvegicus glutathione S-transferase alpha 1 (Gsta1), mRNA [NM_031509] |
| 184 | ***Blvrb*** | 9.4E-04 | 3.80 | Rattus norvegicus biliverdin reductase B (flavin reductase (NADPH)) (Blvrb), mRNA [NM_001106236] |
| 185 | ***Cotl1*** | 3.2E-03 | 3.80 | Rattus norvegicus coactosin-like 1 (Dictyostelium) (Cotl1), mRNA [NM_001108452] |
| 186 | ***Clu*** | 2.7E-03 | 3.78 | Rattus norvegicus clusterin (Clu), mRNA [NM_053021] |
| 187 | ***Csf2ra*** | 9.5E-05 | 3.77 | Rattus norvegicus colony stimulating factor 2 receptor, alpha, low-affinity (granulocyte-macrophage) (Csf2ra), mRNA [NM_001037660] |
| 188 | ***Crlf2*** | 3.9E-03 | 3.76 | Rattus norvegicus cytokine receptor-like factor 2 (Crlf2), mRNA [NM_134465] |
| 189 | ***Igf2r*** | 3.3E-03 | 3.73 | Rattus norvegicus insulin-like growth factor 2 receptor (Igf2r), mRNA [NM_012756] |
| 190 | ***Oxct1*** | 2.2E-02 | 3.72 | Rattus norvegicus 3-oxoacid CoA transferase 1 (Oxct1), mRNA [NM_001127580] |
| 191 | ***Glipr1*** | 4.6E-03 | 3.71 | Rattus norvegicus GLI pathogenesis-related 1 (Glipr1), mRNA [NM_001011987] |
| 192 | ***Rragc*** | 5.3E-03 | 3.71 | Rattus norvegicus Ras-related GTP binding C (Rragc), mRNA [NM_001048184] |
| 193 | ***Tec*** | 3.9E-03 | 3.71 | Rattus norvegicus tec protein tyrosine kinase (Tec), mRNA [NM_053432] |
| 194 | ***Slc11a2*** | 7.7E-03 | 3.71 | Rattus norvegicus solute carrier family 11 (proton-coupled divalent metal ion transporter), member 2 (Slc11a2), mRNA [NM_013173] |
| 195 | ***Folr2*** | 1.2E-02 | 3.70 | Rattus norvegicus folate receptor 2 (fetal) (Folr2), mRNA [NM_001106283] |
| 196 | ***Timp1*** | 1.9E-03 | 3.70 | Rattus norvegicus TIMP metallopeptidase inhibitor 1 (Timp1), mRNA [NM_053819] |
| 197 | ***Pigr*** | 1.7E-02 | 3.70 | Rattus norvegicus polymeric immunoglobulin receptor (Pigr), mRNA [NM_012723] |
| 198 | ***Ncf1*** | 6.6E-03 | 3.68 | Rattus norvegicus neutrophil cytosolic factor 1 (Ncf1), mRNA [NM_053734] |
| 199 | ***Mt1a*** | 2.2E-04 | 3.68 | Rattus norvegicus metallothionein 1a (Mt1a), mRNA [NM_138826] |
| 200 | ***Ssr2*** | 1.4E-02 | 3.67 | Rattus norvegicus signal sequence receptor, beta (Ssr2), mRNA [NM_001106442] |
| 201 | ***Ubtd1*** | 1.4E-04 | 3.66 | Rattus norvegicus ubiquitin domain containing 1 (Ubtd1), mRNA [NM_001013153] |
| 202 | ***Msln*** | 4.3E-02 | 3.65 | Rattus norvegicus mesothelin (Msln), mRNA [NM_031658] |
| 203 | ***G6pd*** | 5.2E-05 | 3.65 | Rattus norvegicus glucose-6-phosphate dehydrogenase (G6pd), mRNA [NM_017006] |
| 204 | ***Hyou1*** | 1.4E-02 | 3.64 | Rattus norvegicus hypoxia up-regulated 1 (Hyou1), transcript variant 1, mRNA [NM_138867] |
| 205 | ***Tor4a*** | 1.8E-02 | 3.63 | Rattus norvegicus torsin family 4, member A (Tor4a), mRNA [NM_001107816] |
| 206 | ***Sema4a*** | 3.3E-04 | 3.62 | Rattus norvegicus sema domain, immunoglobulin domain (Ig), transmembrane domain (TM) and short cytoplasmic domain, (semaphorin) 4A (Sema4a), mRNA [NM_001012078] |
| 207 | ***Gsta3*** | 8.9E-04 | 3.62 | Rattus norvegicus glutathione S-transferase alpha 3 (Gsta3), transcript variant 1, mRNA [NM_001009920] |
| 208 | ***Gngt2*** | 3.0E-04 | 3.61 | Rattus norvegicus guanine nucleotide binding protein (G protein), gamma transducing activity polypeptide 2 (Gngt2), mRNA [NM_001135767] |
| 209 | ***Farsb*** | 2.4E-02 | 3.60 | Rattus norvegicus phenylalanyl-tRNA synthetase, beta subunit (Farsb), mRNA [NM_001004252] |
| 210 | ***Arpc3*** | 1.1E-02 | 3.57 | Rattus norvegicus actin related protein 2/3 complex, subunit 3 (Arpc3), mRNA [NM_001105933] |
| 211 | ***Ccdc93*** | 2.8E-03 | 3.57 | Rattus norvegicus coiled-coil domain containing 93 (Ccdc93), mRNA [NM_001024997] |
| 212 | ***Srebf1*** | 8.8E-03 | 3.56 | Rattus norvegicus sterol regulatory element binding transcription factor 1 (Srebf1), transcript variant 1, mRNA [NM_001276707] |
| 213 | ***Hebp1*** | 1.3E-04 | 3.55 | Rattus norvegicus heme binding protein 1 (Hebp1), mRNA [NM_001108651] |
| 214 | ***Noxo1*** | 9.9E-03 | 3.55 | Rattus norvegicus NADPH oxidase organizer 1 (Noxo1), mRNA [NM_001106986] |
| 215 | ***Actr2*** | 1.7E-02 | 3.54 | Rattus norvegicus ARP2 actin-related protein 2 homolog (yeast) (Actr2), mRNA [NM_001009268] |
| 216 | ***Cxcl17*** | 1.8E-02 | 3.53 | Rattus norvegicus chemokine (C-X-C motif) ligand 17 (Cxcl17), mRNA [NM_001107491] |
| 217 | ***Cxcl3*** | 5.1E-04 | 3.53 | PREDICTED: Rattus norvegicus chemokine (C-X-C motif) ligand 3 (Cxcl3), transcript variant X1, mRNA [XM_006250721] |
| 218 | ***Tkt*** | 4.5E-03 | 3.52 | Rattus norvegicus transketolase (Tkt), mRNA [NM_022592] |
| 219 | ***Atp2a2*** | 1.9E-02 | 3.52 | Rattus norvegicus ATPase, Ca++ transporting, cardiac muscle, slow twitch 2 (Atp2a2), transcript variant 3, mRNA [NM_001110823] |
| 220 | ***Gba*** | 7.0E-03 | 3.51 | Rattus norvegicus glucosidase, beta, acid (Gba), mRNA [NM_001127639] |
| 221 | ***RT1-T24-1*** | 2.6E-02 | 3.50 | Rattus norvegicus RT1 class I, locus T24, gene 1 (RT1-T24-1), mRNA [NM_001008858] |
| 222 | ***Manf*** | 7.0E-05 | 3.50 | Rattus norvegicus mesencephalic astrocyte-derived neurotrophic factor (Manf), mRNA [NM_001108183] |
| 223 | ***Fxyd3*** | 1.4E-02 | 3.49 | Rattus norvegicus FXYD domain-containing ion transport regulator 3 (Fxyd3), mRNA [NM_172317] |
| 224 | ***Bag3*** | 6.0E-04 | 3.49 | Rattus norvegicus Bcl2-associated athanogene 3 (Bag3), mRNA [NM_001011936] |
| 225 | ***Ifi27l2b*** | 7.6E-03 | 3.48 | Rattus norvegicus interferon, alpha-inducible protein 27 like 2B (Ifi27l2b), mRNA [NM_206846] |
| 226 | ***Emp3*** | 8.1E-03 | 3.47 | Rattus norvegicus epithelial membrane protein 3 (Emp3), mRNA [NM_030847] |
| 227 | ***Abr*** | 2.2E-02 | 3.47 | Rattus norvegicus active BCR-related (Abr), mRNA [NM_001105814] |
| 228 | ***P2ry6*** | 3.9E-03 | 3.47 | Rattus norvegicus pyrimidinergic receptor P2Y, G-protein coupled, 6 (P2ry6), mRNA [NM_057124] |
| 229 | ***Dnaja1*** | 4.9E-05 | 3.47 | Rattus norvegicus DnaJ (Hsp40) homolog, subfamily A, member 1 (Dnaja1), mRNA [NM_022934] |
| 230 | ***Slc39a13*** | 4.3E-02 | 3.47 | Rattus norvegicus solute carrier family 39 (zinc transporter), member 13 (Slc39a13), mRNA [NM_001039196] |
| 231 | ***RT1-Da*** | 1.0E-02 | 3.45 | Rattus norvegicus RT1 class II, locus Da (RT1-Da), mRNA [NM_001008847] |
| 232 | ***Slc31a2*** | 1.3E-02 | 3.44 | Rattus norvegicus solute carrier family 31 (copper transporter), member 2 (Slc31a2), mRNA [NM_001033693] |
| 233 | ***Psap*** | 7.6E-04 | 3.44 | Rattus norvegicus prosaposin (Psap), transcript variant 1, mRNA [NM_013013] |
| 234 | ***RT1-CE16*** | 9.1E-03 | 3.44 | Rattus norvegicus RT1 class I, locus CE16 (RT1-CE16), mRNA [NM_001008839] |
| 235 | ***Alas1*** | 1.0E-02 | 3.43 | Rattus norvegicus aminolevulinate, delta-, synthase 1 (Alas1), mRNA [NM_024484] |
| 236 | ***LOC685157*** | 2.7E-04 | 3.42 | Rattus norvegicus similar to paired immunoglobin-like type 2 receptor beta (LOC685157), mRNA [NM_001115044] |
| 237 | ***Slpi*** | 2.6E-02 | 3.42 | Rattus norvegicus secretory leukocyte peptidase inhibitor (Slpi), mRNA [NM_053372] |
| 238 | ***Ghitm*** | 2.7E-02 | 3.40 | Rattus norvegicus growth hormone inducible transmembrane protein (Ghitm), mRNA [NM_001005908] |
| 239 | ***Cfh*** | 4.0E-02 | 3.39 | Rattus norvegicus complement factor H (Cfh), mRNA [NM_130409] |
| 240 | ***Rpia*** | 4.0E-02 | 3.37 | Rattus norvegicus ribose 5-phosphate isomerase A (Rpia), mRNA [NM_001108632] |
| 241 | ***Mpzl2*** | 4.6E-02 | 3.35 | Rattus norvegicus myelin protein zero-like 2 (Mpzl2), mRNA [NM_001106818] |
| 242 | ***Irf5*** | 1.1E-03 | 3.34 | Rattus norvegicus interferon regulatory factor 5 (Irf5), mRNA [NM_001106586] |
| 243 | ***Atp6ap1*** | 2.7E-02 | 3.33 | Rattus norvegicus ATPase, H+ transporting, lysosomal accessory protein 1 (Atp6ap1), mRNA [NM_031785] |
| 244 | ***Trim47*** | 9.6E-03 | 3.33 | Rattus norvegicus tripartite motif-containing 47 (Trim47), mRNA [NM_001109585] |
| 245 | ***Tnfsf13*** | 1.3E-03 | 3.32 | Rattus norvegicus tumor necrosis factor (ligand) superfamily, member 13 (Tnfsf13), mRNA [NM_001009623] |
| 246 | ***Arsb*** | 1.4E-02 | 3.31 | Rattus norvegicus arylsulfatase B (Arsb), mRNA [NM_033443] |
| 247 | ***Defb4*** | 5.9E-03 | 3.31 | Rattus norvegicus defensin beta 4 (Defb4), mRNA [NM_022544] |
| 248 | ***Pdia3*** | 5.1E-03 | 3.31 | Rattus norvegicus protein disulfide isomerase family A, member 3 (Pdia3), mRNA [NM_017319] |
| 249 | ***Fcer1g*** | 6.0E-03 | 3.30 | Rattus norvegicus Fc fragment of IgE, high affinity I, receptor for; gamma polypeptide (Fcer1g), mRNA [NM_001131001] |
| 250 | ***Slc35c1*** | 2.3E-03 | 3.29 | Rattus norvegicus solute carrier family 35 (GDP-fucose transporter), member C1 (Slc35c1), mRNA [NM_001107748] |
| 251 | ***Cd6*** | 1.2E-03 | 3.28 | Rattus norvegicus Cd6 molecule (Cd6), mRNA [NM_175577] |
| 252 | ***Nudt9*** | 1.1E-02 | 3.28 | Rattus norvegicus nudix (nucleoside diphosphate linked moiety X)-type motif 9 (Nudt9), mRNA [NM_001006991] |
| 253 | ***Gdpd5*** | 3.1E-04 | 3.28 | Protein Gdpd5; Putative uncharacterized protein RGD1559673_predicted [Source:UniProtKB/TrEMBL;Acc:G3V9L7] [ENSRNOT00000055321] |
| 254 | ***Eno1*** | 1.4E-03 | 3.28 | Rattus norvegicus enolase 1, (alpha), mRNA (cDNA clone MGC:93770 IMAGE:7108936), complete cds. [BC090069] |
| 255 | ***Ctse*** | 4.2E-02 | 3.28 | Rattus norvegicus cathepsin E (Ctse), mRNA [NM_012938] |
| 256 | ***Rnasek*** | 1.4E-02 | 3.27 | Rattus norvegicus ribonuclease, RNase K (Rnasek), mRNA [NM_001137561] |
| 257 | ***Trappc9*** | 1.8E-02 | 3.26 | Rattus norvegicus trafficking protein particle complex 9 (Trappc9), mRNA [NM_001034156] |
| 258 | ***G0s2*** | 3.0E-02 | 3.26 | Rattus norvegicus G0/G1switch 2 (G0s2), mRNA [NM_001009632] |
| 259 | ***Hexb*** | 1.2E-04 | 3.25 | Rattus norvegicus hexosaminidase B (Hexb), mRNA [NM_001011946] |
| 260 | ***Emc6*** | 9.8E-03 | 3.24 | Rattus norvegicus ER membrane protein complex subunit 6 (Emc6), mRNA [NM_001105806] |
| 261 | ***Dok3*** | 1.1E-03 | 3.24 | Rattus norvegicus docking protein 3 (Dok3), mRNA [NM_001107336] |
| 262 | ***Ccng1*** | 4.8E-04 | 3.24 | Rattus norvegicus cyclin G1 (Ccng1), mRNA [NM_012923] |
| 263 | ***Tmbim6*** | 1.7E-02 | 3.23 | Rattus norvegicus transmembrane BAX inhibitor motif containing 6 (Tmbim6), mRNA [NM_019381] |
| 264 | ***Slc25a44*** | 3.2E-02 | 3.21 | Rattus norvegicus solute carrier family 25, member 44 (Slc25a44), mRNA [NM_001108947] |
| 265 | ***Vim*** | 3.3E-03 | 3.21 | Rattus norvegicus vimentin (Vim), mRNA [NM_031140] |
| 266 | ***Coro7*** | 1.4E-02 | 3.19 | Rattus norvegicus coronin 7 (Coro7), mRNA [NM_001191639] |
| 267 | ***Ripk3*** | 3.2E-04 | 3.19 | Rattus norvegicus receptor-interacting serine-threonine kinase 3 (Ripk3), mRNA [NM_139342] |
| 268 | ***Ptplad1*** | 4.2E-02 | 3.18 | Rattus norvegicus protein tyrosine phosphatase-like A domain containing 1 (Ptplad1), mRNA [NM_001106831] |
| 269 | ***Pttg1ip*** | 3.7E-02 | 3.18 | Rattus norvegicus pituitary tumor-transforming 1 interacting protein (Pttg1ip), mRNA [NM_001013238] |
| 270 | ***Uap1*** | 1.8E-03 | 3.18 | Rattus norvegicus UDP-N-acteylglucosamine pyrophosphorylase 1 (Uap1), mRNA [NM_001191930] |
| 271 | ***Unc93b1*** | 1.8E-04 | 3.18 | Rattus norvegicus unc-93 homolog B1 (C. elegans) (Unc93b1), mRNA [NM_001108513] |
| 272 | ***Mcpt8*** | 2.4E-04 | 3.18 | Rattus norvegicus mast cell protease 8 (Mcpt8), mRNA [NM_021598] |
| 273 | ***Npc1*** | 1.9E-02 | 3.17 | Rattus norvegicus Niemann-Pick disease, type C1 (Npc1), mRNA [NM_153624] |
| 274 | ***Rilpl2*** | 1.5E-02 | 3.16 | Rattus norvegicus Rab interacting lysosomal protein-like 2 (Rilpl2), mRNA [NM_001004205] |
| 275 | ***Scp2*** | 6.9E-03 | 3.16 | Rattus norvegicus sterol carrier protein 2 (Scp2), mRNA [NM_138508] |
| 276 | ***RGD1566006*** | 6.1E-04 | 3.15 | PREDICTED: Rattus norvegicus paired immunoglobulin-like type 2 receptor beta-like (RGD1566006), mRNA [XM_006249195] |
| 277 | ***Hsp90b1*** | 7.1E-03 | 3.15 | Rattus norvegicus heat shock protein 90, beta, member 1 (Hsp90b1), mRNA [NM_001012197] |
| 278 | ***St6gal1*** | 1.2E-04 | 3.15 | Rattus norvegicus ST6 beta-galactosamide alpha-2,6-sialyltranferase 1 (St6gal1), transcript variant 2, mRNA [NM_147205] |
| 279 | ***Dnajc22*** | 9.1E-03 | 3.14 | Rattus norvegicus DnaJ (Hsp40) homolog, subfamily C, member 22 (Dnajc22), mRNA [NM_001014204] |
| 280 | ***Comtd1*** | 2.2E-02 | 3.13 | Rattus norvegicus catechol-O-methyltransferase domain containing 1 (Comtd1), mRNA [NM_001107249] |
| 281 | ***Ran*** | 8.6E-03 | 3.13 | Rattus norvegicus RAN, member RAS oncogene family (Ran), mRNA [NM_053439] |
| 282 | ***LOC681309*** | 9.4E-03 | 3.12 | PREDICTED: Rattus norvegicus thrombospondin 3 (LOC681309), mRNA [XM_003749320] |
| 283 | ***Atp5f1*** | 6.3E-03 | 3.12 | Rattus norvegicus ATP synthase, H+ transporting, mitochondrial Fo complex, subunit B1 (Atp5f1), mRNA [NM_134365] |
| 284 | ***Laptm5*** | 7.6E-03 | 3.11 | Rattus norvegicus lysosomal protein transmembrane 5 (Laptm5), mRNA [NM_053538] |
| 285 | ***LOC688932*** | 3.2E-04 | 3.10 | PREDICTED: Rattus norvegicus heat shock cognate 71 kDa protein-like (LOC688932), misc_RNA [XR_085946] |
| 286 | ***Aldoart2*** | 3.7E-03 | 3.09 | Rattus norvegicus aldolase 1 A retrogene 2 (Aldoart2), mRNA [NM_001013943] |
| 287 | ***Enpp2*** | 6.2E-03 | 3.09 | Rattus norvegicus ectonucleotide pyrophosphatase/phosphodiesterase 2 (Enpp2), mRNA [NM_057104] |
| 288 | ***Gcs1*** | 4.0E-02 | 3.08 | Rattus norvegicus glucosidase 1 (Gcs1), mRNA [NM_031749] |
| 289 | ***Npap60*** | 2.6E-02 | 3.07 | Rattus norvegicus nuclear pore associated protein (Npap60), mRNA [NM_012991] |
| 290 | ***Lman2*** | 2.7E-02 | 3.07 | Rattus norvegicus lectin, mannose-binding 2 (Lman2), mRNA [NM_001115024] |
| 291 | ***Sec11c*** | 1.3E-03 | 3.06 | Rattus norvegicus SEC11 homolog C (S. cerevisiae) (Sec11c), mRNA [NM_153628] |
| 292 | ***Tmed10*** | 1.9E-02 | 3.06 | Rattus norvegicus transmembrane emp24-like trafficking protein 10 (yeast) (Tmed10), mRNA [NM_053467] |
| 293 | ***Clic1*** | 3.0E-03 | 3.06 | Rattus norvegicus chloride intracellular channel 1 (Clic1), mRNA [NM_001002807] |
| 294 | ***RT1-Db1*** | 4.7E-03 | 3.05 | Rattus norvegicus RT1 class II, locus Db1 (RT1-Db1), mRNA [NM_001008884] |
| 295 | ***Tjp3*** | 1.8E-03 | 3.05 | Rattus norvegicus tight junction protein 3 (Tjp3), mRNA [NM_001108073] |
| 296 | ***Ptcd3*** | 1.5E-02 | 3.02 | Rattus norvegicus Pentatricopeptide repeat domain 3 (Ptcd3), mRNA [NM_001134718] |
| 297 | ***LOC619574*** | 3.1E-02 | 3.02 | Rattus norvegicus hypothetical protein LOC619574 (LOC619574), mRNA [NM_001034959] |
| 298 | ***Chst1*** | 1.9E-03 | 3.02 | Rattus norvegicus carbohydrate (keratan sulfate Gal-6) sulfotransferase 1 (Chst1), mRNA [NM_001011955] |
| 299 | ***Elf3*** | 3.3E-03 | 3.01 | Rattus norvegicus E74-like factor 3 (Elf3), mRNA [NM_001024768] |
| 300 | ***Ttc9*** | 7.0E-03 | 3.01 | Rattus norvegicus tetratricopeptide repeat domain 9 (Ttc9), mRNA [NM_001134731] |
| 301 | ***Sh3bgrl3*** | 9.8E-03 | 2.98 | Rattus norvegicus SH3 domain binding glutamic acid-rich protein-like 3 (Sh3bgrl3), mRNA [NM_001106688] |
| 302 | ***RGD1307752*** | 2.3E-02 | 2.98 | Rattus norvegicus similar to RIKEN cDNA 1110008F13 (RGD1307752), mRNA [NM_001013922] |
| 303 | ***Crip1*** | 6.2E-03 | 2.97 | Rattus norvegicus cysteine-rich protein 1 (intestinal) (Crip1), transcript variant 2, mRNA [NM_001134933] |
| 304 | ***Slc6a6*** | 1.4E-02 | 2.97 | Rattus norvegicus solute carrier family 6 (neurotransmitter transporter), member 6 (Slc6a6), mRNA [NM_017206] |
| 305 | ***St14*** | 3.7E-02 | 2.97 | Rattus norvegicus suppression of tumorigenicity 14 (colon carcinoma) (St14), mRNA [NM_053635] |
| 306 | ***Sulf2*** | 2.5E-02 | 2.96 | Rattus norvegicus sulfatase 2 (Sulf2), mRNA [NM_001034927] |
| 307 | ***Evi2a*** | 1.2E-02 | 2.95 | Rattus norvegicus ecotropic viral integration site 2A (Evi2a), mRNA [NM_001044287] |
| 308 | ***Pkm*** | 2.8E-03 | 2.95 | Rattus norvegicus pyruvate kinase, muscle (Pkm), mRNA [NM_053297] |
| 309 | ***Rbm38*** | 1.2E-05 | 2.94 | Rattus norvegicus RNA binding motif protein 38 (Rbm38), mRNA [NM_001108965] |
| 310 | ***Fkbp4*** | 6.4E-04 | 2.93 | Rattus norvegicus FK506 binding protein 4 (Fkbp4), mRNA [NM_001191863] |
| 311 | ***Lamp3*** | 2.3E-02 | 2.93 | Rattus norvegicus lysosomal-associated membrane protein 3 (Lamp3), mRNA [NM_001012015] |
| 312 | ***Creld1*** | 2.6E-02 | 2.93 | Rattus norvegicus cysteine-rich with EGF-like domains 1 (Creld1), mRNA [NM_001024783] |
| 313 | ***Rnf149*** | 6.9E-03 | 2.92 | PREDICTED: Rattus norvegicus ring finger protein 149 (Rnf149), mRNA [XM_343561] |
| 314 | ***Ahsa1*** | 1.5E-04 | 2.92 | Rattus norvegicus AHA1, activator of heat shock 90kDa protein ATPase homolog 1 (yeast) (Ahsa1), mRNA [NM_001115034] |
| 315 | ***Atp11a*** | 4.7E-02 | 2.92 | Rattus norvegicus ATPase, class VI, type 11A (Atp11a), mRNA [NM_001107324] |
| 316 | ***Nagk*** | 1.3E-02 | 2.92 | Rattus norvegicus N-acetylglucosamine kinase (Nagk), mRNA [NM_001037768] |
| 317 | ***Fxyd3*** | 4.2E-03 | 2.91 | Rattus norvegicus FXYD domain-containing ion transport regulator 3 (Fxyd3), mRNA [NM_172317] |
| 318 | ***Ddit3*** | 2.1E-03 | 2.91 | Rattus norvegicus DNA-damage inducible transcript 3 (Ddit3), transcript variant 1, mRNA [NM_001109986] |
| 319 | ***Arpc1b*** | 2.2E-04 | 2.90 | Rattus norvegicus actin related protein 2/3 complex, subunit 1B (Arpc1b), mRNA [NM_019289] |
| 320 | ***Ctu2*** | 1.4E-02 | 2.90 | Rattus norvegicus cytosolic thiouridylase subunit 2 homolog (S. pombe) (Ctu2), mRNA [NM_001037094] |
| 321 | ***Atp6v0d1*** | 2.3E-03 | 2.89 | Rattus norvegicus ATPase, H+ transporting, lysosomal V0 subunit D1 (Atp6v0d1), mRNA [NM_001011927] |
| 322 | ***Napsa*** | 3.2E-02 | 2.89 | Rattus norvegicus napsin A aspartic peptidase (Napsa), mRNA [NM_031670] |
| 323 | ***Nme2*** | 2.1E-03 | 2.88 | Rattus norvegicus NME/NM23 nucleoside diphosphate kinase 2 (Nme2), mRNA [NM_031833] |
| 324 | ***Khk*** | 4.4E-04 | 2.88 | Rattus norvegicus ketohexokinase (Khk), mRNA [NM_031855] |
| 325 | ***C1r*** | 2.8E-03 | 2.87 | Rattus norvegicus complement component 1, r subcomponent (C1r), mRNA [NM_001134555] |
| 326 | ***Tmbim4*** | 7.7E-03 | 2.87 | Rattus norvegicus transmembrane BAX inhibitor motif containing 4 (Tmbim4), mRNA [NM_199116] |
| 327 | ***Lrrc59*** | 1.2E-02 | 2.87 | Rattus norvegicus leucine rich repeat containing 59 (Lrrc59), mRNA [NM_001008280] |
| 328 | ***Slc22a18*** | 1.8E-02 | 2.87 | Rattus norvegicus solute carrier family 22, member 18 (Slc22a18), mRNA [NM_001004260] |
| 329 | ***Ppic*** | 4.0E-02 | 2.86 | Rattus norvegicus peptidylprolyl isomerase C (Ppic), mRNA [NM_001004215] |
| 330 | ***Mafg*** | 2.7E-03 | 2.86 | Rattus norvegicus v-maf avian musculoaponeurotic fibrosarcoma oncogene homolog G (Mafg), mRNA [NM_022386] |
| 331 | ***Atp6v1d*** | 4.8E-05 | 2.86 | Rattus norvegicus ATPase, H+ transporting, lysosomal V1 subunit D (Atp6v1d), mRNA [NM_199386] |
| 332 | ***Stat5a*** | 2.4E-02 | 2.86 | Rattus norvegicus signal transducer and activator of transcription 5A (Stat5a), mRNA [NM_017064] |
| 333 | ***Lrp1*** | 2.1E-03 | 2.86 | Rattus norvegicus low density lipoprotein receptor-related protein 1 (Lrp1), mRNA [NM_001130490] |
| 334 | ***Slc26a6*** | 3.1E-03 | 2.85 | Rattus norvegicus solute carrier family 26 (anion exchanger), member 6 (Slc26a6), mRNA [NM_001143817] |
| 335 | ***Nceh1*** | 1.2E-02 | 2.85 | Rattus norvegicus neutral cholesterol ester hydrolase 1 (Nceh1), mRNA [NM_001127524] |
| 336 | ***Flcn*** | 1.9E-02 | 2.85 | Rattus norvegicus folliculin (Flcn), mRNA [NM_199390] |
| 337 | ***Lasp1*** | 2.4E-02 | 2.84 | Rattus norvegicus LIM and SH3 protein 1 (Lasp1), mRNA [NM_032613] |
| 338 | ***Slc22a23*** | 1.5E-02 | 2.84 | Rattus norvegicus solute carrier family 22, member 23 (Slc22a23), mRNA [NM_022624] |
| 339 | ***Pdlim7*** | 4.6E-03 | 2.84 | Rattus norvegicus PDZ and LIM domain 7 (Pdlim7), mRNA [NM_173125] |
| 340 | ***Mri1*** | 2.9E-02 | 2.84 | Rattus norvegicus methylthioribose-1-phosphate isomerase 1 (Mri1), mRNA [NM_001010947] |
| 341 | ***Tnfaip8l2*** | 1.8E-03 | 2.83 | Rattus norvegicus tumor necrosis factor, alpha-induced protein 8-like 2 (Tnfaip8l2), mRNA [NM_001014039] |
| 342 | ***RGD1305464*** | 4.9E-03 | 2.83 | Rattus norvegicus similar to human chromosome 15 open reading frame 39 (RGD1305464), mRNA [NM_001025011] |
| 343 | ***Letm1*** | 4.4E-03 | 2.83 | Rattus norvegicus leucine zipper-EF-hand containing transmembrane protein 1 (Letm1), mRNA [NM_001005884] |
| 344 | ***Clint1*** | 8.7E-03 | 2.82 | PREDICTED: Rattus norvegicus clathrin interactor 1 (Clint1), transcript variant X1, mRNA [XM_006246149] |
| 345 | ***Fdps*** | 2.0E-02 | 2.82 | Rattus norvegicus farnesyl diphosphate synthase (Fdps), mRNA [NM_031840] |
| 346 | ***Fcgr1a*** | 1.0E-02 | 2.82 | Rattus norvegicus Fc fragment of IgG, high affinity Ia, receptor (CD64) (Fcgr1a), mRNA [NM_001100836] |
| 347 | ***Arl4c*** | 4.0E-04 | 2.82 | PREDICTED: Rattus norvegicus ADP-ribosylation factor-like 4C (Arl4c), transcript variant 1, mRNA [XM_003754546] |
| 348 | ***Rt1.aa*** | 6.5E-03 | 2.82 | Rattus norvegicus MHC class I RT1.Aa alpha-chain (Rt1.aa), mRNA [NM_001134701] |
| 349 | ***Psmb8*** | 5.1E-03 | 2.82 | Rattus norvegicus proteasome (prosome, macropain) subunit, beta type, 8 (Psmb8), mRNA [NM_080767] |
| 350 | ***Eva1c*** | 6.9E-04 | 2.81 | PREDICTED: Rattus norvegicus eva-1 homolog C (Eva1c), transcript variant X1, mRNA [XM_001073261] |
| 351 | ***Blvrb*** | 8.8E-05 | 2.80 | Rattus norvegicus biliverdin reductase B (flavin reductase (NADPH)) (Blvrb), mRNA [NM_001106236] |
| 352 | ***Mien1*** | 1.9E-03 | 2.80 | Rattus norvegicus migration and invasion enhancer 1 (Mien1), mRNA [NM_001108296] |
| 353 | ***Ccl6*** | 2.1E-02 | 2.80 | Rattus norvegicus chemokine (C-C motif) ligand 6 (Ccl6), mRNA [NM_001004202] |
| 354 | ***Cst3*** | 2.5E-02 | 2.80 | Rattus norvegicus cystatin C (Cst3), mRNA [NM_012837] |
| 355 | ***Pycard*** | 3.1E-03 | 2.80 | Rattus norvegicus PYD and CARD domain containing (Pycard), mRNA [NM_172322] |
| 356 | ***St3gal4*** | 6.7E-03 | 2.80 | Rattus norvegicus ST3 beta-galactoside alpha-2,3-sialyltransferase 4 (St3gal4), mRNA [NM_203337] |
| 357 | ***Atox1*** | 1.1E-02 | 2.79 | Rattus norvegicus antioxidant 1 copper chaperone (Atox1), mRNA [NM_053359] |
| 358 | ***Tmsb10*** | 1.2E-02 | 2.79 | Rattus norvegicus thymosin, beta 10 (Tmsb10), mRNA [NM_021261] |
| 359 | ***Il18*** | 1.0E-02 | 2.79 | Rattus norvegicus interleukin 18 (Il18), mRNA [NM_019165] |
| 360 | ***Nupr1*** | 9.5E-04 | 2.79 | Rattus norvegicus nuclear protein, transcriptional regulator, 1 (Nupr1), mRNA [NM_053611] |
| 361 | ***Rtn3*** | 1.3E-02 | 2.78 | Rattus norvegicus reticulon 3 (Rtn3), transcript variant 2, mRNA [NM_001009953] |
| 362 | ***Cacybp*** | 1.1E-04 | 2.78 | Rattus norvegicus calcyclin binding protein (Cacybp), mRNA [NM_001004208] |
| 363 | ***RT1-DMb*** | 7.0E-04 | 2.77 | Rattus norvegicus RT1 class II, locus DMb (RT1-DMb), mRNA [NM_198740] |
| 364 | ***Eno1*** | 9.8E-04 | 2.77 | Rattus norvegicus enolase 1, (alpha) (Eno1), transcript variant 2, mRNA [NM_001109908] |
| 365 | ***Ssr1*** | 1.5E-02 | 2.77 | Rattus norvegicus signal sequence receptor, alpha (Ssr1), mRNA [NM_001008891] |
| 366 | ***Hsd11b1*** | 4.6E-02 | 2.77 | Rattus norvegicus hydroxysteroid 11-beta dehydrogenase 1 (Hsd11b1), mRNA [NM_017080] |
| 367 | ***Arl8b*** | 2.9E-02 | 2.76 | Rattus norvegicus ADP-ribosylation factor-like 8B (Arl8b), mRNA [NM_001024332] |
| 368 | ***Rasal3*** | 3.0E-02 | 2.76 | Rattus norvegicus RAS protein activator like 3 (Rasal3), mRNA [NM_001134562] |
| 369 | ***Orai3*** | 2.1E-02 | 2.75 | Rattus norvegicus ORAI calcium release-activated calcium modulator 3 (Orai3), mRNA [NM_001014024] |
| 370 | ***Sh3bp2*** | 2.1E-02 | 2.74 | Rattus norvegicus SH3-domain binding protein 2 (Sh3bp2), mRNA [NM_001100684] |
| 371 | ***Rpn2*** | 1.2E-02 | 2.74 | Rattus norvegicus ribophorin II (Rpn2), mRNA [NM_031698] |
| 372 | ***Ifitm2*** | 2.1E-02 | 2.74 | Rattus norvegicus interferon induced transmembrane protein 2 (Ifitm2), mRNA [NM_030833] |
| 373 | ***Grin2c*** | 2.1E-04 | 2.73 | Rattus norvegicus glutamate receptor, ionotropic, N-methyl D-aspartate 2C (Grin2c), mRNA [NM_012575] |
| 374 | ***C1qbp*** | 2.4E-02 | 2.73 | Rattus norvegicus complement component 1, q subcomponent binding protein (C1qbp), mRNA [NM_019259] |
| 375 | ***Ap2s1*** | 4.9E-05 | 2.73 | Rattus norvegicus adaptor-related protein complex 2, sigma 1 subunit (Ap2s1), mRNA [NM_022952] |
| 376 | ***Aldh7a1*** | 4.1E-02 | 2.73 | Rattus norvegicus aldehyde dehydrogenase 7 family, member A1 (Aldh7a1), mRNA [NM_001271105] |
| 377 | ***Abcc1*** | 1.2E-02 | 2.72 | ATP-binding cassette, subfamily C (CFTR/MRP), member 1 (Abcc1), mRNA [Source:RefSeq mRNA;Acc:NM_022281] [ENSRNOT00000041358] |
| 378 | ***Eif4ebp1*** | 6.3E-04 | 2.72 | Rattus norvegicus eukaryotic translation initiation factor 4E binding protein 1 (Eif4ebp1), mRNA [NM_053857] |
| 379 | ***Cib1*** | 9.7E-03 | 2.72 | Rattus norvegicus calcium and integrin binding 1 (calmyrin) (Cib1), mRNA [NM_031145] |
| 380 | ***Efemp2*** | 1.4E-03 | 2.72 | Rattus norvegicus EGF-containing fibulin-like extracellular matrix protein 2 (Efemp2), transcript variant 1, mRNA [NM_001277341] |
| 381 | ***Dbnl*** | 1.8E-02 | 2.72 | Rattus norvegicus drebrin-like (Dbnl), transcript variant 1, mRNA [NM_001277211] |
| 382 | ***Dynlt1*** | 5.8E-03 | 2.71 | Rattus norvegicus dynein light chain Tctex-type 1 (Dynlt1), mRNA [NM_031318] |
| 383 | ***Ccdc28a*** | 1.0E-03 | 2.71 | Rattus norvegicus coiled-coil domain containing 28A (Ccdc28a), mRNA [NM_001037789] |
| 384 | ***Ppib*** | 1.3E-02 | 2.71 | Rattus norvegicus peptidylprolyl isomerase B (Ppib), mRNA [NM_022536] |
| 385 | ***Grb7*** | 2.0E-03 | 2.70 | Rattus norvegicus growth factor receptor bound protein 7 (Grb7), mRNA [NM_053403] |
| 386 | ***Il10rb*** | 9.7E-03 | 2.70 | Rattus norvegicus interleukin 10 receptor, beta (Il10rb), mRNA [NM_001107111] |
| 387 | ***Hspa8*** | 2.0E-04 | 2.70 | Rattus norvegicus heat shock 70kDa protein 8 (Hspa8), mRNA [NM_024351] |
| 388 | ***Tmem256*** | 4.8E-04 | 2.70 | Rattus norvegicus transmembrane protein 256 (Tmem256), mRNA [NM_001170549] |
| 389 | ***Fxyd2*** | 4.9E-04 | 2.70 | Rattus norvegicus FXYD domain-containing ion transport regulator 2 (Fxyd2), transcript variant a, mRNA [NM_145717] |
| 390 | ***Lcp1*** | 1.4E-02 | 2.70 | Rattus norvegicus lymphocyte cytosolic protein 1 (Lcp1), mRNA [NM_001012044] |
| 391 | ***Zfp46*** | 1.2E-02 | 2.70 | Rattus norvegicus zinc finger protein 46 (Zfp46), mRNA [NM_001106691] |
| 392 | ***Fam49b*** | 1.7E-02 | 2.69 | Rattus norvegicus family with sequence similarity 49, member B (Fam49b), mRNA [NM_001126267] |
| 393 | ***Vcp*** | 1.5E-02 | 2.68 | Rattus norvegicus valosin-containing protein (Vcp), mRNA [NM_053864] |
| 394 | ***Ptdss1*** | 2.0E-02 | 2.67 | Rattus norvegicus phosphatidylserine synthase 1 (Ptdss1), mRNA [NM_001012113] |
| 395 | ***Taldo1*** | 2.1E-02 | 2.67 | Rattus norvegicus transaldolase 1 (Taldo1), mRNA [NM_031811] |
| 396 | ***Pet100*** | 5.6E-03 | 2.66 | Rattus norvegicus PET100 homolog (S. cerevisiae) (Pet100), mRNA [NM_001195245] |
| 397 | ***Gls*** | 4.4E-05 | 2.66 | Rattus norvegicus glutaminase (Gls), nuclear gene encoding mitochondrial protein, transcript variant 1, mRNA [NM_012569] |
| 398 | ***Med10*** | 2.3E-02 | 2.65 | Rattus norvegicus mediator complex subunit 10 (Med10), mRNA [NM_001106097] |
| 399 | ***Asl*** | 4.3E-02 | 2.65 | Rattus norvegicus argininosuccinate lyase (Asl), mRNA [NM_021577] |
| 400 | ***Scpep1*** | 3.0E-02 | 2.64 | Rattus norvegicus serine carboxypeptidase 1 (Scpep1), mRNA [NM_133383] |
| 401 | ***Tmem33*** | 1.4E-03 | 2.64 | Rattus norvegicus transmembrane protein 33 (Tmem33), transcript variant 1, mRNA [NM_021671] |
| 402 | ***Prkar1b*** | 2.8E-04 | 2.64 | Rattus norvegicus protein kinase, cAMP dependent regulatory, type I, beta (Prkar1b), mRNA [NM_001033679] |
| 403 | ***LOC501282*** | 2.5E-02 | 2.64 | DNA segment, Chr 17, Wayne State University 104, expressed [Source:MGI Symbol;Acc:MGI:2156020] [ENSRNOT00000072870] |
| 404 | ***Cp*** | 1.0E-02 | 2.64 | Rattus norvegicus ceruloplasmin (ferroxidase) (Cp), transcript variant 1, mRNA [NM_012532] |
| 405 | ***Hspa8*** | 4.5E-06 | 2.63 | Rattus norvegicus heat shock 70kDa protein 8 (Hspa8), mRNA [NM_024351] |
| 406 | ***Hsp90ab1*** | 1.3E-03 | 2.63 | Rattus norvegicus heat shock protein 90 alpha (cytosolic), class B member 1 (Hsp90ab1), mRNA [NM_001004082] |
| 407 | ***Erp29*** | 3.8E-02 | 2.63 | Rattus norvegicus endoplasmic reticulum protein 29 (Erp29), mRNA [NM_053961] |
| 408 | ***Tekt2*** | 3.2E-03 | 2.63 | Rattus norvegicus tektin 2 (testicular) (Tekt2), mRNA [NM_001011977] |
| 409 | ***Mars*** | 1.7E-02 | 2.63 | Rattus norvegicus methionyl-tRNA synthetase (Mars), mRNA [NM_001127659] |
| 410 | ***Car8*** | 2.6E-02 | 2.62 | Rattus norvegicus carbonic anhydrase 8 (Car8), mRNA [NM_001009662] |
| 411 | ***Hsp90ab1*** | 6.4E-04 | 2.62 | Rattus norvegicus heat shock protein 90 alpha (cytosolic), class B member 1 (Hsp90ab1), mRNA [NM_001004082] |
| 412 | ***Xdh*** | 2.7E-02 | 2.62 | Rattus norvegicus xanthine dehydrogenase (Xdh), mRNA [NM_017154] |
| 413 | ***Slc35b1*** | 2.1E-02 | 2.62 | Rattus norvegicus solute carrier family 35, member B1 (Slc35b1), mRNA [NM_199081] |
| 414 | ***Aif1*** | 2.5E-03 | 2.62 | Rattus norvegicus allograft inflammatory factor 1 (Aif1), mRNA [NM_017196] |
| 415 | ***Atp6v1g1*** | 1.4E-02 | 2.62 | Rattus norvegicus ATPase, H transporting, lysosomal V1 subunit G1 (Atp6v1g1), mRNA [NM_001106660] |
| 416 | ***Il1rn*** | 2.1E-02 | 2.62 | Rattus norvegicus interleukin 1 receptor antagonist (Il1rn), mRNA [NM_022194] |
| 417 | ***Aprt*** | 2.5E-02 | 2.61 | Rattus norvegicus adenine phosphoribosyl transferase (Aprt), mRNA [NM_001013061] |
| 418 | ***Aen*** | 1.0E-03 | 2.61 | Rattus norvegicus apoptosis enhancing nuclease (Aen), mRNA [NM_001108487] |
| 419 | ***Glb1*** | 2.7E-05 | 2.61 | Rattus norvegicus galactosidase, beta 1 (Glb1), mRNA [NM_001108192] |
| 420 | ***RT1-EC2*** | 1.5E-02 | 2.61 | Rattus norvegicus RT1 class Ib, locus EC2 (RT1-EC2), mRNA [NM_012645] |
| 421 | ***RGD1560281*** | 2.5E-04 | 2.61 | PREDICTED: Rattus norvegicus paired immunoglobulin-like type 2 receptor beta-like (RGD1560281), mRNA [XM_006221312] |
| 422 | ***Hspa8*** | 3.2E-04 | 2.61 | Rattus norvegicus heat shock 70kDa protein 8 (Hspa8), mRNA [NM_024351] |
| 423 | ***Esrra*** | 4.1E-02 | 2.60 | Rattus norvegicus estrogen related receptor, alpha (Esrra), mRNA [NM_001008511] |
| 424 | ***Arhgdib*** | 1.8E-02 | 2.60 | Rattus norvegicus Rho, GDP dissociation inhibitor (GDI) beta (Arhgdib), mRNA [NM_001009600] |
| 425 | ***Bak1*** | 1.2E-02 | 2.60 | Rattus norvegicus BCL2-antagonist/killer 1 (Bak1), mRNA [NM_053812] |
| 426 | ***Ssu72*** | 3.0E-02 | 2.59 | Rattus norvegicus SSU72 RNA polymerase II CTD phosphatase homolog (S. cerevisiae) (Ssu72), mRNA [NM_001025657] |
| 427 | ***Rgs10*** | 1.4E-04 | 2.59 | Rattus norvegicus regulator of G-protein signaling 10 (Rgs10), mRNA [NM_019337] |
| 428 | ***Rmdn2*** | 5.0E-03 | 2.59 | Rattus norvegicus regulator of microtubule dynamics 2 (Rmdn2), mRNA [NM_001037200] |
| 429 | ***Rps6ka1*** | 1.5E-02 | 2.59 | Rattus norvegicus ribosomal protein S6 kinase polypeptide 1 (Rps6ka1), mRNA [NM_031107] |
| 430 | ***Rab31*** | 4.8E-02 | 2.58 | Rattus norvegicus RAB31, member RAS oncogene family (Rab31), mRNA [NM_145094] |
| 431 | ***Lrp2*** | 1.4E-02 | 2.58 | Rattus norvegicus low density lipoprotein receptor-related protein 2 (Lrp2), mRNA [NM_030827] |
| 432 | ***Myo1g*** | 1.1E-02 | 2.58 | Rattus norvegicus myosin IG (Myo1g), mRNA [NM_001134843] |
| 433 | ***Smim4*** | 1.5E-02 | 2.58 | LOC361111 (Predicted), isoform CRA_a; Protein Snhg8 [Source:UniProtKB/TrEMBL;Acc:D4A8X7] [ENSRNOT00000061204] |
| 434 | ***Parp1*** | 2.2E-02 | 2.58 | Rattus norvegicus poly (ADP-ribose) polymerase 1 (Parp1), mRNA [NM_013063] |
| 435 | ***Mrpl17*** | 1.6E-02 | 2.57 | Rattus norvegicus mitochondrial ribosomal protein L17 (Mrpl17), mRNA [NM_133539] |
| 436 | ***Lhpp*** | 2.9E-02 | 2.57 | Rattus norvegicus phospholysine phosphohistidine inorganic pyrophosphate phosphatase (Lhpp), mRNA [NM_001009706] |
| 437 | ***Ikbke*** | 3.6E-02 | 2.57 | Rattus norvegicus inhibitor of kappa light polypeptide gene enhancer in B-cells, kinase epsilon (Ikbke), mRNA [NM_001108854] |
| 438 | ***Ccl6*** | 4.0E-02 | 2.56 | chemokine (C-C motif) ligand 6 (Ccl6), mRNA [Source:RefSeq mRNA;Acc:NM_001004202] [ENSRNOT00000045867] |
| 439 | ***Chchd6*** | 2.8E-03 | 2.56 | Rattus norvegicus coiled-coil-helix-coiled-coil-helix domain containing 6 (Chchd6), mRNA [NM_001106608] |
| 440 | ***Leprotl1*** | 1.6E-02 | 2.56 | Rattus norvegicus leptin receptor overlapping transcript-like 1 (Leprotl1), mRNA [NM_001013188] |
| 441 | ***Spdef*** | 2.0E-02 | 2.55 | Rattus norvegicus SAM pointed domain containing ets transcription factor (Spdef), mRNA [NM_001109530] |
| 442 | ***Mrpl18*** | 1.2E-02 | 2.55 | Rattus norvegicus mitochondrial ribosomal protein L18 (Mrpl18), mRNA [NM_001106205] |
| 443 | ***Acsl5*** | 3.2E-02 | 2.55 | Rattus norvegicus acyl-CoA synthetase long-chain family member 5 (Acsl5), mRNA [NM_053607] |
| 444 | ***Nhp2*** | 6.6E-03 | 2.55 | Rattus norvegicus NHP2 ribonucleoprotein (Nhp2), mRNA [NM_001105779] |
| 445 | ***Cox5b*** | 9.6E-03 | 2.54 | Rattus norvegicus cytochrome c oxidase subunit Vb (Cox5b), mRNA [NM_053586] |
| 446 | ***Mcoln1*** | 6.3E-03 | 2.54 | Rattus norvegicus mucolipin 1 (Mcoln1), mRNA [NM_001105903] |
| 447 | ***RT1-A1*** | 1.2E-02 | 2.54 | Rattus norvegicus RT1 class Ia, locus A1 (RT1-A1), mRNA [NM_001008827] |
| 448 | ***Gadd45gip1*** | 3.4E-02 | 2.54 | Rattus norvegicus growth arrest and DNA-damage-inducible, gamma interacting protein 1 (Gadd45gip1), mRNA [NM_001100504] |
| 449 | ***Cyp51*** | 2.7E-03 | 2.54 | Rattus norvegicus cytochrome P450, family 51 (Cyp51), mRNA [NM_012941] |
| 450 | ***Atp6v1a*** | 2.9E-03 | 2.54 | Rattus norvegicus ATPase, H+ transporting, lysosomal V1 subunit A (Atp6v1a), mRNA [NM_001108318] |
| 451 | ***Atic*** | 1.3E-02 | 2.53 | Rattus norvegicus 5-aminoimidazole-4-carboxamide ribonucleotide formyltransferase/IMP cyclohydrolase (Atic), mRNA [NM_031014] |
| 452 | ***Spcs1*** | 8.2E-03 | 2.53 | Rattus norvegicus signal peptidase complex subunit 1 homolog (S. cerevisiae) (Spcs1), mRNA [NM_001131006] |
| 453 | ***Scarb1*** | 1.9E-02 | 2.52 | Rattus norvegicus scavenger receptor class B, member 1 (Scarb1), mRNA [NM_031541] |
| 454 | ***Hsp90ab1*** | 1.2E-03 | 2.52 | heat shock protein 90 alpha (cytosolic), class B member 1 (Hsp90ab1), mRNA [Source:RefSeq mRNA;Acc:NM_001004082] [ENSRNOT00000026920] |
| 455 | ***H2afz*** | 2.8E-03 | 2.51 | Rattus norvegicus H2A histone family, member Z (H2afz), mRNA [NM_022674] |
| 456 | ***Sbno2*** | 2.2E-03 | 2.51 | Rattus norvegicus strawberry notch homolog 2 (Drosophila) (Sbno2), mRNA [NM_001108068] |
| 457 | ***Kdelr1*** | 1.9E-02 | 2.50 | Rattus norvegicus KDEL (Lys-Asp-Glu-Leu) endoplasmic reticulum protein retention receptor 1 (Kdelr1), mRNA [NM_001017385] |
| 458 | ***Tspo*** | 2.9E-02 | 2.50 | Rattus norvegicus translocator protein (Tspo), mRNA [NM_012515] |
| 459 | ***Wfdc2*** | 3.6E-02 | 2.50 | Rattus norvegicus WAP four-disulfide core domain 2 (Wfdc2), mRNA [NM_173109] |
| 460 | ***Msrb1*** | 1.5E-04 | 2.50 | Rattus norvegicus methionine sulfoxide reductase B1 (Msrb1), mRNA [NM_001044285] |
| 461 | ***Tex264*** | 2.2E-02 | 2.50 | Rattus norvegicus testis expressed 264 (Tex264), mRNA [NM_001007665] |
| 462 | ***Atp6v1c1*** | 8.6E-03 | 2.49 | Rattus norvegicus ATPase, H+ transporting, lysosomal V1 subunit C1 (Atp6v1c1), mRNA [NM_001011992] |
| 463 | ***Tes*** | 3.0E-03 | 2.49 | Rattus norvegicus testis derived transcript (Tes), mRNA [NM_001039344] |
| 464 | ***Gpx1*** | 1.0E-02 | 2.49 | Rattus norvegicus glutathione peroxidase 1 (Gpx1), mRNA [NM_030826] |
| 465 | ***Ifitm3*** | 2.8E-02 | 2.49 | Rattus norvegicus interferon induced transmembrane protein 3 (Ifitm3), mRNA [NM_001136124] |
| 466 | ***Usp34*** | 2.6E-02 | 2.49 | Rattus norvegicus ubiquitin specific peptidase 34 (Usp34), mRNA [NM_001271196] |
| 467 | ***Atp6v0a1*** | 4.2E-02 | 2.49 | Rattus norvegicus ATPase, H+ transporting, lysosomal V0 subunit A1 (Atp6v0a1), mRNA [NM_031604] |
| 468 | ***Fam46a*** | 3.0E-04 | 2.49 | Rattus norvegicus family with sequence similarity 46, member A (Fam46a), mRNA [NM_001106844] |
| 469 | ***Plod3*** | 2.3E-02 | 2.49 | Rattus norvegicus procollagen-lysine, 2-oxoglutarate 5-dioxygenase 3 (Plod3), mRNA [NM_178101] |
| 470 | ***Iah1*** | 3.1E-02 | 2.49 | Rattus norvegicus isoamyl acetate-hydrolyzing esterase 1 homolog (S. cerevisiae) (Iah1), mRNA [NM_001100540] |
| 471 | ***RGD1563348*** | 4.7E-02 | 2.49 | Rattus norvegicus similar to Selenoprotein H (RGD1563348), mRNA [NM_001114939] |
| 472 | ***Galk2*** | 1.6E-02 | 2.48 | Rattus norvegicus galactokinase 2 (Galk2), mRNA [NM_001013919] |
| 473 | ***mrpl24*** | 7.3E-04 | 2.48 | Rattus norvegicus mitochondrial ribosomal protein L24 (mrpl24), mRNA [NM_001007637] |
| 474 | ***Rab27a*** | 1.2E-02 | 2.48 | Rattus norvegicus RAB27A, member RAS oncogene family (Rab27a), mRNA [NM_017317] |
| 475 | ***Nup62*** | 2.4E-02 | 2.48 | Rattus norvegicus nucleoporin 62 (Nup62), mRNA [NM_023098] |
| 476 | ***RGD1564614*** | 7.6E-04 | 2.48 | Rattus norvegicus similar to complement factor H-related protein (RGD1564614), mRNA [NM_001134792] |
| 477 | ***Mvd*** | 2.1E-02 | 2.48 | Rattus norvegicus mevalonate (diphospho) decarboxylase (Mvd), mRNA [NM_031062] |
| 478 | ***Timm13*** | 3.4E-02 | 2.46 | Rattus norvegicus translocase of inner mitochondrial membrane 13 homolog (yeast) (Timm13), mRNA [NM_145781] |
| 479 | ***LOC685186*** | 3.7E-05 | 2.46 | Protein LOC685186 [Source:UniProtKB/TrEMBL;Acc:M0R590] [ENSRNOT00000041328] |
| 480 | ***Snx18*** | 8.9E-03 | 2.46 | Rattus norvegicus sorting nexin 18 (Snx18), mRNA [NM_001107652] |
| 481 | ***Rasa3*** | 1.3E-02 | 2.46 | Rattus norvegicus mRNA for R-ras GTPase activating protein, partial cds. [AB028626] |
| 482 | ***RT1-CE2*** | 4.0E-03 | 2.46 | Rattus norvegicus RT1 class I, locus CE2 (RT1-CE2), mRNA [NM_001008840] |
| 483 | ***Abracl*** | 6.5E-03 | 2.46 | Rattus norvegicus ABRA C-terminal like (Abracl), mRNA [NM_001099647] |
| 484 | ***Pigz*** | 9.0E-05 | 2.45 | Rattus norvegicus phosphatidylinositol glycan anchor biosynthesis, class Z (Pigz), mRNA [NM_001109525] |
| 485 | ***Calm1*** | 2.2E-02 | 2.45 | Rattus norvegicus calmodulin 1 (Calm1), mRNA [NM_031969] |
| 486 | ***Man2a1*** | 4.4E-02 | 2.45 | Rattus norvegicus mannosidase, alpha, class 2A, member 1 (Man2a1), mRNA [NM_012979] |
| 487 | ***Sqstm1*** | 6.9E-03 | 2.45 | Rattus norvegicus sequestosome 1 (Sqstm1), transcript variant 1, mRNA [NM_175843] |
| 488 | ***Tspan4*** | 2.9E-04 | 2.45 | Rattus norvegicus tetraspanin 4 (Tspan4), mRNA [NM_001013070] |
| 489 | ***Gpn1*** | 1.8E-02 | 2.44 | Rattus norvegicus GPN-loop GTPase 1 (Gpn1), mRNA [NM_001127572] |
| 490 | ***Esyt1*** | 3.4E-04 | 2.44 | Rattus norvegicus extended synaptotagmin-like protein 1 (Esyt1), mRNA [NM_017249] |
| 491 | ***Suclg1*** | 4.0E-02 | 2.44 | Rattus norvegicus succinate-CoA ligase, alpha subunit (Suclg1), mRNA [NM_053752] |
| 492 | ***LOC102556514*** | 3.7E-03 | 2.44 | PREDICTED: Rattus norvegicus complement C1s subcomponent-like (LOC102556514), misc_RNA [XR_346300] |
| 493 | ***Pigk*** | 2.2E-02 | 2.43 | Rattus norvegicus phosphatidylinositol glycan anchor biosynthesis, class K (Pigk), mRNA [NM_001011953] |
| 494 | ***Ndufa12*** | 8.3E-03 | 2.43 | Rattus norvegicus NADH dehydrogenase (ubiquinone) 1 alpha subcomplex, 12 (Ndufa12), mRNA [NM_001106781] |
| 495 | ***Sdf4*** | 5.2E-03 | 2.43 | Rattus norvegicus stromal cell derived factor 4 (Sdf4), mRNA [NM_130412] |
| 496 | ***Spint1*** | 5.3E-03 | 2.43 | Rattus norvegicus serine peptidase inhibitor, Kunitz type 1 (Spint1), mRNA [NM_001004265] |
| 497 | ***Pck2*** | 8.8E-04 | 2.43 | Rattus norvegicus phosphoenolpyruvate carboxykinase 2 (mitochondrial) (Pck2), mRNA [NM_001108377] |
| 498 | ***Mdh2*** | 2.2E-02 | 2.43 | Rattus norvegicus malate dehydrogenase 2, NAD (mitochondrial) (Mdh2), mRNA [NM_031151] |
| 499 | ***Abcc1*** | 3.4E-03 | 2.42 | Rattus norvegicus ATP-binding cassette, subfamily C (CFTR/MRP), member 1 (Abcc1), mRNA [NM_022281] |
| 500 | ***Dbnl*** | 3.8E-02 | 2.42 | Rattus norvegicus drebrin-like (Dbnl), transcript variant 1, mRNA [NM_001277211] |
| 501 | ***Tbc1d1*** | 1.1E-03 | 2.42 | PREDICTED: Rattus norvegicus TBC1 (tre-2/USP6, BUB2, cdc16) domain family, member 1 (Tbc1d1), transcript variant X5, mRNA [XM_341215] |
| 502 | ***Cnih1*** | 3.1E-02 | 2.42 | Rattus norvegicus cornichon family AMPA receptor auxiliary protein 1 (Cnih1), mRNA [NM_001106029] |
| 503 | ***Romo1*** | 2.7E-02 | 2.41 | Rattus norvegicus reactive oxygen species modulator 1 (Romo1), mRNA [NM_001195490] |
| 504 | ***LOC691603*** | 1.5E-02 | 2.41 | PREDICTED: Rattus norvegicus mast cell protease 8-like (LOC691603), mRNA [XM_003751469] |
| 505 | ***Vat1*** | 1.8E-02 | 2.41 | Rattus norvegicus vesicle amine transport 1 (Vat1), mRNA [NM_001033683] |
| 506 | ***Wdr83os*** | 2.0E-02 | 2.41 | Rattus norvegicus WD repeat domain 83 opposite strand (Wdr83os), mRNA [NM_001105947] |
| 507 | ***Uqcr10*** | 1.4E-02 | 2.40 | Rattus norvegicus ubiquinol-cytochrome c reductase, complex III subunit X (Uqcr10), mRNA [NM_001170465] |
| 508 | ***Ilvbl*** | 2.7E-02 | 2.40 | Rattus norvegicus ilvB (bacterial acetolactate synthase)-like (Ilvbl), mRNA [NM_001108738] |
| 509 | ***Echs1*** | 1.6E-02 | 2.40 | Rattus norvegicus enoyl CoA hydratase, short chain, 1, mitochondrial (Echs1), mRNA [NM_078623] |
| 510 | ***Odc1*** | 3.1E-02 | 2.40 | Rattus norvegicus ornithine decarboxylase 1 (Odc1), mRNA [NM_012615] |
| 511 | ***Hspa8*** | 1.1E-06 | 2.40 | Rattus norvegicus heat shock 70kDa protein 8 (Hspa8), mRNA [NM_024351] |
| 512 | ***Naprt1*** | 2.6E-03 | 2.39 | Rattus norvegicus nicotinate phosphoribosyltransferase domain containing 1 (Naprt1), mRNA [NM_207609] |
| 513 | ***Cnpy2*** | 3.9E-02 | 2.39 | Rattus norvegicus canopy FGF signaling regulator 2 (Cnpy2), mRNA [NM_001077585] |
| 514 | ***Alg14*** | 3.9E-02 | 2.39 | Rattus norvegicus ALG14, UDP-N-acetylglucosaminyltransferase subunit (Alg14), mRNA [NM_001014176] |
| 515 | ***H2afz*** | 3.5E-03 | 2.39 | Rattus norvegicus H2A histone family, member Z (H2afz), mRNA [NM_022674] |
| 516 | ***Ubqln1*** | 5.7E-03 | 2.39 | Rattus norvegicus ubiquilin 1 (Ubqln1), mRNA [NM_053747] |
| 517 | ***Erp44*** | 1.7E-02 | 2.38 | Rattus norvegicus endoplasmic reticulum protein 44 (Erp44), mRNA [NM_001008317] |
| 518 | ***Capza1*** | 2.4E-03 | 2.38 | Rattus norvegicus capping protein (actin filament) muscle Z-line, alpha 1 (Capza1), mRNA [NM_001109625] |
| 519 | ***Scly*** | 2.7E-02 | 2.38 | Rattus norvegicus selenocysteine lyase (Scly), mRNA [NM_001007755] |
| 520 | ***Col9a2*** | 1.3E-02 | 2.38 | Rattus norvegicus collagen, type IX, alpha 2 (Col9a2), mRNA [NM_001108675] |
| 521 | ***Riok3*** | 1.8E-02 | 2.38 | Rattus norvegicus RIO kinase 3 (Riok3), mRNA [NM_001108423] |
| 522 | ***Igsf6*** | 3.5E-02 | 2.38 | Rattus norvegicus immunoglobulin superfamily, member 6 (Igsf6), mRNA [NM_133542] |
| 523 | ***Atp6v1f*** | 2.2E-03 | 2.38 | Rattus norvegicus ATPase, H transporting, lysosomal V1 subunit F (Atp6v1f), mRNA [NM_053884] |
| 524 | ***Slc9a1*** | 9.5E-03 | 2.38 | Rattus norvegicus solute carrier family 9, subfamily A (NHE1, cation proton antiporter 1), member 1 (Slc9a1), mRNA [NM_012652] |
| 525 | ***Slc38a10*** | 2.1E-02 | 2.38 | Protein Slc38a10; Putative uncharacterized protein RGD1306356 [Source:UniProtKB/TrEMBL;Acc:D3ZH43] [ENSRNOT00000006225] |
| 526 | ***Sec61b*** | 4.5E-02 | 2.38 | Rattus norvegicus Sec61 beta subunit (Sec61b), mRNA [NM_001106654] |
| 527 | ***Hspe1*** | 6.4E-04 | 2.37 | Rattus norvegicus heat shock protein 1 (chaperonin 10) (Hspe1), mRNA [NM_012966] |
| 528 | ***Pgk1*** | 1.5E-02 | 2.37 | Rattus norvegicus phosphoglycerate kinase 1 (Pgk1), mRNA [NM_053291] |
| 529 | ***Ndufb5*** | 1.6E-02 | 2.37 | Rattus norvegicus NADH dehydrogenase (ubiquinone) 1 beta subcomplex, 5 (Ndufb5), mRNA [NM_001106426] |
| 530 | ***Set*** | 4.9E-03 | 2.37 | Rattus norvegicus SET nuclear oncogene (Set), mRNA [NM_001012504] |
| 531 | ***Aars*** | 1.6E-02 | 2.37 | Rattus norvegicus alanyl-tRNA synthetase (Aars), mRNA [NM_001100517] |
| 532 | ***Plin2*** | 2.6E-02 | 2.37 | Rattus norvegicus perilipin 2 (Plin2), mRNA [NM_001007144] |
| 533 | ***Tlcd1*** | 3.0E-03 | 2.36 | Rattus norvegicus TLC domain containing 1 (Tlcd1), mRNA [NM_001013858] |
| 534 | ***Slc39a1*** | 4.8E-02 | 2.36 | Rattus norvegicus solute carrier family 39 (zinc transporter), member 1 (Slc39a1), mRNA [NM_001134577] |
| 535 | ***Hspe1*** | 5.4E-04 | 2.36 | Rattus norvegicus heat shock protein 1 (chaperonin 10) (Hspe1), mRNA [NM_012966] |
| 536 | ***Sdhb*** | 2.1E-02 | 2.36 | Rattus norvegicus succinate dehydrogenase complex, subunit B, iron sulfur (Ip) (Sdhb), mRNA [NM_001100539] |
| 537 | ***Calm1*** | 3.4E-02 | 2.36 | Rattus norvegicus calmodulin 1 (Calm1), mRNA [NM_031969] |
| 538 | ***Gabarap*** | 8.8E-03 | 2.35 | Rattus norvegicus GABA(A) receptor-associated protein (Gabarap), mRNA [NM_172036] |
| 539 | ***Wdr1*** | 1.7E-02 | 2.35 | Rattus norvegicus WD repeat domain 1 (Wdr1), mRNA [NM_001014135] |
| 540 | ***Mpc1*** | 2.2E-02 | 2.35 | Rattus norvegicus mitochondrial pyruvate carrier 1 (Mpc1), mRNA [NM_133561] |
| 541 | ***Adam19*** | 3.9E-02 | 2.35 | Rattus norvegicus ADAM metallopeptidase domain 19 (Adam19), mRNA [NM_001160228] |
| 542 | ***LOC503131*** | 1.1E-02 | 2.35 | PREDICTED: Rattus norvegicus glyceraldehyde-3-phosphate dehydrogenase-like (LOC503131), misc RNA [XR_147054] |
| 543 | ***Nubp2*** | 3.2E-02 | 2.34 | Rattus norvegicus nucleotide binding protein 2 (Nubp2), mRNA [NM_001011891] |
| 544 | ***Man2b1*** | 3.9E-02 | 2.34 | Rattus norvegicus mannosidase, alpha, class 2B, member 1 (Man2b1), mRNA [NM_199404] |
| 545 | ***Slirp*** | 8.2E-03 | 2.34 | Rattus norvegicus SRA stem-loop interacting RNA binding protein (Slirp), mRNA [NM_001109507] |
| 546 | ***RGD1560402*** | 1.5E-02 | 2.33 | PREDICTED: Rattus norvegicus phosphoglycerate kinase 1-like (RGD1560402), mRNA [XM_001074373] |
| 547 | ***Rrbp1*** | 2.3E-02 | 2.33 | ribosome binding protein 1 [Source:MGI Symbol;Acc:MGI:1932395] [ENSRNOT00000007888] |
| 548 | ***Tnnt2*** | 1.4E-03 | 2.33 | Rattus norvegicus troponin T type 2 (cardiac) (Tnnt2), mRNA [NM_012676] |
| 549 | ***Tcf19*** | 4.9E-03 | 2.32 | Rattus norvegicus transcription factor 19 (Tcf19), mRNA [NM_213561] |
| 550 | ***Chchd2*** | 1.8E-02 | 2.32 | Rattus norvegicus coiled-coil-helix-coiled-coil-helix domain containing 2 (Chchd2), mRNA [NM_001015019] |
| 551 | ***Ogfrl1*** | 2.4E-02 | 2.31 | Rattus norvegicus opioid growth factor receptor-like 1 (Ogfrl1), mRNA [NM_001025708] |
| 552 | ***Cisd1*** | 4.4E-02 | 2.31 | Rattus norvegicus CDGSH iron sulfur domain 1 (Cisd1), mRNA [NM_001106385] |
| 553 | ***Ndufb11*** | 3.9E-02 | 2.30 | Rattus norvegicus NADH dehydrogenase (ubiquinone) 1 beta subcomplex, 11 (Ndufb11), mRNA [NM_001106756] |
| 554 | ***Snd1*** | 1.2E-02 | 2.30 | Rattus norvegicus staphylococcal nuclease and tudor domain containing 1 (Snd1), mRNA [NM_022694] |
| 555 | ***Inhbb*** | 1.7E-02 | 2.30 | Rattus norvegicus inhibin beta-B (Inhbb), mRNA [NM_080771] |
| 556 | ***Slc35e3*** | 2.9E-02 | 2.30 | Rattus norvegicus solute carrier family 35, member E3 (Slc35e3), mRNA [NM_001134687] |
| 557 | ***Txn1*** | 2.1E-02 | 2.29 | Rattus norvegicus thioredoxin 1 (Txn1), mRNA [NM_053800] |
| 558 | ***Fh*** | 1.8E-02 | 2.29 | Rattus norvegicus fumarate hydratase (Fh), mRNA [NM_017005] |
| 559 | ***Cyp2s1*** | 5.4E-03 | 2.29 | Rattus norvegicus cytochrome P450, family 2, subfamily s, polypeptide 1 (Cyp2s1), mRNA [NM_001107495] |
| 560 | ***Clta*** | 2.5E-02 | 2.28 | Rattus norvegicus clathrin, light chain A (Clta), mRNA [NM_031974] |
| 561 | ***Fam195a*** | 2.6E-02 | 2.28 | Rattus norvegicus family with sequence similarity 195, member A (Fam195a), mRNA [NM_001109475] |
| 562 | ***Ifi27l2b*** | 2.4E-02 | 2.28 | Rattus norvegicus interferon, alpha-inducible protein 27 like 2B (Ifi27l2b), mRNA [NM_206846] |
| 563 | ***Psmb5*** | 1.9E-02 | 2.28 | Rattus norvegicus proteasome (prosome, macropain) subunit, beta type 5 (Psmb5), mRNA [NM_001105727] |
| 564 | ***Lig1*** | 4.3E-02 | 2.28 | Rattus norvegicus ligase I, DNA, ATP-dependent (Lig1), mRNA [NM_001024268] |
| 565 | ***Ube2a*** | 4.5E-02 | 2.28 | Rattus norvegicus ubiquitin-conjugating enzyme E2A (Ube2a), mRNA [NM_001013933] |
| 566 | ***Rnase4*** | 4.3E-03 | 2.27 | Rattus norvegicus ribonuclease, RNase A family 4, mRNA (cDNA clone MGC:187485 IMAGE:7372999), complete cds. [BC166436] |
| 567 | ***Mrp63*** | 9.2E-03 | 2.27 | Rattus norvegicus mitochondrial ribosomal protein 63 (Mrp63), mRNA [NM_001109649] |
| 568 | ***LOC100909504*** | 5.2E-05 | 2.27 | PREDICTED: Rattus norvegicus heat shock protein HSP 90-beta-like (LOC100909504), partial mRNA [XM_003752259] |
| 569 | ***Slirp*** | 6.5E-03 | 2.27 | Rattus norvegicus SRA stem-loop interacting RNA binding protein (Slirp), mRNA [NM_001109507] |
| 570 | ***Srm*** | 3.3E-03 | 2.27 | Rattus norvegicus spermidine synthase (Srm), mRNA [NM_053464] |
| 571 | ***Mospd2*** | 3.2E-02 | 2.27 | Rattus norvegicus motile sperm domain containing 2 (Mospd2), mRNA [NM_001134588] |
| 572 | ***Lyplal1*** | 4.8E-02 | 2.27 | Rattus norvegicus lysophospholipase-like 1 (Lyplal1), mRNA [NM_001105986] |
| 573 | ***Nme1*** | 1.1E-02 | 2.27 | Rattus norvegicus NME/NM23 nucleoside diphosphate kinase 1 (Nme1), mRNA [NM_138548] |
| 574 | ***Hdhd2*** | 1.2E-02 | 2.26 | Rattus norvegicus haloacid dehalogenase-like hydrolase domain containing 2 (Hdhd2), mRNA [NM_001014151] |
| 575 | ***Stap2*** | 1.0E-02 | 2.26 | Rattus norvegicus signal transducing adaptor family member 2 (Stap2), mRNA [NM_001025026] |
| 576 | ***Yif1a*** | 2.8E-02 | 2.26 | Rattus norvegicus Yip1 interacting factor homolog A (S. cerevisiae) (Yif1a), mRNA [NM_172017] |
| 577 | ***Abcc1*** | 3.2E-02 | 2.25 | Rattus norvegicus ATP-binding cassette, subfamily C (CFTR/MRP), member 1 (Abcc1), mRNA [NM_022281] |
| 578 | ***Rexo2*** | 2.2E-03 | 2.25 | Rattus norvegicus RNA exonuclease 2 (Rexo2), mRNA [NM_001008326] |
| 579 | ***Prdx2*** | 3.5E-02 | 2.25 | Rattus norvegicus peroxiredoxin 2 (Prdx2), mRNA [NM_017169] |
| 580 | ***Rab1a*** | 4.5E-02 | 2.25 | Rattus norvegicus RAB1A, member RAS oncogene family (Rab1a), mRNA [NM_031090] |
| 581 | ***Brk1*** | 8.7E-03 | 2.25 | Rattus norvegicus BRICK1, SCAR/WAVE actin-nucleating complex subunit (Brk1), mRNA [NM_001195476] |
| 582 | ***Ciapin1*** | 1.4E-02 | 2.24 | Rattus norvegicus cytokine induced apoptosis inhibitor 1 (Ciapin1), mRNA [NM_001007689] |
| 583 | ***Wfdc2*** | 3.8E-02 | 2.24 | Rattus norvegicus WAP four-disulfide core domain 2 (Wfdc2), mRNA [NM_173109] |
| 584 | ***Commd9*** | 1.7E-02 | 2.24 | Rattus norvegicus COMM domain containing 9 (Commd9), mRNA [NM_001033692] |
| 585 | ***Capza1*** | 1.9E-02 | 2.24 | Rattus norvegicus capping protein (actin filament) muscle Z-line, alpha 1 (Capza1), mRNA [NM_001109625] |
| 586 | ***Mt2A*** | 1.9E-03 | 2.24 | Rattus norvegicus metallothionein 2A (Mt2A), mRNA [NM_001137564] |
| 587 | ***Mpv17l2*** | 4.7E-02 | 2.24 | Rattus norvegicus MPV17 mitochondrial membrane protein-like 2 (Mpv17l2), mRNA [NM_001106072] |
| 588 | ***Commd4*** | 3.1E-02 | 2.24 | Rattus norvegicus COMM domain containing 4 (Commd4), mRNA [NM_001108762] |
| 589 | ***Myeov2*** | 1.7E-02 | 2.23 | Rattus norvegicus myeloma overexpressed 2 (Myeov2), mRNA [NM_001109044] |
| 590 | ***LOC498154*** | 3.8E-02 | 2.23 | Rattus norvegicus hypothetical protein LOC498154 (LOC498154), mRNA [NM_001025033] |
| 591 | ***Metrnl*** | 1.7E-03 | 2.23 | Rattus norvegicus meteorin, glial cell differentiation regulator-like (Metrnl), mRNA [NM_001014104] |
| 592 | ***Ppt1*** | 4.8E-02 | 2.23 | Rattus norvegicus palmitoyl-protein thioesterase 1 (Ppt1), mRNA [NM_022502] |
| 593 | ***Notch3*** | 4.1E-02 | 2.23 | Rattus norvegicus notch 3 (Notch3), mRNA [NM_020087] |
| 594 | ***Ldhb*** | 1.1E-02 | 2.23 | Rattus norvegicus lactate dehydrogenase B (Ldhb), mRNA [NM_012595] |
| 595 | ***Dohh*** | 5.0E-02 | 2.22 | Rattus norvegicus deoxyhypusine hydroxylase/monooxygenase (Dohh), mRNA [NM_001025006] |
| 596 | ***Tubb5*** | 2.3E-02 | 2.22 | Rattus norvegicus tubulin, beta 5 class I (Tubb5), mRNA [NM_173102] |
| 597 | ***Ctps1*** | 3.7E-02 | 2.21 | Rattus norvegicus CTP synthase 1 (Ctps1), mRNA [NM_001134873] |
| 598 | ***Tmem69*** | 3.0E-02 | 2.21 | Rattus norvegicus transmembrane protein 69 (Tmem69), mRNA [NM_001035001] |
| 599 | ***Txndc11*** | 2.1E-03 | 2.21 | Rattus norvegicus thioredoxin domain containing 11 (Txndc11), mRNA [NM_001127532] |
| 600 | ***Cst7*** | 1.2E-02 | 2.21 | Rattus norvegicus cystatin F (leukocystatin) (Cst7), mRNA [NM_001106523] |
| 601 | ***Ccdc88b*** | 6.6E-05 | 2.21 | PREDICTED: Rattus norvegicus coiled-coil domain containing 88B (Ccdc88b), transcript variant X1, mRNA [XM_001072042] |
| 602 | ***Lrrk2*** | 4.2E-02 | 2.21 | Rattus norvegicus leucine-rich repeat kinase 2 (Lrrk2), mRNA [NM_001191789] |
| 603 | ***LOC102552334*** | 6.5E-04 | 2.21 | PREDICTED: Rattus norvegicus uncharacterized LOC102552334 (LOC102552334), ncRNA [XR_358982] |
| 604 | ***Sel1l3*** | 2.6E-03 | 2.21 | PREDICTED: Rattus norvegicus sel-1 suppressor of lin-12-like 3 (C. elegans) (Sel1l3), transcript variant X1, mRNA [XM_006251033] |
| 605 | ***Rtn3*** | 1.7E-02 | 2.20 | Rattus norvegicus reticulon 3 (Rtn3), transcript variant 1, mRNA [NM_080909] |
| 606 | ***Slc25a11*** | 3.2E-02 | 2.20 | Rattus norvegicus solute carrier family 25 (mitochondrial carrier; oxoglutarate carrier), member 11 (Slc25a11), mRNA [NM_022398] |
| 607 | ***Romo1*** | 3.7E-02 | 2.20 | Rattus norvegicus reactive oxygen species modulator 1 (Romo1), mRNA [NM_001195490] |
| 608 | ***Lyz2*** | 8.1E-03 | 2.20 | Rattus norvegicus lysozyme 2 (Lyz2), mRNA [NM_012771] |
| 609 | ***Psmd8*** | 4.0E-02 | 2.20 | Rattus norvegicus proteasome (prosome, macropain) 26S subunit, non-ATPase, 8 (Psmd8), mRNA [NM_001100831] |
| 610 | ***Ahcyl2*** | 1.4E-02 | 2.19 | Rattus norvegicus adenosylhomocysteinase-like 2 (Ahcyl2), mRNA [NM_001173510] |
| 611 | ***Uchl3*** | 1.6E-02 | 2.19 | Rattus norvegicus ubiquitin carboxyl-terminal esterase L3 (ubiquitin thiolesterase) (Uchl3), mRNA [NM_001110165] |
| 612 | ***Ppil1*** | 1.2E-03 | 2.19 | Rattus norvegicus peptidylprolyl isomerase (cyclophilin)-like 1 (Ppil1), mRNA [NM_001034188] |
| 613 | ***Cct7*** | 1.1E-02 | 2.19 | Rattus norvegicus chaperonin containing Tcp1, subunit 7 (eta) (Cct7), mRNA [NM_001106603] |
| 614 | ***LOC685438*** | 1.8E-04 | 2.19 | PREDICTED: Rattus norvegicus paired immunoglobulin-like type 2 receptor beta-2-like (LOC685438), transcript variant X2, mRNA [XM_006221313] |
| 615 | ***Jtb*** | 3.4E-02 | 2.19 | Rattus norvegicus jumping translocation breakpoint (Jtb), mRNA [NM_019213] |
| 616 | ***Smap2*** | 4.8E-02 | 2.18 | Rattus norvegicus small ArfGAP2 (Smap2), mRNA [NM_001100669] |
| 617 | ***RGD1564450*** | 4.5E-04 | 2.18 | Rattus norvegicus RGD1564450 (RGD1564450), mRNA [NM_001106377] |
| 618 | ***Nadk*** | 2.1E-02 | 2.18 | Rattus norvegicus NAD kinase (Nadk), mRNA [NM_001109678] |
| 619 | ***Mrpl36*** | 9.1E-03 | 2.18 | Rattus norvegicus mitochondrial ribosomal protein L36 (Mrpl36), mRNA [NM_001108879] |
| 620 | ***Trappc2l*** | 2.9E-02 | 2.17 | Rattus norvegicus trafficking protein particle complex 2-like (Trappc2l), mRNA [NM_001106193] |
| 621 | ***Dusp11*** | 2.3E-02 | 2.17 | Rattus norvegicus dual specificity phosphatase 11 (RNA/RNP complex 1-interacting) (Dusp11), mRNA [NM_001025650] |
| 622 | ***Uqcrfs1*** | 3.4E-02 | 2.17 | Rattus norvegicus ubiquinol-cytochrome c reductase, Rieske iron-sulfur polypeptide 1 (Uqcrfs1), mRNA [NM_001008888] |
| 623 | ***Slc39a8*** | 2.6E-02 | 2.17 | Rattus norvegicus solute carrier family 39 (zinc transporter), member 8 (Slc39a8), mRNA [NM_001011952] |
| 624 | ***LOC100364956*** | 6.9E-03 | 2.17 | PREDICTED: Rattus norvegicus MHC class Ib alpha chain-like (LOC100364956), partial mRNA [XM_006227191] |
| 625 | ***LOC303448*** | 4.1E-04 | 2.17 | Rattus norvegicus similar to glyceraldehyde-3-phosphate dehydrogenase (LOC303448), mRNA [NM_001037190] |
| 626 | ***RGD2320734*** | 3.2E-03 | 2.16 | similar to ubiquinol-cytochrome c reductase complex 7.2kDa protein isoform b (LOC685322), mRNA [Source:RefSeq mRNA;Acc:NM_001170465] [ENSRNOT00000012614] |
| 627 | ***Cpeb2*** | 4.7E-03 | 2.16 | PREDICTED: Rattus norvegicus cytoplasmic polyadenylation element binding protein 2 (Cpeb2), transcript variant X1, mRNA [XM_006251080] |
| 628 | ***Gpr56*** | 4.4E-03 | 2.16 | Rattus norvegicus G protein-coupled receptor 56 (Gpr56), mRNA [NM_152242] |
| 629 | ***Fes*** | 1.5E-02 | 2.15 | Rattus norvegicus feline sarcoma oncogene (Fes), mRNA [NM_001108488] |
| 630 | ***Gapdh*** | 1.0E-03 | 2.15 | Rattus norvegicus glyceraldehyde-3-phosphate dehydrogenase (Gapdh), mRNA [NM_017008] |
| 631 | ***Fbxo25*** | 3.3E-02 | 2.14 | Rattus norvegicus F-box protein 25 (Fbxo25), mRNA [NM_001014239] |
| 632 | ***Apex1*** | 3.1E-02 | 2.14 | Rattus norvegicus APEX nuclease (multifunctional DNA repair enzyme) 1 (Apex1), mRNA [NM_024148] |
| 633 | ***Usp8*** | 4.2E-02 | 2.14 | Rattus norvegicus ubiquitin specific peptidase 8 (Usp8), mRNA [NM_001106502] |
| 634 | ***Ndufb2*** | 3.8E-02 | 2.14 | Rattus norvegicus NADH dehydrogenase (ubiquinone) 1 beta subcomplex, 2 (Ndufb2), mRNA [NM_001108624] |
| 635 | ***Slc41a1*** | 1.7E-03 | 2.14 | Rattus norvegicus solute carrier family 41 (magnesium transporter), member 1 (Slc41a1), mRNA [NM_001108855] |
| 636 | ***Atp6v0e1*** | 3.0E-02 | 2.14 | Rattus norvegicus ATPase, H+ transporting, lysosomal, V0 subunit e1 (Atp6v0e1), mRNA [NM_053578] |
| 637 | ***Impdh2*** | 4.5E-02 | 2.13 | Rattus norvegicus IMP (inosine 5'-monophosphate) dehydrogenase 2 (Impdh2), mRNA [NM_199099] |
| 638 | ***Cct3*** | 1.5E-02 | 2.13 | Rattus norvegicus chaperonin containing Tcp1, subunit 3 (gamma) (Cct3), mRNA [NM_199091] |
| 639 | ***Dram1*** | 2.4E-04 | 2.13 | Rattus norvegicus DNA-damage regulated autophagy modulator 1 (Dram1), mRNA [NM_001173427] |
| 640 | ***Trpm6*** | 5.9E-03 | 2.13 | PREDICTED: Rattus norvegicus transient receptor potential cation channel, subfamily M, member 6 (Trpm6), transcript variant X1, mRNA [XM_006223666] |
| 641 | ***Psmb2*** | 4.2E-02 | 2.13 | Rattus norvegicus proteasome (prosome, macropain) subunit, beta type 2 (Psmb2), mRNA [NM_017284] |
| 642 | ***Got2*** | 1.4E-03 | 2.13 | Rattus norvegicus glutamic-oxaloacetic transaminase 2, mitochondrial (Got2), mRNA [NM_013177] |
| 643 | ***Atpif1*** | 2.2E-02 | 2.12 | Rattus norvegicus ATPase inhibitory factor 1 (Atpif1), mRNA [NM_012915] |
| 644 | ***Cndp1*** | 2.9E-02 | 2.12 | Rattus norvegicus carnosine dipeptidase 1 (metallopeptidase M20 family) (Cndp1), mRNA [NM_001007687] |
| 645 | ***LOC100364062*** | 1.2E-04 | 2.12 | PREDICTED: Rattus norvegicus M2 pyruvate kinase-like (LOC100364062), mRNA [XM_003751952] |
| 646 | ***Ap3b1*** | 9.9E-03 | 2.12 | Rattus norvegicus adaptor-related protein complex 3, beta 1 subunit (Ap3b1), mRNA [NM_001107646] |
| 647 | ***LOC100361067*** | 4.6E-03 | 2.12 | proteasome subunit alpha type 3-like (Psma3l), mRNA [Source:RefSeq mRNA;Acc:NM_001004094] [ENSRNOT00000011446] |
| 648 | ***LOC691015*** | 1.3E-02 | 2.12 | PREDICTED: Rattus norvegicus NADH dehydrogenase [ubiquinone] 1 alpha subcomplex subunit 2-like (LOC691015), mRNA [XM_001069534] |
| 649 | ***Uchl3*** | 7.1E-03 | 2.12 | Rattus norvegicus ubiquitin carboxyl-terminal esterase L3 (ubiquitin thiolesterase) (Uchl3), mRNA [NM_001110165] |
| 650 | ***Vps36*** | 1.1E-02 | 2.12 | Rattus norvegicus vacuolar protein sorting 36 homolog (S. cerevisiae) (Vps36), mRNA [NM_001106092] |
| 651 | ***Rplp0*** | 2.7E-02 | 2.12 | Rattus norvegicus ribosomal protein, large, P0 (Rplp0), mRNA [NM_022402] |
| 652 | ***Sts*** | 3.6E-02 | 2.12 | Rattus norvegicus steroid sulfatase (microsomal), isozyme S (Sts), mRNA [NM_012661] |
| 653 | ***Cdc123*** | 2.6E-02 | 2.11 | Rattus norvegicus cell division cycle 123 (Cdc123), mRNA [NM_053877] |
| 654 | ***Ctps2*** | 1.4E-02 | 2.11 | Rattus norvegicus CTP synthase 2 (Ctps2), mRNA [NM_001034998] |
| 655 | ***Mob1a*** | 2.9E-02 | 2.11 | Rattus norvegicus MOB kinase activator 1A (Mob1a), mRNA [NM_001033891] |
| 656 | ***Tmem116*** | 2.9E-02 | 2.11 | Rattus norvegicus transmembrane protein 116 (Tmem116), mRNA [NM_001159625] |
| 657 | ***Hprt1*** | 9.1E-03 | 2.11 | Rattus norvegicus hypoxanthine phosphoribosyltransferase 1 (Hprt1), mRNA [NM_012583] |
| 658 | ***Pgs1*** | 2.2E-02 | 2.10 | PREDICTED: Rattus norvegicus phosphatidylglycerophosphate synthase 1 (Pgs1), transcript variant X2, mRNA [XM_006220954] |
| 659 | ***Actr3*** | 7.7E-03 | 2.10 | Rattus norvegicus ARP3 actin-related protein 3 homolog (yeast) (Actr3), mRNA [NM_031068] |
| 660 | ***Polr2j*** | 3.2E-02 | 2.10 | Rattus norvegicus polymerase (RNA) II (DNA directed) polypeptide J (Polr2j), mRNA [NM_001105921] |
| 661 | ***Tbc1d2*** | 1.8E-02 | 2.10 | Rattus norvegicus TBC1 domain family, member 2 (Tbc1d2), mRNA [NM_001107933] |
| 662 | ***Gpi*** | 2.1E-02 | 2.09 | Rattus norvegicus glucose-6-phosphate isomerase (Gpi), mRNA [NM_207592] |
| 663 | ***Hdlbp*** | 2.8E-02 | 2.09 | Rattus norvegicus high density lipoprotein binding protein (Hdlbp), mRNA [NM_172039] |
| 664 | ***Enpp3*** | 1.3E-03 | 2.08 | Rattus norvegicus ectonucleotide pyrophosphatase/phosphodiesterase 3 (Enpp3), mRNA [NM_019370] |
| 665 | ***Park7*** | 2.9E-02 | 2.08 | Rattus norvegicus parkinson protein 7 (Park7), transcript variant 5, mRNA [NM_057143] |
| 666 | ***Por*** | 1.5E-02 | 2.08 | Rattus norvegicus P450 (cytochrome) oxidoreductase (Por), mRNA [NM_031576] |
| 667 | ***Pfn1*** | 9.7E-03 | 2.08 | Rattus norvegicus profilin 1 (Pfn1), mRNA [NM_022511] |
| 668 | ***Mrpl27*** | 4.8E-02 | 2.08 | Rattus norvegicus mitochondrial ribosomal protein L27 (Mrpl27), mRNA [NM_001105831] |
| 669 | ***Calm3*** | 3.4E-02 | 2.07 | Rattus norvegicus calmodulin 3 (Calm3), mRNA [NM_012518] |
| 670 | ***Tcp1*** | 1.4E-02 | 2.07 | Rattus norvegicus t-complex 1 (Tcp1), mRNA [NM_012670] |
| 671 | ***Prkcsh*** | 4.8E-02 | 2.07 | Rattus norvegicus protein kinase C substrate 80K-H (Prkcsh), mRNA [NM_001106806] |
| 672 | ***Dnajc15*** | 9.8E-03 | 2.07 | Rattus norvegicus DnaJ (Hsp40) homolog, subfamily C, member 15 (Dnajc15), mRNA [NM_001106050] |
| 673 | ***Ppp4c*** | 2.2E-02 | 2.07 | Rattus norvegicus protein phosphatase 4, catalytic subunit (Ppp4c), mRNA [NM_134359] |
| 674 | ***Ermp1*** | 3.9E-02 | 2.07 | Rattus norvegicus endoplasmic reticulum metallopeptidase 1 (Ermp1), mRNA [NM_184050] |
| 675 | ***Atxn10*** | 2.4E-02 | 2.07 | Rattus norvegicus ataxin 10 (Atxn10), mRNA [NM_133313] |
| 676 | ***Ctsf*** | 8.5E-03 | 2.06 | Rattus norvegicus cathepsin F (Ctsf), mRNA [NM_001034110] |
| 677 | ***Tnfsf12*** | 3.5E-03 | 2.06 | Rattus norvegicus tumor necrosis factor ligand superfamily member 12 (Tnfsf12), mRNA [NM_001001513] |
| 678 | ***RGD1562690*** | 6.1E-03 | 2.06 | PREDICTED: Rattus norvegicus L-lactate dehydrogenase A chain-like (RGD1562690), mRNA [XM_003750458] |
| 679 | ***Tmed5*** | 3.7E-02 | 2.06 | Rattus norvegicus transmembrane emp24 protein transport domain containing 5 (Tmed5), mRNA [NM_001007619] |
| 680 | ***Slc16a6*** | 1.5E-02 | 2.06 | Rattus norvegicus solute carrier family 16, member 6 (Slc16a6), mRNA [NM_198760] |
| 681 | ***Tuba4a*** | 1.0E-02 | 2.06 | Rattus norvegicus tubulin, alpha 4A (Tuba4a), mRNA [NM_001007004] |
| 682 | ***Cisd2*** | 1.5E-04 | 2.06 | Rattus norvegicus CDGSH iron sulfur domain 2 (Cisd2), mRNA [NM_001191608] |
| 683 | ***RGD1563581*** | 4.5E-02 | 2.05 | PREDICTED: Rattus norvegicus protein S100-A11-like (RGD1563581), mRNA [XM_001064539] |
| 684 | ***Tcp1*** | 2.0E-02 | 2.05 | Rattus norvegicus t-complex 1 (Tcp1), mRNA [NM_012670] |
| 685 | ***Sil1*** | 4.1E-02 | 2.05 | Rattus norvegicus SIL1 nucleotide exchange factor (Sil1), mRNA [NM_199376] |
| 686 | ***Dpp3*** | 3.4E-02 | 2.04 | Rattus norvegicus dipeptidylpeptidase 3 (Dpp3), mRNA [NM_053748] |
| 687 | ***S100a11*** | 3.4E-02 | 2.04 | Rattus norvegicus S100 calcium binding protein A11 (S100a11), mRNA [NM_001004095] |
| 688 | ***Smurf1*** | 3.1E-02 | 2.04 | Rattus norvegicus SMAD specific E3 ubiquitin protein ligase 1 (Smurf1), mRNA [NM_001109598] |
| 689 | ***Hadha*** | 1.9E-02 | 2.03 | Rattus norvegicus hydroxyacyl-CoA dehydrogenase/3-ketoacyl-CoA thiolase/enoyl-CoA hydratase (trifunctional protein), alpha subunit (Hadha), mRNA [NM_130826] |
| 690 | ***Ccdc97*** | 3.0E-02 | 2.03 | Rattus norvegicus coiled-coil domain containing 97 (Ccdc97), mRNA [NM_001106235] |
| 691 | ***Tuba1c*** | 7.0E-03 | 2.03 | Rattus norvegicus tubulin, alpha 1C (Tuba1c), mRNA [NM_001011995] |
| 692 | ***Eif3c*** | 3.8E-02 | 2.03 | Rattus norvegicus eukaryotic translation initiation factor 3, subunit C (Eif3c), mRNA [NM_001100662] |
| 693 | ***Mrpl23*** | 2.3E-02 | 2.03 | Rattus norvegicus mitochondrial ribosomal protein L23 (Mrpl23), mRNA [NM_022529] |
| 694 | ***Caprin1*** | 2.3E-02 | 2.03 | Rattus norvegicus cell cycle associated protein 1 (Caprin1), mRNA [NM_001012185] |
| 695 | ***Ncoa1*** | 1.9E-02 | 2.03 | Rattus norvegicus nuclear receptor coactivator 1 (Ncoa1), mRNA [NM_001108012] |
| 696 | ***Plin3*** | 2.4E-02 | 2.03 | Protein Plin3 [Source:UniProtKB/TrEMBL;Acc:M0RA08] [ENSRNOT00000070978] |
| 697 | ***Noc4l*** | 4.4E-02 | 2.02 | Rattus norvegicus nucleolar complex associated 4 homolog (S. cerevisiae) (Noc4l), mRNA [NM_001014129] |
| 698 | ***Ndufs3*** | 4.7E-02 | 2.02 | Rattus norvegicus NADH dehydrogenase (ubiquinone) Fe-S protein 3 (Ndufs3), mRNA [NM_001106489] |
| 699 | ***Spc24*** | 1.1E-02 | 2.02 | PREDICTED: Rattus norvegicus SPC24, NDC80 kinetochore complex component (Spc24), mRNA [XM_003750448] |
| 700 | ***Gsto1*** | 2.3E-03 | 2.02 | Rattus norvegicus glutathione S-transferase omega 1 (Gsto1), mRNA [NM_001007602] |
| 701 | ***Cnih4*** | 4.1E-02 | 2.02 | Rattus norvegicus cornichon family AMPA receptor auxiliary protein 4 (Cnih4), mRNA [NM_001105981] |
| 702 | ***Lrp2*** | 2.5E-02 | 2.02 | Rattus norvegicus low density lipoprotein receptor-related protein 2 (Lrp2), mRNA [NM_030827] |
| 703 | ***Oser1*** | 4.1E-02 | 2.02 | Rattus norvegicus oxidative stress responsive serine-rich 1 (Oser1), mRNA [NM_201560] |
| 704 | ***LOC365654*** | 3.6E-02 | 2.02 | PREDICTED: Rattus norvegicus ATP synthase subunit beta, mitochondrial-like (LOC365654), mRNA [XM_006223989] |
| 705 | ***Arf1*** | 1.3E-02 | 2.02 | Rattus norvegicus ADP-ribosylation factor 1 (Arf1), mRNA [NM_022518] |
| 706 | ***Cox5a*** | 7.9E-03 | 2.01 | Rattus norvegicus cytochrome c oxidase, subunit Va (Cox5a), mRNA [NM_145783] |
| 707 | ***Tapbp*** | 1.3E-02 | 2.01 | Rattus norvegicus TAP binding protein (tapasin) (Tapbp), mRNA [NM_033098] |
| 708 | ***Copz1*** | 1.3E-02 | 2.01 | Rattus norvegicus coatomer protein complex, subunit zeta 1 (Copz1), mRNA [NM_001108117] |
| 709 | ***Ppme1*** | 3.9E-02 | 2.01 | Rattus norvegicus protein phosphatase methylesterase 1 (Ppme1), mRNA [NM_001191838] |
| 710 | ***Sod1*** | 2.6E-02 | 2.01 | Rattus norvegicus superoxide dismutase 1, soluble (Sod1), mRNA [NM_017050] |
| 711 | ***Ndufa2*** | 1.5E-02 | 2.01 | Rattus norvegicus NADH dehydrogenase (ubiquinone) 1 alpha subcomplex, 2 (Ndufa2), mRNA [NM_001106153] |
| 712 | ***Pgam1*** | 5.4E-03 | 2.00 | Rattus norvegicus phosphoglycerate mutase 1 (brain) (Pgam1), mRNA [NM_053290] |
