# Supplementary material for: Microarray analysis of gene expression in lung tissues of indium-exposed rats: possible roles of S100 proteins in lung diseases
Source: Arch Toxicol. 2024 Nov 8;99(1):245–58. doi: 10.1007/s00204-024-03897-x (PMC11742277; doi:10.1007/s00204-024-03897-x)
Supplement: Supplementary file 2 — Supplementary file2 (DOCX 77 KB) [file 204_2024_3897_MOESM2_ESM.docx]

**Table S2** Downregulated genes in indium-exposed rats

**(A) In_2_O_3_-exposed rats**

| Rank | ***GeneSymbol*** | *p* (ANOVA) | fold | Description |
| --- | --- | --- | --- | --- |
| 1 | ***Serpinb10*** | 1.1E-02 | -6.11 | Rattus norvegicus serpin peptidase inhibitor, clade B (ovalbumin), member 10 (Serpinb10), mRNA [NM_153733] |
| 2 | ***Pla2g2d*** | 2.2E-04 | -5.71 | Rattus norvegicus phospholipase A2, group IID (Pla2g2d), mRNA [NM_001013428] |
| 3 | ***Slc29a1*** | 3.9E-02 | -5.41 | Rattus norvegicus solute carrier family 29 (equilibrative nucleoside transporter), member 1 (Slc29a1), mRNA [NM_031684] |
| 4 | ***Tbx2*** | 1.8E-03 | -4.99 | Rattus norvegicus T-box 2 (Tbx2), mRNA [NM_001107033] |
| 5 | ***Kcne2*** | 4.0E-02 | -3.81 | Rattus norvegicus potassium voltage-gated channel, Isk-related family, member 2 (Kcne2), mRNA [NM_133603] |
| 6 | ***Hpgd*** | 1.0E-02 | -3.81 | Rattus norvegicus hydroxyprostaglandin dehydrogenase 15 (NAD) (Hpgd), mRNA [NM_024390] |
| 7 | ***Cbx7*** | 2.0E-02 | -3.74 | Rattus norvegicus chromobox homolog 7 (Cbx7), mRNA [NM_199117] |
| 8 | ***Fmo1*** | 1.7E-02 | -3.64 | Rattus norvegicus flavin containing monooxygenase 1 (Fmo1), mRNA [NM_012792] |
| 9 | ***Ptprr*** | 4.1E-04 | -3.48 | Rattus norvegicus protein tyrosine phosphatase, receptor type, R (Ptprr), transcript variant 1, mRNA [NM_053594] |
| 10 | ***Tuba1a*** | 8.7E-04 | -3.34 | Rattus norvegicus tubulin, alpha 1A (Tuba1a), mRNA [NM_022298] |
| 11 | ***Ablim3*** | 1.9E-03 | -3.25 | Rattus norvegicus actin binding LIM protein family, member 3 (Ablim3), mRNA [NM_001191698] |
| 12 | ***Il33*** | 7.2E-04 | -3.16 | Rattus norvegicus interleukin 33 (Il33), mRNA [NM_001014166] |
| 13 | ***Tppp3*** | 2.7E-03 | -3.14 | Rattus norvegicus tubulin polymerization-promoting protein family member 3 (Tppp3), mRNA [NM_001009639] |
| 14 | ***Gja5*** | 8.6E-03 | -3.10 | Rattus norvegicus gap junction protein, alpha 5 (Gja5), mRNA [NM_019280] |
| 15 | ***Ooep*** | 8.5E-04 | -3.01 | PREDICTED: Rattus norvegicus oocyte expressed protein (Ooep), misc_RNA [XR_348628] |
| 16 | ***Apol3*** | 7.6E-03 | -2.95 | Rattus norvegicus apolipoprotein L, 3 (Apol3), transcript variant 2, mRNA [NM_001013175] |
| 17 | ***Clec14a*** | 2.7E-02 | -2.92 | Rattus norvegicus C-type lectin domain family 14, member A (Clec14a), mRNA [NM_001014077] |
| 18 | ***Dusp14*** | 8.4E-06 | -2.82 | Rattus norvegicus dual specificity phosphatase 14 (Dusp14), transcript variant 1, mRNA [NM_001079893] |
| 19 | ***Akap5*** | 2.2E-03 | -2.80 | Rattus norvegicus A kinase (PRKA) anchor protein 5 (Akap5), mRNA [NM_133515] |
| 20 | ***Synm*** | 2.0E-02 | -2.77 | Rattus norvegicus synemin, intermediate filament protein (Synm), mRNA [NM_001134858] |
| 21 | ***Apol3*** | 8.3E-03 | -2.74 | Rattus norvegicus apolipoprotein L, 3 (Apol3), transcript variant 1, mRNA [NM_001277359] |
| 22 | ***Rab6b*** | 9.2E-04 | -2.73 | Rattus norvegicus RAB6B, member RAS oncogene family (Rab6b), mRNA [NM_001108775] |
| 23 | ***Mycbpap*** | 2.7E-02 | -2.73 | Rattus norvegicus Mycbp associated protein (Mycbpap), mRNA [NM_001009482] |
| 24 | ***Ooep*** | 6.2E-05 | -2.70 | PREDICTED: Rattus norvegicus oocyte expressed protein (Ooep), misc_RNA [XR_348628] |
| 25 | ***Cdc42bpg*** | 1.0E-02 | -2.69 | Rattus norvegicus CDC42 binding protein kinase gamma (DMPK-like) (Cdc42bpg), mRNA [NM_001130013] |
| 26 | ***Ccdc85b*** | 5.2E-04 | -2.65 | PREDICTED: Rattus norvegicus coiled-coil domain containing 85B (Ccdc85b), mRNA [XM_006223613] |
| 27 | ***Dusp26*** | 3.2E-03 | -2.64 | Rattus norvegicus dual specificity phosphatase 26 (putative) (Dusp26), mRNA [NM_001012352] |
| 28 | ***Cav1*** | 2.4E-02 | -2.62 | Rattus norvegicus caveolin 1, caveolae protein (Cav1), transcript variant 1, mRNA [NM_031556] |
| 29 | ***Meis1*** | 4.3E-02 | -2.62 | Rattus norvegicus Meis homeobox 1 (Meis1), mRNA [NM_001134702] |
| 30 | ***Tspan8*** | 3.8E-03 | -2.60 | Rattus norvegicus tetraspanin 8 (Tspan8), mRNA [NM_133526] |
| 31 | ***Ripply3*** | 2.7E-04 | -2.59 | Rattus norvegicus ripply transcriptional repressor 3 (Ripply3), mRNA [NM_001105892] |
| 32 | ***Klf4*** | 1.9E-03 | -2.58 | Rattus norvegicus Kruppel-like factor 4 (gut) (Klf4), mRNA [NM_053713] |
| 33 | ***Slc9a3r2*** | 1.1E-02 | -2.57 | Rattus norvegicus solute carrier family 9, subfamily A (NHE3, cation proton antiporter 3), member 3 regulator 2 (Slc9a3r2), mRNA [NM_053811] |
| 34 | ***S100a16*** | 1.2E-02 | -2.56 | Rattus norvegicus S100 calcium binding protein A16 (S100a16), mRNA [NM_001108557] |
| 35 | ***Wscd1*** | 8.3E-03 | -2.56 | Rattus norvegicus WSC domain containing 1 (Wscd1), mRNA [NM_001024234] |
| 36 | ***Artn*** | 3.2E-03 | -2.50 | Rattus norvegicus artemin (Artn), mRNA [NM_053397] |
| 37 | ***Npy*** | 3.7E-03 | -2.47 | Rattus norvegicus neuropeptide Y (Npy), mRNA [NM_012614] |
| 38 | ***Epn3*** | 2.4E-03 | -2.47 | Rattus norvegicus epsin 3 (Epn3), mRNA [NM_001024791] |
| 39 | ***Tp53i11*** | 6.5E-03 | -2.47 | Rattus norvegicus tumor protein p53 inducible protein 11 (Tp53i11), mRNA [NM_001107749] |
| 40 | ***H1f0*** | 7.0E-04 | -2.45 | Rattus norvegicus H1 histone family, member 0 (H1f0), mRNA [NM_012578] |
| 41 | ***Tacstd2*** | 6.8E-03 | -2.45 | Rattus norvegicus tumor-associated calcium signal transducer 2 (Tacstd2), mRNA [NM_001009540] |
| 42 | ***Hist1h1d*** | 1.2E-02 | -2.44 | Rattus norvegicus histone cluster 1, H1d (Hist1h1d), mRNA [NM_133285] |
| 43 | ***Nfatc4*** | 1.2E-02 | -2.42 | Rattus norvegicus nuclear factor of activated T-cells, cytoplasmic, calcineurin-dependent 4 (Nfatc4), mRNA [NM_001107264] |
| 44 | ***Gng13*** | 1.1E-02 | -2.41 | Rattus norvegicus guanine nucleotide binding protein (G protein), gamma 13 (Gng13), mRNA [NM_001135918] |
| 45 | ***Tp53bp2*** | 1.0E-03 | -2.36 | Protein Tp53bp2 [Source:UniProtKB/TrEMBL;Acc:F1M5H6] [ENSRNOT00000004330] |
| 46 | ***Ahnak*** | 7.8E-03 | -2.34 | Rattus norvegicus AHNAK nucleoprotein (Ahnak), mRNA [NM_001191951] |
| 47 | ***Dynlrb2*** | 1.4E-02 | -2.32 | Rattus norvegicus dynein light chain roadblock-type 2 (Dynlrb2), mRNA [NM_001108451] |
| 48 | ***Pkig*** | 1.8E-02 | -2.32 | Rattus norvegicus protein kinase inhibitor, gamma (Pkig), mRNA [NM_153469] |
| 49 | ***Emid1*** | 3.5E-03 | -2.31 | Rattus norvegicus EMI domain containing 1 (Emid1), mRNA [NM_001109467] |
| 50 | ***Gimap7*** | 1.3E-02 | -2.30 | Rattus norvegicus GTPase, IMAP family member 7 (Gimap7), mRNA [NM_001024328] |
| 51 | ***Mettl25*** | 1.4E-02 | -2.29 | Rattus norvegicus methyltransferase like 25 (Mettl25), mRNA [NM_001108089] |
| 52 | ***Apol3*** | 1.1E-03 | -2.28 | Rattus norvegicus TL0AEA54YP09 mRNA sequence. [FQ225154] |
| 53 | ***Pacsin3*** | 3.8E-02 | -2.27 | Rattus norvegicus protein kinase C and casein kinase substrate in neurons 3 (Pacsin3), mRNA [NM_001009966] |
| 54 | ***Dynlrb2*** | 1.7E-02 | -2.27 | Rattus norvegicus dynein light chain roadblock-type 2 (Dynlrb2), mRNA [NM_001108451] |
| 55 | ***Nrp1*** | 4.2E-03 | -2.24 | Rattus norvegicus neuropilin 1 (Nrp1), mRNA [NM_145098] |
| 56 | ***Anxa8*** | 6.8E-03 | -2.22 | Rattus norvegicus annexin A8 (Anxa8), mRNA [NM_001031654] |
| 57 | ***Bbs4*** | 2.4E-02 | -2.20 | Rattus norvegicus Bardet-Biedl syndrome 4 (Bbs4), mRNA [NM_001106826] |
| 58 | ***Clec2dl1*** | 2.0E-02 | -2.19 | Rattus norvegicus C-type lectin domain family 2 member D-like 1 (Clec2dl1), mRNA [NM_001085404] |
| 59 | ***Kctd3*** | 1.4E-04 | -2.18 | Rattus norvegicus potassium channel tetramerization domain containing 3 (Kctd3), mRNA [NM_001107199] |
| 60 | ***Ehd1*** | 3.8E-02 | -2.18 | Rattus norvegicus EH-domain containing 1 (Ehd1), mRNA [NM_001011939] |
| 61 | ***Hopx*** | 5.2E-03 | -2.17 | Rattus norvegicus HOP homeobox (Hopx), mRNA [NM_133621] |
| 62 | ***Rassf3*** | 6.7E-03 | -2.17 | Rattus norvegicus Ras association (RalGDS/AF-6) domain family member 3 (Rassf3), mRNA [NM_001108747] |
| 63 | ***Calm2*** | 3.8E-03 | -2.16 | Rattus norvegicus calmodulin 2 (Calm2), mRNA [NM_017326] |
| 64 | ***LOC685179*** | 2.4E-02 | -2.16 | PREDICTED: Rattus norvegicus SWI/SNF complex subunit SMARCC2-like (LOC685179), transcript variant X5, mRNA [XM_002729767] |
| 65 | ***Npy*** | 5.4E-03 | -2.16 | Rattus norvegicus neuropeptide Y (Npy), mRNA [NM_012614] |
| 66 | ***Slc38a5*** | 2.7E-02 | -2.15 | Rattus norvegicus solute carrier family 38, member 5 (Slc38a5), mRNA [NM_138854] |
| 67 | ***Hs3st6*** | 3.2E-02 | -2.14 | Rattus norvegicus heparan sulfate (glucosamine) 3-O-sulfotransferase 6 (Hs3st6), mRNA [NM_001109450] |
| 68 | ***LOC680885*** | 3.9E-02 | -2.13 | Rattus norvegicus hypothetical protein LOC680885 (LOC680885), mRNA [NM_001109431] |
| 69 | ***RGD1310587*** | 2.0E-04 | -2.12 | Rattus norvegicus similar to hypothetical protein FLJ14146 (RGD1310587), mRNA [NM_001100857] |
| 70 | ***Fus*** | 2.1E-02 | -2.10 | Rattus norvegicus fused in sarcoma (Fus), mRNA [NM_001012137] |
| 71 | ***Fus*** | 1.2E-02 | -2.10 | Rattus norvegicus fused in sarcoma (Fus), mRNA [NM_001012137] |
| 72 | ***RT1-T24-1*** | 2.3E-03 | -2.08 | Rattus norvegicus RT1 class I, locus T24, gene 1 (RT1-T24-1), mRNA [NM_001008858] |
| 73 | ***Sec14l3*** | 2.4E-02 | -2.07 | Rattus norvegicus SEC14-like 3 (S. cerevisiae) (Sec14l3), mRNA [NM_022608] |
| 74 | ***Sox4*** | 2.0E-02 | -2.06 | Rattus norvegicus SRY (sex determining region Y)-box 4 (Sox4), mRNA [NM_001271205] |
| 75 | ***Bend5*** | 2.3E-04 | -2.06 | Rattus norvegicus BEN domain containing 5 (Bend5), mRNA [NM_001108672] |
| 76 | ***Efcab4a*** | 2.6E-03 | -2.05 | Rattus norvegicus EF-hand calcium binding domain 4A (Efcab4a), mRNA [NM_001127541] |
| 77 | ***Selm*** | 6.6E-03 | -2.05 | Rattus norvegicus selenoprotein M (Selm), mRNA [NM_001115013] |
| 78 | ***Acsm5*** | 3.0E-02 | -2.05 | Rattus norvegicus acyl-CoA synthetase medium-chain family member 5 (Acsm5), mRNA [NM_001014162] |
| 79 | ***S100a6*** | 4.4E-02 | -2.04 | Rattus norvegicus S100 calcium binding protein A6 (S100a6), mRNA [NM_053485] |
| 80 | ***Cc2d2a*** | 1.6E-02 | -2.03 | coiled-coil and C2 domain containing 2A [Source:MGI Symbol;Acc:MGI:1924487] [ENSRNOT00000006968] |
| 81 | ***Nup210*** | 7.2E-03 | -2.03 | Rattus norvegicus nucleoporin 210 (Nup210), mRNA [NM_053322] |
| 82 | ***Adm*** | 1.7E-02 | -2.03 | Rattus norvegicus adrenomedullin (Adm), mRNA [NM_012715] |
| 83 | ***Selenbp1*** | 1.5E-02 | -2.02 | Rattus norvegicus selenium binding protein 1 (Selenbp1), mRNA [NM_080892] |
| 84 | ***Sox18*** | 1.2E-02 | -2.02 | Rattus norvegicus SRY (sex determining region Y)-box 18 (Sox18), mRNA [NM_001024781] |
| 85 | ***Akap7*** | 2.9E-02 | -2.00 | A kinase (PRKA) anchor protein 7 (Akap7), mRNA [Source:RefSeq mRNA;Acc:NM_001001801] [ENSRNOT00000017617] |
| 86 | ***Mfng*** | 5.6E-03 | -2.00 | Rattus norvegicus MFNG O-fucosylpeptide 3-beta-N-acetylglucosaminyltransferase (Mfng), mRNA [NM_199110] |

**(B) ITO-exposed rats**

| Rank | ***GeneSymbol*** | *p* (ANOVA) | fold | Description |
| --- | --- | --- | --- | --- |
| 1 | ***Pla2g2d*** | 2.2E-04 | -10.38 | Rattus norvegicus phospholipase A2, group IID (Pla2g2d), mRNA [NM_001013428] |
| 2 | ***Serpinb10*** | 1.1E-02 | -5.47 | Rattus norvegicus serpin peptidase inhibitor, clade B (ovalbumin), member 10 (Serpinb10), mRNA [NM_153733] |
| 3 | ***Vom2r75*** | 2.0E-02 | -5.23 | Rattus norvegicus vomeronasal 2 receptor, 75 (Vom2r75), mRNA [NM_173320] |
| 4 | ***Vmac*** | 2.2E-02 | -5.15 | Rattus norvegicus vimentin-type intermediate filament associated coiled-coil protein (Vmac), mRNA [NM_001001720] |
| 5 | ***Lmnb1*** | 2.6E-02 | -5.00 | Rattus norvegicus lamin B1 (Lmnb1), mRNA [NM_053905] |
| 6 | ***RT1-M4*** | 4.6E-02 | -4.77 | Rattus norvegicus RT1 class Ib, locus M4 (RT1-M4), mRNA [NM_001168343] |
| 7 | ***Cngb1*** | 3.2E-02 | -4.75 | Rattus norvegicus cyclic nucleotide gated channel beta 1 (Cngb1), mRNA [NM_031809] |
| 8 | ***Mdh1b*** | 3.8E-02 | -4.66 | Rattus norvegicus malate dehydrogenase 1B, NAD (soluble) (Mdh1b), mRNA [NM_001108221] |
| 9 | ***Cyr61*** | 1.9E-03 | -4.55 | Rattus norvegicus cysteine-rich, angiogenic inducer, 61 (Cyr61), mRNA [NM_031327] |
| 10 | ***Prr14l*** | 3.9E-02 | -4.37 | proline rich 14-like [Source:MGI Symbol;Acc:MGI:2443658] [ENSRNOT00000072368] |
| 11 | ***RGD1561667*** | 2.4E-02 | -4.37 | PREDICTED: Rattus norvegicus sperm motility kinase Y-like (RGD1561667), mRNA [XM_003748704] |
| 12 | ***LOC500846*** | 3.9E-02 | -4.17 | Rattus norvegicus hypothetical protein LOC500846 (LOC500846), mRNA [NM_001047962] |
| 13 | ***LOC365499*** | 2.0E-02 | -4.14 | PREDICTED: Rattus norvegicus sperm motility kinase Y-like (LOC365499), transcript variant X3, mRNA [XM_006222919] |
| 14 | ***RGD1562677*** | 3.1E-02 | -4.13 | Rattus norvegicus similar to putative protein kinase (LOC365136), mRNA [XM_344833] |
| 15 | ***Cntn3*** | 3.2E-02 | -4.10 | Rattus norvegicus contactin 3 (plasmacytoma associated) (Cntn3), mRNA [NM_019329] |
| 16 | ***Wnt16*** | 2.2E-02 | -4.08 | Rattus norvegicus wingless-type MMTV integration site family, member 16 (Wnt16), mRNA [NM_001109223] |
| 17 | ***RGD1561667*** | 2.8E-02 | -4.06 | PREDICTED: Rattus norvegicus sperm motility kinase Y-like (RGD1561667), mRNA [XM_003753181] |
| 18 | ***RGD1561667*** | 1.3E-02 | -4.05 | PREDICTED: Rattus norvegicus sperm motility kinase Y-like (RGD1561667), mRNA [XM_003753181] |
| 19 | ***Cx3cr1*** | 1.6E-02 | -4.04 | Rattus norvegicus chemokine (C-X3-C motif) receptor 1 (Cx3cr1), mRNA [NM_133534] |
| 20 | ***Clcn3*** | 3.1E-02 | -4.02 | Rattus norvegicus chloride channel, voltage-sensitive 3 (Clcn3), mRNA [NM_053363] |
| 21 | ***Snapc5*** | 3.7E-02 | -4.01 | Rattus norvegicus small nuclear RNA activating complex, polypeptide 5 (Snapc5), mRNA [NM_001109643] |
| 22 | ***Slc7a12*** | 2.9E-02 | -3.96 | Rattus norvegicus solute carrier family 7 (cationic amino acid transporter, y+ system), member 12 (Slc7a12), mRNA [NM_001011948] |
| 23 | ***Fst*** | 1.8E-02 | -3.92 | Rattus norvegicus follistatin (Fst), mRNA [NM_012561] |
| 24 | ***Esm1*** | 3.4E-02 | -3.86 | Rattus norvegicus endothelial cell-specific molecule 1 (Esm1), mRNA [NM_022604] |
| 25 | ***E4f1*** | 3.4E-02 | -3.86 | Rattus norvegicus E4F transcription factor 1 (E4f1), mRNA [NM_001185046] |
| 26 | ***Usp17l5*** | 2.1E-02 | -3.85 | PREDICTED: Rattus norvegicus ubiquitin specific peptidase 17-like 5 (Usp17l5), mRNA [XM_219062] |
| 27 | ***Mcc*** | 2.4E-02 | -3.80 | Rattus norvegicus mutated in colorectal cancers (Mcc), mRNA [NM_001170534] |
| 28 | ***Rassf2*** | 2.1E-02 | -3.73 | Rattus norvegicus Ras association (RalGDS/AF-6) domain family member 2 (Rassf2), mRNA [NM_001037096] |
| 29 | ***RGD1561185*** | 3.6E-02 | -3.71 | PREDICTED: Rattus norvegicus sperm motility kinase Y-like (RGD1561185), mRNA [XM_006227967] |
| 30 | ***LOC361914*** | 3.1E-02 | -3.71 | Rattus norvegicus similar to solute carrier family 7 (cationic amino acid transporter, y+ system), member 12 (LOC361914), mRNA [NM_001017465] |
| 31 | ***LOC499843*** | 3.8E-02 | -3.70 | Rattus norvegicus LRRGT00091 (LOC499843), mRNA [NM_001047953] |
| 32 | ***Ctf1*** | 2.3E-02 | -3.69 | Rattus norvegicus cardiotrophin 1 (Ctf1), mRNA [NM_017129] |
| 33 | ***LOC365499*** | 3.5E-02 | -3.67 | PREDICTED: Rattus norvegicus sperm motility kinase Y-like (LOC365499), transcript variant X3, mRNA [XM_006222919] |
| 34 | ***LOC685321*** | 3.7E-02 | -3.66 | PREDICTED: Rattus norvegicus serine protease 28-like (LOC685321), mRNA [XM_003750795] |
| 35 | ***Evc2*** | 2.8E-02 | -3.66 | Rattus norvegicus Ellis van Creveld syndrome 2 (Evc2), mRNA [NM_001106012] |
| 36 | ***Extl3*** | 1.9E-02 | -3.65 | Rattus norvegicus exostosin-like glycosyltransferase 3 (Extl3), mRNA [NM_020097] |
| 37 | ***Acn9*** | 2.6E-02 | -3.62 | Rattus norvegicus ACN9 homolog (S. cerevisiae) (Acn9), mRNA [NM_001047914] |
| 38 | ***LOC499219*** | 4.1E-02 | -3.62 | Rattus norvegicus hypothetical protein LOC499219 (LOC499219), mRNA [NM_001047938] |
| 39 | ***LOC300308*** | 1.2E-02 | -3.62 | Rattus norvegicus similar to hypothetical protein 4930509O22 (LOC300308), mRNA [NM_001013952] |
| 40 | ***RGD1565071*** | 9.1E-03 | -3.53 | PREDICTED: Rattus norvegicus sperm motility kinase W-like (RGD1565071), mRNA [XM_001079328] |
| 41 | ***Ackr4*** | 2.3E-02 | -3.50 | PREDICTED: Rattus norvegicus atypical chemokine receptor 4 (Ackr4), transcript variant X2, mRNA [XM_006226544] |
| 42 | ***Ntrk3*** | 1.3E-02 | -3.48 | Rattus norvegicus neurotrophic tyrosine kinase, receptor, type 3 (Ntrk3), transcript variant 1, mRNA [NM_001270656] |
| 43 | ***Cym*** | 2.4E-02 | -3.48 | Rattus norvegicus chymosin (Cym), mRNA [NM_020091] |
| 44 | ***Krt80*** | 2.9E-02 | -3.46 | Rattus norvegicus keratin 80 (Krt80), mRNA [NM_001008815] |
| 45 | ***RGD1309808*** | 2.5E-02 | -3.39 | Rattus norvegicus similar to apolipoprotein L2; apolipoprotein L-II (RGD1309808), mRNA [NM_001134801] |
| 46 | ***LOC305806*** | 9.2E-03 | -3.37 | Rattus norvegicus similar to glutaredoxin 1 (thioltransferase); glutaredoxin (LOC305806), mRNA [NM_001013993] |
| 47 | ***Pigr*** | 4.1E-02 | -3.37 | Rattus norvegicus polymeric immunoglobulin receptor (Pigr), mRNA [NM_012723] |
| 48 | ***Nr4a1*** | 9.6E-03 | -3.36 | Rattus norvegicus nuclear receptor subfamily 4, group A, member 1 (Nr4a1), mRNA [NM_024388] |
| 49 | ***Nepn*** | 4.7E-02 | -3.35 | Rattus norvegicus nephrocan (Nepn), mRNA [NM_001107632] |
| 50 | ***LOC302228*** | 3.2E-02 | -3.35 | PREDICTED: Rattus norvegicus Y-linked testis-specific protein 1-like (LOC302228), mRNA [XM_229842] |
| 51 | ***LOC683469*** | 2.3E-02 | -3.35 | Rattus norvegicus similar to RNA polymerase II transcription factor SIII subunit A2 (Elongin A2) (EloA2) (Transcription elongation factor B polypeptide 3B) (LOC683469), mRNA [NM_001115040] |
| 52 | ***LOC679663*** | 1.5E-02 | -3.34 | PREDICTED: Rattus norvegicus transcription elongation factor B polypeptide 1-like (LOC679663), mRNA [XM_001053910] |
| 53 | ***Fam155b*** | 4.2E-02 | -3.34 | PREDICTED: Rattus norvegicus family with sequence similarity 155, member B (Fam155b), mRNA [XM_001068528] |
| 54 | ***Pinx1*** | 4.7E-02 | -3.34 | Rattus norvegicus PIN2/TERF1 interacting, telomerase inhibitor 1 (Pinx1), mRNA [NM_001083337] |
| 55 | ***LOC100362981*** | 3.2E-02 | -3.32 | Rattus norvegicus LRRGT00010-like (LOC100362981), mRNA [NM_001245924] |
| 56 | ***LOC307263*** | 2.8E-02 | -3.32 | PREDICTED: Rattus norvegicus glyceraldehyde-3-phosphate dehydrogenase-like (LOC307263), misc_RNA [XR_146400] |
| 57 | ***LOC102553316*** | 2.4E-02 | -3.30 | PREDICTED: Rattus norvegicus Y-linked testis-specific protein 1-like (LOC102553316), mRNA [XM_006235107] |
| 58 | ***Ccdc58*** | 4.1E-02 | -3.30 | Rattus norvegicus coiled-coil domain containing 58 (Ccdc58), mRNA [NM_001105875] |
| 59 | ***Krt79*** | 3.8E-04 | -3.29 | PREDICTED: Rattus norvegicus keratin 79 (Krt79), mRNA [XM_003750410] |
| 60 | ***Pigr*** | 4.3E-02 | -3.29 | Rattus norvegicus polymeric immunoglobulin receptor (Pigr), mRNA [NM_012723] |
| 61 | ***LOC681107*** | 4.2E-02 | -3.29 | PREDICTED: Rattus norvegicus Y-linked testis-specific protein 1-like (LOC681107), partial mRNA [XM_003754651] |
| 62 | ***Prm2*** | 4.9E-02 | -3.28 | Rattus norvegicus protamine 2 (Prm2), mRNA [NM_012873] |
| 63 | ***Medag*** | 1.2E-02 | -3.28 | PREDICTED: Rattus norvegicus mesenteric estrogen-dependent adipogenesis (Medag), transcript variant 1, mRNA [XM_001059692] |
| 64 | ***Emc1*** | 3.6E-02 | -3.27 | Rattus norvegicus ER membrane protein complex subunit 1 (Emc1), mRNA [NM_001108690] |
| 65 | ***Pebp4*** | 2.1E-02 | -3.25 | PREDICTED: Rattus norvegicus phosphatidylethanolamine binding protein 4 (Pebp4), mRNA [XM_006222065] |
| 66 | ***Wnt6*** | 3.5E-02 | -3.24 | Rattus norvegicus wingless-type MMTV integration site family, member 6 (Wnt6), mRNA [NM_001108226] |
| 67 | ***RGD1564409*** | 1.4E-02 | -3.24 | PREDICTED: Rattus norvegicus sperm motility kinase W-like (RGD1564409), mRNA [XM_002727434] |
| 68 | ***LOC100366054*** | 4.5E-02 | -3.23 | Rattus norvegicus Da1-10-like (LOC100366054), mRNA [NM_001177830] |
| 69 | ***Ccnk*** | 3.4E-02 | -3.22 | Rattus norvegicus cyclin K (Ccnk), mRNA [NM_001109672] |
| 70 | ***Inhba*** | 7.2E-03 | -3.21 | Rattus norvegicus inhibin beta-A (Inhba), mRNA [NM_017128] |
| 71 | ***Kcng1*** | 4.3E-02 | -3.20 | Rattus norvegicus K+ channel mRNA, sequence. [M81784] |
| 72 | ***Olr1687*** | 4.3E-02 | -3.20 | Rattus norvegicus olfactory receptor 1687 (Olr1687), mRNA [NM_001000508] |
| 73 | ***Lgals7*** | 2.9E-02 | -3.18 | Rattus norvegicus lectin, galactoside-binding, soluble, 7 (Lgals7), mRNA [NM_022582] |
| 74 | ***RGD1560585*** | 2.0E-02 | -3.17 | PREDICTED: Rattus norvegicus high mobility group protein B4-like (RGD1560585), mRNA [XM_006257604] |
| 75 | ***Elf2*** | 3.8E-02 | -3.16 | Rattus norvegicus E74-like factor 2 (Elf2), transcript variant 2, mRNA [NM_001012181] |
| 76 | ***Ablim3*** | 1.9E-03 | -3.16 | Rattus norvegicus actin binding LIM protein family, member 3 (Ablim3), mRNA [NM_001191698] |
| 77 | ***Tmem47*** | 3.7E-02 | -3.16 | Rattus norvegicus transmembrane protein 47 (Tmem47), mRNA [NM_001109317] |
| 78 | ***Nrcam*** | 3.1E-02 | -3.15 | Rattus norvegicus neuronal cell adhesion molecule (Nrcam), mRNA [NM_013150] |
| 79 | ***Ldlrad2*** | 3.2E-02 | -3.14 | PREDICTED: Rattus norvegicus low density lipoprotein receptor class A domain containing 2 (Ldlrad2), mRNA [XM_001070032] |
| 80 | ***LOC691511*** | 3.4E-02 | -3.14 | PREDICTED: Rattus norvegicus vomeronasal type-2 receptor 26-like (LOC691511), mRNA [XM_006223539] |
| 81 | ***Glra2*** | 3.3E-02 | -3.13 | Rattus norvegicus glycine receptor, alpha 2 (Glra2), mRNA [NM_012568] |
| 82 | ***Chrna1*** | 2.0E-02 | -3.12 | Rattus norvegicus cholinergic receptor, nicotinic, alpha 1 (muscle) (Chrna1), mRNA [NM_024485] |
| 83 | ***Olr1411*** | 3.6E-02 | -3.12 | Rattus norvegicus olfactory receptor 1411 (Olr1411), mRNA [NM_001000783] |
| 84 | ***Ucp3*** | 4.2E-04 | -3.11 | Rattus norvegicus uncoupling protein 3 (mitochondrial, proton carrier) (Ucp3), mRNA [NM_013167] |
| 85 | ***Bmp2*** | 1.0E-02 | -3.09 | Rattus norvegicus bone morphogenetic protein 2 (Bmp2), mRNA [NM_017178] |
| 86 | ***Plag1*** | 3.3E-02 | -3.09 | Rattus norvegicus pleiomorphic adenoma gene 1 (Plag1), mRNA [NM_001008316] |
| 87 | ***LOC290595*** | 2.0E-03 | -3.09 | Rattus norvegicus hypothetical gene supported by AF152002 (LOC290595), mRNA [NM_001106063] |
| 88 | ***Idnk*** | 2.2E-02 | -3.08 | Rattus norvegicus idnK, gluconokinase homolog (E. coli) (Idnk), mRNA [NM_001037362] |
| 89 | ***Mfsd1*** | 2.5E-02 | -3.07 | major facilitator superfamily domain containing 1 (Mfsd1), mRNA [Source:RefSeq mRNA;Acc:NM_001191847] [ENSRNOT00000018489] |
| 90 | ***Sox9*** | 4.5E-02 | -3.06 | Protein Sox9; RCG33659 [Source:UniProtKB/TrEMBL;Acc:F1LYL9] [ENSRNOT00000003511] |
| 91 | ***Artn*** | 3.2E-03 | -3.06 | Rattus norvegicus artemin (Artn), mRNA [NM_053397] |
| 92 | ***Npy*** | 5.4E-03 | -3.05 | Rattus norvegicus neuropeptide Y (Npy), mRNA [NM_012614] |
| 93 | ***Pdlim3*** | 8.7E-03 | -3.04 | PDZ and LIM domain 3 (Pdlim3), mRNA [Source:RefSeq mRNA;Acc:NM_053650] [ENSRNOT00000017568] |
| 94 | ***Slc7a10*** | 3.0E-02 | -3.04 | Rattus norvegicus solute carrier family 7 (neutral amino acid transporter light chain, asc system), member 10 (Slc7a10), mRNA [NM_053726] |
| 95 | ***Cflar*** | 2.4E-02 | -3.03 | Rattus norvegicus CASP8 and FADD-like apoptosis regulator, mRNA (cDNA clone MGC:108616 IMAGE:7376876), complete cds. [BC089781] |
| 96 | ***Frmd1*** | 4.1E-02 | -3.03 | PREDICTED: Rattus norvegicus FERM domain containing 1 (Frmd1), mRNA [XM_006227941] |
| 97 | ***Ncoa2*** | 4.4E-02 | -3.03 | Rattus norvegicus nuclear receptor coactivator 2 (Ncoa2), mRNA [NM_031822] |
| 98 | ***LOC363337*** | 1.8E-02 | -3.01 | Rattus norvegicus similar to RIKEN cDNA 1700081O22 (LOC363337), mRNA [NM_001014221] |
| 99 | ***Phf11b*** | 2.9E-02 | -3.01 | Rattus norvegicus PHD finger protein 11B (Phf11b), mRNA [NM_001014235] |
| 100 | ***Csrnp3*** | 3.6E-02 | -3.00 | Rattus norvegicus cysteine-serine-rich nuclear protein 3 (Csrnp3), mRNA [NM_001271225] |
| 101 | ***Olr1369*** | 1.5E-02 | -2.99 | Rattus norvegicus olfactory receptor 1369 (Olr1369), mRNA [NM_001000494] |
| 102 | ***MGC114492*** | 8.8E-03 | -2.99 | Rattus norvegicus similar to melanoma antigen family A, 5 (MGC114492), mRNA [NM_001024895] |
| 103 | ***Olr1700*** | 4.7E-02 | -2.99 | Rattus norvegicus olfactory receptor 1700 (Olr1700), mRNA [NM_001001113] |
| 104 | ***Olr803*** | 3.0E-02 | -2.98 | Rattus norvegicus olfactory receptor 803 (Olr803), mRNA [NM_001000853] |
| 105 | ***Cd99l2*** | 2.8E-02 | -2.98 | Rattus norvegicus CD99 molecule-like 2 (Cd99l2), mRNA [NM_134459] |
| 106 | ***RGD1564177*** | 2.0E-02 | -2.98 | PREDICTED: Rattus norvegicus RGD1564177 (RGD1564177), mRNA [XM_001054841] |
| 107 | ***Tp53i11*** | 6.5E-03 | -2.97 | Rattus norvegicus tumor protein p53 inducible protein 11 (Tp53i11), mRNA [NM_001107749] |
| 108 | ***Fbn2*** | 1.7E-02 | -2.96 | Rattus norvegicus fibrillin 2 (Fbn2), mRNA [NM_031826] |
| 109 | ***Ptprn2*** | 3.9E-02 | -2.95 | Rattus norvegicus protein tyrosine phosphatase, receptor type, N polypeptide 2 (Ptprn2), mRNA [NM_031600] |
| 110 | ***Fdft1*** | 1.7E-02 | -2.94 | Rattus norvegicus farnesyl diphosphate farnesyl transferase 1 (Fdft1), mRNA [NM_019238] |
| 111 | ***Rapgef6*** | 3.6E-02 | -2.94 | Rattus norvegicus Rap guanine nucleotide exchange factor (GEF) 6 (Rapgef6), mRNA [NM_001107003] |
| 112 | ***Timd2*** | 2.4E-02 | -2.94 | Rattus norvegicus T-cell immunoglobulin and mucin domain containing 2 (Timd2), mRNA [NM_001013855] |
| 113 | ***Pura*** | 4.0E-02 | -2.93 | PREDICTED: Rattus norvegicus purine rich element binding protein A (Pura), mRNA [XM_006222540] |
| 114 | ***Smarcc1*** | 2.6E-02 | -2.92 | Rattus norvegicus SWI/SNF related, matrix associated, actin dependent regulator of chromatin, subfamily c, member 1 (Smarcc1), mRNA [NM_001106861] |
| 115 | ***LOC100912652*** | 1.8E-02 | -2.90 | PREDICTED: Rattus norvegicus sperm motility kinase W-like (LOC100912652), partial mRNA [XM_003754694] |
| 116 | ***Dusp26*** | 3.2E-03 | -2.89 | Rattus norvegicus dual specificity phosphatase 26 (putative) (Dusp26), mRNA [NM_001012352] |
| 117 | ***Rmrp*** | 3.4E-02 | -2.89 | Rattus norvegicus RNA component of mitochondrial RNA processing endoribonuclease (Rmrp), RNase MRP RNA [NR_002703] |
| 118 | ***Eml5*** | 8.9E-03 | -2.87 | Rattus norvegicus EML5 (Eml5) mRNA, complete cds. [AY445136] |
| 119 | ***Vom2r67*** | 2.5E-02 | -2.87 | Rattus norvegicus vomeronasal 2 receptor, 67 (Vom2r67), mRNA [NM_001099485] |
| 120 | ***Npy*** | 3.7E-03 | -2.87 | Rattus norvegicus neuropeptide Y (Npy), mRNA [NM_012614] |
| 121 | ***Psmb11*** | 3.2E-02 | -2.86 | Rattus norvegicus proteasome (prosome, macropain) subunit, beta type, 11 (Psmb11), mRNA [NM_001106032] |
| 122 | ***Rnf150*** | 2.2E-02 | -2.86 | Rattus norvegicus ring finger protein 150 (Rnf150), mRNA [NM_001191093] |
| 123 | ***Fam154a*** | 1.8E-02 | -2.85 | Protein FAM154A [Source:UniProtKB/TrEMBL;Acc:M0R6K6] [ENSRNOT00000071318] |
| 124 | ***LOC100912668*** | 2.3E-02 | -2.85 | PREDICTED: Rattus norvegicus uncharacterized LOC100912668 (LOC100912668), misc_RNA [XR_147157] |
| 125 | ***LOC102556298*** | 3.3E-02 | -2.84 | PREDICTED: Rattus norvegicus rho GTPase-activating protein 20-like (LOC102556298), mRNA [XM_006244397] |
| 126 | ***LOC102546376*** | 2.0E-03 | -2.83 | PREDICTED: Rattus norvegicus disks large homolog 5-like (LOC102546376), mRNA [XM_006251678] |
| 127 | ***LOC102550773*** | 4.0E-02 | -2.83 | PREDICTED: Rattus norvegicus rho GTPase-activating protein 20-like (LOC102550773), mRNA [XM_006232463] |
| 128 | ***Nkain3*** | 3.4E-02 | -2.83 | Rattus norvegicus Na+/K+ transporting ATPase interacting 3 (Nkain3), mRNA [NM_001109540] |
| 129 | ***LOC100912668*** | 2.4E-02 | -2.82 | PREDICTED: Rattus norvegicus uncharacterized LOC100912668 (LOC100912668), misc_RNA [XR_147157] |
| 130 | ***LOC501437*** | 4.7E-02 | -2.81 | PREDICTED: Rattus norvegicus CD99 antigen-like protein 2-like (LOC501437), mRNA [XM_006227067] |
| 131 | ***Tas2r120*** | 3.6E-02 | -2.81 | Rattus norvegicus taste receptor, type 2, member 120 (Tas2r120), mRNA [NM_001080937] |
| 132 | ***Cxxc4*** | 1.8E-02 | -2.79 | Rattus norvegicus CXXC finger protein 4 (Cxxc4), mRNA [NM_053342] |
| 133 | ***Dnah10*** | 3.2E-02 | -2.78 | PREDICTED: Rattus norvegicus dynein, axonemal, heavy chain 10 (Dnah10), mRNA [XM_006221420] |
| 134 | ***Ctrb1*** | 3.2E-02 | -2.77 | Rattus norvegicus chymotrypsinogen B1 (Ctrb1), mRNA [NM_012536] |
| 135 | ***Mrpl10*** | 3.4E-02 | -2.77 | Rattus norvegicus mitochondrial ribosomal protein L10 (Mrpl10), mRNA [NM_001109620] |
| 136 | ***Cyp4a8*** | 3.4E-02 | -2.76 | Rattus norvegicus cytochrome P450, family 4, subfamily a, polypeptide 8 (Cyp4a8), mRNA [NM_031605] |
| 137 | ***Tp63*** | 3.5E-02 | -2.76 | Rattus norvegicus tumor protein p63 (Tp63), transcript variant 3, mRNA [NM_001127341] |
| 138 | ***Clec14a*** | 2.7E-02 | -2.75 | Rattus norvegicus C-type lectin domain family 14, member A (Clec14a), mRNA [NM_001014077] |
| 139 | ***Cdx1*** | 3.3E-02 | -2.75 | PREDICTED: Rattus norvegicus caudal type homeo box 1 (Cdx1), partial mRNA [XM_006254816] |
| 140 | ***Gimap9*** | 3.5E-02 | -2.74 | Rattus norvegicus GTPase, IMAP family member 9 (Gimap9), mRNA [NM_001008398] |
| 141 | ***Asb15*** | 2.0E-02 | -2.73 | Protein Asb15 [Source:UniProtKB/TrEMBL;Acc:F1LLX0] [ENSRNOT00000008917] |
| 142 | ***Ccr9*** | 3.5E-02 | -2.72 | Rattus norvegicus chemokine (C-C motif) receptor 9 (Ccr9), mRNA [NM_172329] |
| 143 | ***Igtp*** | 2.8E-02 | -2.71 | Rattus norvegicus interferon gamma induced GTPase (Igtp), mRNA [NM_001008765] |
| 144 | ***Olr374*** | 4.1E-02 | -2.71 | Rattus norvegicus olfactory receptor 374 (Olr374), mRNA [NM_001001289] |
| 145 | ***Gabarapl2*** | 2.1E-02 | -2.71 | Rattus norvegicus GABA(A) receptor-associated protein like 2 (Gabarapl2), mRNA [NM_022706] |
| 146 | ***Elfn2-ps1*** | 4.2E-02 | -2.70 | PREDICTED: Rattus norvegicus protein phosphatase 1 regulatory subunit 29-like (LOC100910536), mRNA [XM_006242041] |
| 147 | ***Shbg*** | 8.6E-03 | -2.69 | Rattus norvegicus sex hormone binding globulin (Shbg), mRNA [NM_012650] |
| 148 | ***LOC102557071*** | 2.6E-02 | -2.68 | PREDICTED: Rattus norvegicus CD99 antigen-like protein 2-like (LOC102557071), mRNA [XM_006227023] |
| 149 | ***LOC100912880*** | 3.5E-02 | -2.67 | PREDICTED: Rattus norvegicus uncharacterized LOC100912880 (LOC100912880), transcript variant X5, ncRNA [XR_342602] |
| 150 | ***Sdpr*** | 2.6E-02 | -2.65 | Rattus norvegicus serum deprivation response (Sdpr), mRNA [NM_001007712] |
| 151 | ***LOC102547093*** | 4.5E-03 | -2.64 | PREDICTED: Rattus norvegicus uncharacterized LOC102547093 (LOC102547093), mRNA [XM_006251682] |
| 152 | ***Rab6b*** | 9.2E-04 | -2.64 | Rattus norvegicus RAB6B, member RAS oncogene family (Rab6b), mRNA [NM_001108775] |
| 153 | ***Adcy9*** | 1.9E-03 | -2.62 | adenylate cyclase 9 (Adcy9), mRNA [Source:RefSeq mRNA;Acc:NM_001106980] [ENSRNOT00000072802] |
| 154 | ***LOC365085*** | 2.3E-02 | -2.61 | PREDICTED: Rattus norvegicus nidogen-2-like (LOC365085), mRNA [XM_006232465] |
| 155 | ***Fxyd7*** | 2.7E-02 | -2.61 | Rattus norvegicus FXYD domain-containing ion transport regulator 7 (Fxyd7), mRNA [NM_022008] |
| 156 | ***Tcf7l2*** | 1.9E-02 | -2.61 | Rattus norvegicus transcription factor 7-like 2 (T-cell specific, HMG-box) (Tcf7l2), mRNA [NM_001191052] |
| 157 | ***Ooep*** | 8.5E-04 | -2.60 | PREDICTED: Rattus norvegicus oocyte expressed protein (Ooep), misc_RNA [XR_348628] |
| 158 | ***Ooep*** | 6.2E-05 | -2.60 | PREDICTED: Rattus norvegicus oocyte expressed protein (Ooep), misc_RNA [XR_348628] |
| 159 | ***LOC100909456*** | 3.6E-02 | -2.58 | PREDICTED: Rattus norvegicus uncharacterized LOC100909456 (LOC100909456), ncRNA [XR_344941] |
| 160 | ***Lpl*** | 2.0E-02 | -2.58 | Rattus norvegicus lipoprotein lipase (Lpl), mRNA [NM_012598] |
| 161 | ***Olr337*** | 1.9E-02 | -2.58 | Rattus norvegicus olfactory receptor 337 (Olr337), mRNA [NM_001000507] |
| 162 | ***Bend5*** | 2.3E-04 | -2.57 | Rattus norvegicus BEN domain containing 5 (Bend5), mRNA [NM_001108672] |
| 163 | ***Snurf*** | 3.3E-02 | -2.56 | Rattus norvegicus SNRPN upstream reading frame protein (SNURF) mRNA, complete cds. [AF101041] |
| 164 | ***Anxa8*** | 6.8E-03 | -2.55 | Rattus norvegicus annexin A8 (Anxa8), mRNA [NM_001031654] |
| 165 | ***RGD1562865*** | 3.3E-02 | -2.55 | Rattus norvegicus similar to BTB and CNC homology 1, basic leucine zipper transcription factor 2 (RGD1562865), mRNA [NM_001135754] |
| 166 | ***Cwc22*** | 6.1E-03 | -2.54 | Protein Cwc22 [Source:UniProtKB/TrEMBL;Acc:F1LY38] [ENSRNOT00000037684] |
| 167 | ***LOC363324*** | 1.6E-02 | -2.54 | PREDICTED: Rattus norvegicus uncharacterized LOC363324 (LOC363324), transcript variant X1, mRNA [XM_006255427] |
| 168 | ***Clcn6*** | 4.9E-02 | -2.54 | Rattus norvegicus chloride channel, voltage-sensitive 6 (Clcn6), mRNA [NM_001106479] |
| 169 | ***LOC685067*** | 1.3E-02 | -2.54 | guanylate binding protein 7 [Source:MGI Symbol;Acc:MGI:2444421] [ENSRNOT00000037181] |
| 170 | ***C1ql3*** | 4.5E-02 | -2.54 | Rattus norvegicus complement component 1, q subcomponent-like 3 (C1ql3), mRNA [NM_001109403] |
| 171 | ***Hic2*** | 4.2E-02 | -2.53 | Rattus norvegicus hypermethylated in cancer 2 (Hic2), mRNA [NM_001105862] |
| 172 | ***Greb1*** | 3.8E-02 | -2.53 | gene regulated by estrogen in breast cancer protein [Source:MGI Symbol;Acc:MGI:2149712] [ENSRNOT00000032417] |
| 173 | ***LOC102549564*** | 1.3E-02 | -2.52 | PREDICTED: Rattus norvegicus disks large homolog 5-like (LOC102549564), transcript variant X1, mRNA [XM_006227114] |
| 174 | ***Ttc4*** | 1.8E-02 | -2.52 | Rattus norvegicus tetratricopeptide repeat domain 4 (Ttc4), mRNA [NM_001013214] |
| 175 | ***Sycp2*** | 2.2E-02 | -2.52 | Rattus norvegicus synaptonemal complex protein 2 (Sycp2), mRNA [NM_130735] |
| 176 | ***Abca8*** | 3.3E-04 | -2.52 | PREDICTED: Rattus norvegicus ATP-binding cassette, subfamily A (ABC1), member 8 (Abca8), mRNA [XM_006221030] |
| 177 | ***Efnb3*** | 3.1E-02 | -2.50 | Rattus norvegicus ephrin B3 (Efnb3), mRNA [NM_001100980] |
| 178 | ***Nrxn1*** | 1.1E-02 | -2.49 | Rattus norvegicus neurexin 1 (Nrxn1), mRNA [NM_021767] |
| 179 | ***Piezo2*** | 7.3E-04 | -2.49 | PREDICTED: Rattus norvegicus piezo-type mechanosensitive ion channel component 2 (Piezo2), transcript variant X2, mRNA [XM_006222590] |
| 180 | ***C1qtnf2*** | 9.4E-03 | -2.49 | Rattus norvegicus C1q and tumor necrosis factor related protein 2 (C1qtnf2), mRNA [NM_001191918] |
| 181 | ***Chm*** | 1.7E-02 | -2.49 | Rattus norvegicus choroideremia (Rab escort protein 1) (Chm), mRNA [NM_017067] |
| 182 | ***Tp53*** | 3.7E-02 | -2.48 | Rattus norvegicus tumor protein p53 (Tp53), mRNA [NM_030989] |
| 183 | ***Lmcd1*** | 1.9E-02 | -2.48 | Rattus norvegicus LIM and cysteine-rich domains 1 (Lmcd1), mRNA [NM_001008562] |
| 184 | ***Kctd4*** | 1.8E-02 | -2.47 | Rattus norvegicus potassium channel tetramerization domain containing 4 (Kctd4), mRNA [NM_001109650] |
| 185 | ***Ripply3*** | 2.7E-04 | -2.47 | Rattus norvegicus ripply transcriptional repressor 3 (Ripply3), mRNA [NM_001105892] |
| 186 | ***Kank2*** | 1.9E-02 | -2.46 | Rattus norvegicus KN motif and ankyrin repeat domains 2 (Kank2), mRNA [NM_001270413] |
| 187 | ***Tecpr1*** | 2.2E-02 | -2.45 | Rattus norvegicus tectonin beta-propeller repeat containing 1 (Tecpr1), mRNA [NM_001037191] |
| 188 | ***Med12*** | 2.9E-02 | -2.43 | Rattus norvegicus mediator complex subunit 12 (Med12), mRNA [NM_001193292] |
| 189 | ***Zdhhc5*** | 2.3E-02 | -2.43 | Rattus norvegicus zinc finger, DHHC-type containing 5 (Zdhhc5), mRNA [NM_001039338] |
| 190 | ***Dusp8*** | 2.9E-04 | -2.42 | Rattus norvegicus dual specificity phosphatase 8 (Dusp8), mRNA [NM_001108510] |
| 191 | ***Ccnjl*** | 4.1E-02 | -2.42 | Rattus norvegicus cyclin J-like (Ccnjl), mRNA [NM_001037773] |
| 192 | ***RGD1562204*** | 3.7E-02 | -2.41 | PREDICTED: Rattus norvegicus 60S ribosomal protein L9-like (RGD1562204), misc_RNA [XR_146222] |
| 193 | ***Satb1*** | 3.4E-05 | -2.40 | Rattus norvegicus SATB homeobox 1 (Satb1), mRNA [NM_001012129] |
| 194 | ***Akap5*** | 2.2E-03 | -2.39 | Rattus norvegicus A kinase (PRKA) anchor protein 5 (Akap5), mRNA [NM_133515] |
| 195 | ***LOC691195*** | 3.3E-02 | -2.38 | PREDICTED: Rattus norvegicus 60S ribosomal protein L21-like (LOC691195), mRNA [XM_006224236] |
| 196 | ***Lphn2*** | 9.2E-03 | -2.36 | Rattus norvegicus latrophilin 2 (Lphn2), transcript variant 2, mRNA [NM_001190475] |
| 197 | ***R3hdm4*** | 4.7E-02 | -2.36 | Rattus norvegicus R3H domain containing 4 (R3hdm4), mRNA [NM_001173974] |
| 198 | ***Hmg1l1*** | 1.4E-02 | -2.34 | Rattus norvegicus high-mobility group (nonhistone chromosomal) protein 1-like 1 (Hmg1l1), mRNA [NM_001109373] |
| 199 | ***Gbp5*** | 3.6E-04 | -2.34 | Rattus norvegicus guanylate binding protein 5 (Gbp5), mRNA [NM_001108569] |
| 200 | ***Opn4*** | 3.8E-02 | -2.33 | Rattus norvegicus opsin 4 (Opn4), mRNA [NM_138860] |
| 201 | ***LOC102547093*** | 5.1E-03 | -2.32 | PREDICTED: Rattus norvegicus uncharacterized LOC102547093 (LOC102547093), transcript variant X3, mRNA [XM_006227098] |
| 202 | ***Kcnmb2*** | 1.2E-02 | -2.32 | Rattus norvegicus potassium large conductance calcium-activated channel, subfamily M, beta member 2 (Kcnmb2), mRNA [NM_176861] |
| 203 | ***LOC685438*** | 1.2E-02 | -2.32 | PREDICTED: Rattus norvegicus paired immunoglobulin-like type 2 receptor beta-2-like (LOC685438), transcript variant X2, mRNA [XM_006221313] |
| 204 | ***Gp1bb*** | 2.3E-02 | -2.31 | Rattus norvegicus glycoprotein Ib (platelet), beta polypeptide (Gp1bb), mRNA [NM_053930] |
| 205 | ***Cdc37*** | 2.4E-02 | -2.31 | Rattus norvegicus cell division cycle 37 (Cdc37), mRNA [NM_053743] |
| 206 | ***Acta2*** | 3.1E-03 | -2.31 | Rattus norvegicus actin, alpha 2, smooth muscle, aorta (Acta2), mRNA [NM_031004] |
| 207 | ***Tmem169*** | 2.8E-02 | -2.30 | Rattus norvegicus transmembrane protein 169 (Tmem169), mRNA [NM_001109574] |
| 208 | ***Zfp978*** | 3.9E-02 | -2.30 | Rattus norvegicus zinc finger protein 978 (Zfp978), mRNA [NM_001025677] |
| 209 | ***LOC102548695*** | 3.8E-02 | -2.30 | PREDICTED: Rattus norvegicus zinc finger protein 45-like (LOC102548695), mRNA [XM_006223125] |
| 210 | ***LOC102549144*** | 4.5E-02 | -2.30 | PREDICTED: Rattus norvegicus uncharacterized LOC102549144 (LOC102549144), transcript variant X1, ncRNA [XR_348123] |
| 211 | ***Epha4*** | 7.7E-03 | -2.29 | Rattus norvegicus Eph receptor A4 (Epha4), mRNA [NM_001162411] |
| 212 | ***RGD1310587*** | 2.0E-04 | -2.29 | Rattus norvegicus similar to hypothetical protein FLJ14146 (RGD1310587), mRNA [NM_001100857] |
| 213 | ***Adamts1*** | 4.6E-02 | -2.27 | Rattus norvegicus ADAM metallopeptidase with thrombospondin type 1 motif, 1 (Adamts1), mRNA [NM_024400] |
| 214 | ***Gimap1*** | 7.2E-05 | -2.27 | Rattus norvegicus GTPase, IMAP family member 1 (Gimap1), mRNA [NM_001034849] |
| 215 | ***Dclk2*** | 2.3E-03 | -2.26 | Rattus norvegicus doublecortin-like kinase 2 (Dclk2), transcript variant 2, mRNA [NM_001009691] |
| 216 | ***LOC687797*** | 3.5E-02 | -2.26 | PREDICTED: Rattus norvegicus similar to tumor suppressor candidate 5 (LOC687797), transcript variant X1, mRNA [XM_001080179] |
| 217 | ***Hoxa9*** | 2.7E-02 | -2.26 | Rattus norvegicus homeobox A9 (Hoxa9), mRNA [NM_001109233] |
| 218 | ***Enpp1*** | 8.8E-05 | -2.26 | Rattus norvegicus ectonucleotide pyrophosphatase/phosphodiesterase 1 (Enpp1), mRNA [NM_053535] |
| 219 | ***Dtwd1*** | 2.2E-02 | -2.26 | Rattus norvegicus DTW domain containing 1 (Dtwd1), mRNA [NM_001013921] |
| 220 | ***Rgs7bp*** | 2.0E-02 | -2.25 | Rattus norvegicus regulator of G-protein signaling 7 binding protein (Rgs7bp), mRNA [NM_001012347] |
| 221 | ***Tceb1*** | 3.6E-02 | -2.25 | Rattus norvegicus transcription elongation factor B (SIII), polypeptide 1 (Tceb1), transcript variant 1, mRNA [NM_001270561] |
| 222 | ***Piezo2*** | 4.7E-03 | -2.24 | piezo-type mechanosensitive ion channel component 2 [Source:MGI Symbol;Acc:MGI:1918781] [ENSRNOT00000065693] |
| 223 | ***Sstr4*** | 6.3E-03 | -2.24 | Rattus norvegicus somatostatin receptor 4 (Sstr4), mRNA [NM_013036] |
| 224 | ***Mylk*** | 1.8E-03 | -2.24 | Rattus norvegicus myosin light chain kinase (Mylk), mRNA [NM_001105874] |
| 225 | ***Mapt*** | 1.3E-02 | -2.23 | Rattus norvegicus microtubule-associated protein tau (Mapt), mRNA [NM_017212] |
| 226 | ***Ptprr*** | 4.1E-04 | -2.23 | Rattus norvegicus protein tyrosine phosphatase, receptor type, R (Ptprr), transcript variant 1, mRNA [NM_053594] |
| 227 | ***Meox2*** | 3.5E-04 | -2.23 | Rattus norvegicus mesenchyme homeobox 2 (Meox2), mRNA [NM_017149] |
| 228 | ***Klf4*** | 1.9E-03 | -2.22 | Rattus norvegicus Kruppel-like factor 4 (gut) (Klf4), mRNA [NM_053713] |
| 229 | ***She*** | 4.0E-03 | -2.22 | PREDICTED: Rattus norvegicus Src homology 2 domain containing E (She), mRNA [XM_001062249] |
| 230 | ***RGD1563888*** | 2.8E-02 | -2.21 | uncharacterized protein LOC360692 [Source:RefSeq peptide;Acc:NP_001101782] [ENSRNOT00000002119] |
| 231 | ***Slc9a3r2*** | 1.1E-02 | -2.21 | Rattus norvegicus solute carrier family 9, subfamily A (NHE3, cation proton antiporter 3), member 3 regulator 2 (Slc9a3r2), mRNA [NM_053811] |
| 232 | ***Nbl1*** | 2.0E-02 | -2.21 | Rattus norvegicus neuroblastoma 1, DAN family BMP antagonist (Nbl1), mRNA [NM_031609] |
| 233 | ***Dusp14*** | 8.4E-06 | -2.20 | Rattus norvegicus dual specificity phosphatase 14 (Dusp14), transcript variant 1, mRNA [NM_001079893] |
| 234 | ***RGD1566399*** | 8.8E-03 | -2.19 | PREDICTED: Rattus norvegicus histone acetyltransferase KAT6B-like (RGD1566399), mRNA [XM_006251674] |
| 235 | ***LOC680964*** | 1.6E-02 | -2.19 | PREDICTED: Rattus norvegicus non-histone chromosomal protein HMG-14-like (LOC680964), mRNA [XM_001059675] |
| 236 | ***RGD1306151*** | 2.4E-03 | -2.18 | Rattus norvegicus similar to hypothetical protein DKFZp761D0211 (RGD1306151), mRNA [NM_001108652] |
| 237 | ***Ccdc88a*** | 1.1E-02 | -2.17 | PREDICTED: Rattus norvegicus coiled coil domain containing 88A (Ccdc88a), transcript variant X2, mRNA [XM_006221877] |
| 238 | ***Kcnb1*** | 3.5E-03 | -2.16 | Rattus norvegicus potassium voltage gated channel, Shab-related subfamily, member 1 (Kcnb1), mRNA [NM_013186] |
| 239 | ***Cdc42bpg*** | 1.0E-02 | -2.16 | Rattus norvegicus CDC42 binding protein kinase gamma (DMPK-like) (Cdc42bpg), mRNA [NM_001130013] |
| 240 | ***Gsg1l*** | 8.2E-05 | -2.15 | PREDICTED: Rattus norvegicus GSG1-like (Gsg1l), mRNA [XM_002725684] |
| 241 | ***LOC102556999*** | 3.3E-02 | -2.15 | PREDICTED: Rattus norvegicus uncharacterized LOC102556999 (LOC102556999), mRNA [XM_006227399] |
| 242 | ***Tbx2*** | 1.8E-03 | -2.14 | Rattus norvegicus T-box 2 (Tbx2), mRNA [NM_001107033] |
| 243 | ***Supt20*** | 2.1E-02 | -2.14 | Rattus norvegicus suppressor of Ty 20 (Supt20), mRNA [NM_001014170] |
| 244 | ***Gimap4*** | 1.0E-02 | -2.14 | Rattus norvegicus GTPase, IMAP family member 4 (Gimap4), mRNA [NM_173153] |
| 245 | ***Ereg*** | 2.2E-03 | -2.14 | Rattus norvegicus epiregulin (Ereg), mRNA [NM_021689] |
| 246 | ***Clcf1*** | 9.1E-03 | -2.13 | Rattus norvegicus cardiotrophin-like cytokine factor 1 (Clcf1), mRNA [NM_207615] |
| 247 | ***Ahnak*** | 7.8E-03 | -2.13 | Rattus norvegicus AHNAK nucleoprotein (Ahnak), mRNA [NM_001191951] |
| 248 | ***Mef2c*** | 3.3E-02 | -2.13 | PREDICTED: Rattus norvegicus myocyte enhancer factor 2C (Mef2c), transcript variant X1, mRNA [XM_006223955] |
| 249 | ***Kif26a*** | 3.0E-02 | -2.13 | Rattus norvegicus kinesin family member 26A (Kif26a), mRNA [NM_001170348] |
| 250 | ***Cyyr1*** | 7.8E-03 | -2.11 | Rattus norvegicus cysteine/tyrosine-rich 1 (Cyyr1), mRNA [NM_001013980] |
| 251 | ***Ppp1r14c*** | 2.7E-02 | -2.10 | Rattus norvegicus protein phosphatase 1, regulatory (inhibitor) subunit 14c (Ppp1r14c), mRNA [NM_133425] |
| 252 | ***Lrp5*** | 5.0E-03 | -2.10 | Rattus norvegicus low density lipoprotein receptor-related protein 5 (Lrp5), mRNA [NM_001106321] |
| 253 | ***Pcdh17*** | 1.6E-03 | -2.10 | Rattus norvegicus protocadherin 17 (Pcdh17), mRNA [NM_001107279] |
| 254 | ***Cdh13*** | 2.4E-02 | -2.09 | Rattus norvegicus cadherin 13 (Cdh13), mRNA [NM_138889] |
| 255 | ***Jade1*** | 1.6E-02 | -2.09 | Rattus norvegicus jade family PHD finger 1 (Jade1), mRNA [NM_001107670] |
| 256 | ***Sox5*** | 1.3E-02 | -2.08 | Rattus norvegicus SRY (sex determining region Y)-box 5 (Sox5), mRNA [NM_001271267] |
| 257 | ***RT1-T24-1*** | 2.3E-03 | -2.08 | Rattus norvegicus RT1 class I, locus T24, gene 1 (RT1-T24-1), mRNA [NM_001008858] |
| 258 | ***Slc38a5*** | 2.7E-02 | -2.06 | Rattus norvegicus solute carrier family 38, member 5 (Slc38a5), mRNA [NM_138854] |
| 259 | ***Mllt3*** | 2.4E-04 | -2.06 | Rattus norvegicus myeloid/lymphoid or mixed-lineage leukemia (trithorax homolog, Drosophila); translocated to, 3 (Mllt3), mRNA [NM_053718] |
| 260 | ***Anln*** | 4.4E-02 | -2.06 | PREDICTED: Rattus norvegicus anillin, actin binding protein (Anln), mRNA [XM_006242714] |
| 261 | ***Lgr4*** | 1.0E-02 | -2.05 | Rattus norvegicus leucine-rich repeat-containing G protein-coupled receptor 4 (Lgr4), mRNA [NM_173328] |
| 262 | ***Nkain4*** | 8.3E-03 | -2.05 | Rattus norvegicus Na+/K+ transporting ATPase interacting 4 (Nkain4), mRNA [NM_001106550] |
| 263 | ***Igf2*** | 1.1E-02 | -2.04 | Rattus norvegicus insulin-like growth factor 2 (Igf2), transcript variant 1, mRNA [NM_031511] |
| 264 | ***Acsm5*** | 3.0E-02 | -2.03 | Rattus norvegicus acyl-CoA synthetase medium-chain family member 5 (Acsm5), mRNA [NM_001014162] |
| 265 | ***Tcf4*** | 4.2E-02 | -2.03 | Rattus norvegicus transcription factor 4 (Tcf4), mRNA [NM_053369] |
| 266 | ***Katnal1*** | 3.3E-02 | -2.03 | Rattus norvegicus katanin p60 subunit A-like 1 (Katnal1), mRNA [NM_001006956] |
| 267 | ***Itga9*** | 2.8E-03 | -2.02 | PREDICTED: Rattus norvegicus integrin, alpha 9 (Itga9), mRNA [XM_006244173] |
| 268 | ***Ube4b*** | 2.9E-02 | -2.02 | Rattus norvegicus ubiquitination factor E4B (Ube4b), mRNA [NM_001271198] |
| 269 | ***Dsp*** | 4.4E-04 | -2.02 | Protein Dsp [Source:UniProtKB/TrEMBL;Acc:F1LMV6] [ENSRNOT00000018649] |
| 270 | ***Sec14l3*** | 2.4E-02 | -2.02 | Rattus norvegicus SEC14-like 3 (S. cerevisiae) (Sec14l3), mRNA [NM_022608] |
| 271 | ***LOC691642*** | 1.4E-03 | -2.01 | PREDICTED: Rattus norvegicus similar to high mobility group nucleosomal binding domain 1 (LOC691642), mRNA [XM_001079135] |
| 272 | ***Ramp1*** | 2.6E-02 | -2.01 | Rattus norvegicus receptor (G protein-coupled) activity modifying protein 1 (Ramp1), mRNA [NM_031645] |
| 273 | ***Cdk17*** | 1.0E-03 | -2.00 | Rattus norvegicus cyclin-dependent kinase 17 (Cdk17), mRNA [NM_001108082] |
| 274 | ***Lphn1*** | 1.2E-02 | -2.00 | Rattus norvegicus latrophilin 1 (Lphn1), mRNA [NM_022962] |
