# Supplementary figures and images for: Microarray analysis of gene expression in lung tissues of indium-exposed rats: possible roles of S100 proteins in lung diseases
Source: Arch Toxicol. 2024 Nov 8;99(1):245–58. doi: 10.1007/s00204-024-03897-x (PMC11742277; doi:10.1007/s00204-024-03897-x)

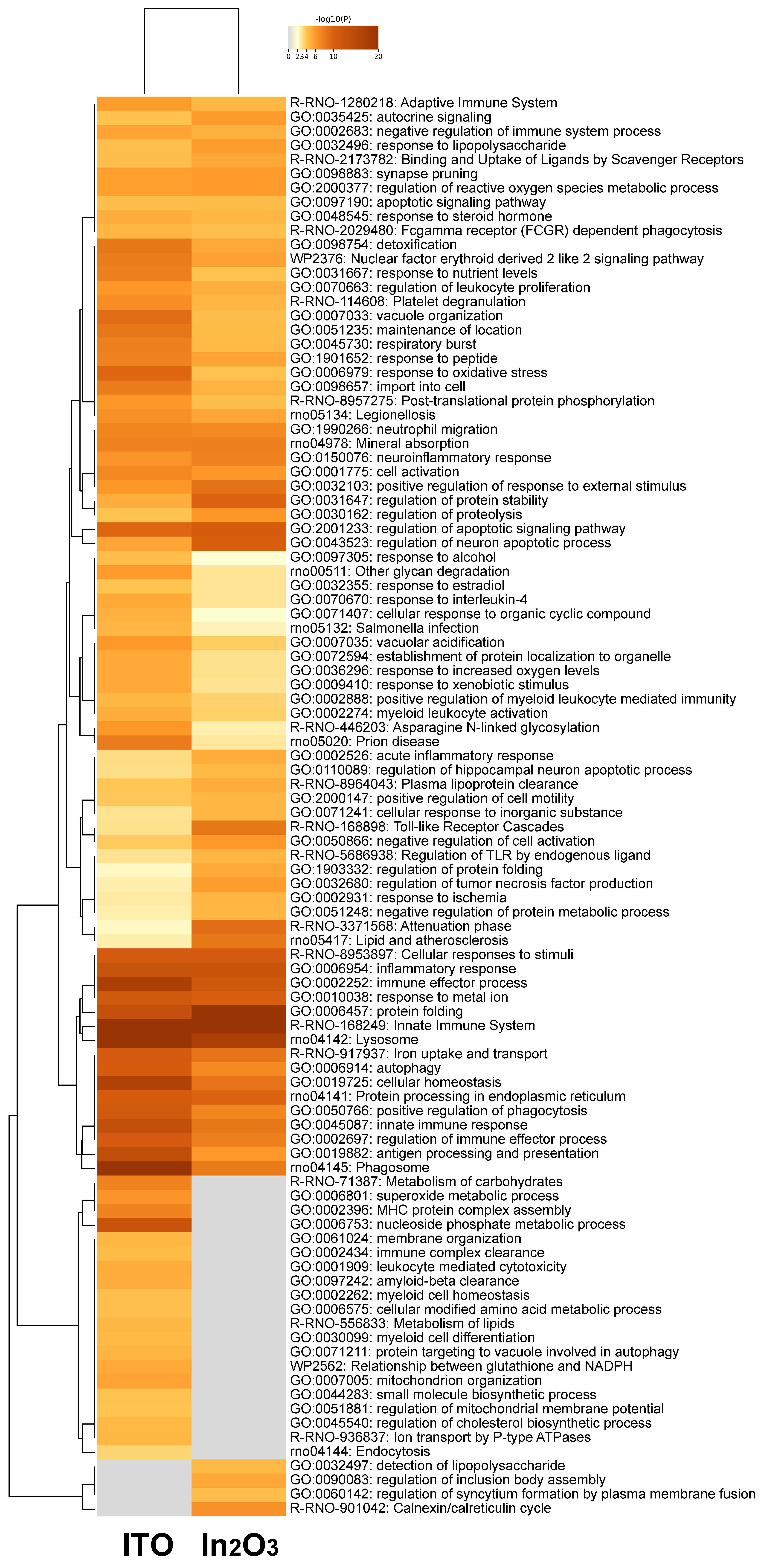

Supplement: Supplementary file 3 — Supplementary file3 (JPG 3985 KB) [file 204_2024_3897_MOESM3_ESM.jpg]
